# Updating the forelimb anatomy of the domestic cat (*Felis catus*, Felidae) based on evolutionary inferences of its muscles and nerves I: Shoulder and brachium

**DOI:** 10.1111/joa.70151

**Published:** 2026-04-19

**Authors:** Juan Fernando Vélez García, Ismael Vélez Cabrera, Maria Angelica Miglino

**Affiliations:** ^1^ Research Group of Medicine and Surgery in Small Animals, Department of Animal Health, Faculty of Veterinary Medicine and Animal Science Universidad del Tolima Ibagué Colombia; ^2^ Postgraduate Program in Anatomy of the Domestic and Wild Animals, Department of Surgery, School of Veterinary Medicine and Animal Science Universidade de São Paulo São Paulo Brazil; ^3^ Instituto Educativo Superior Sanje (IES Sanje) Murcia Spain; ^4^ University of Marilia ‐ UNIMAR Marília Brazil

**Keywords:** anatomical variants, artery, muscle maps, myology, nerve, thoracic limb

## Abstract

The domestic cat (*Felis catus*) is a species of the order Carnivora, suborder Feliformia, and family Felidae. It is used as a representative model of mammals in comparative anatomy of vertebrates, whereas in veterinary anatomy, only differences from the domestic dog are described. Because of this, several recent studies on thoracic limb muscles in wild felids have preferred the use of former references concerning the anatomy of the cat as a guide to describe their findings. Furthermore, these studies established the homologies of the thoracic limb muscles among felids based on topology but did not consider other evolutionary characteristics, such as innervation and embryological development. Therefore, the present study aimed to describe in detail the thoracic limb muscles and the brachial plexus of *F. catus* to reconstruct muscle maps, report anatomical variants, establish homologies, and infer muscle derivations in felids. In this chapter, we report the attachments, innervation, and arterial supply of the intrinsic shoulder and brachium muscles and the origin and distribution of the brachial plexus of *F. catus* in these regions. Nineteen thoracic limbs from 10 carcasses of *F. catus* were used. Differences from other studies in *F. catus* and other felids were observed when considering the topology, innervation, and most recent available literature on the embryonic muscle splitting patterns in amniotes. Among the main findings, we observed the articularis humeri muscle in only one limb (1/19), exhibiting the arrangement of an atavistic muscle, similar to the coracobrachialis longus muscle observed in six limbs (6/19) and the caput breve of biceps brachii muscle observed in two limbs (2/19). The triceps brachii muscle only has four heads, and the anconeus medialis muscle (named by other authors as the short portion of the caput mediale, caput mediale accessorium, or m. epitrochleoanconeus) is not part of the triceps brachii muscle since its attachments are independent, the ulnar nerve innervates it, and the most recent embryological derivation evidence in mammals supports it. Two tricipital bursae were observed between the tendons of the triceps brachii heads. The muscle maps on the thoracic limb bones were reconstructed and complemented with the anatomical relationships of the ligaments, joint capsules, synovial bursae, and nerves. Most studied brachial plexuses originated from C6‐T1 (13/16), whereas the origin from C5 was observed bilaterally in two limbs and from T2 in one limb. Adding the results of other studies (56), the contributions from C5 or T2 were 4.17% (3/72) and 1.39% (1/72), respectively. The distribution of the brachial plexus nerves of *F. catus* was similar to that reported in other studies in felids, although most of those studies did not report innervation to the anconeus medialis muscle. The nerve distribution and muscle topology allowed us to establish muscle homologies with other felids and infer evolutionary derivations of the shoulder and brachium muscles in the family Felidae. In conclusion, the present study updated the anatomy of the shoulder and brachium in *F. catus* and should be considered in evolutionary studies in Carnivora and diagnoses and surgical approaches in veterinary medicine of felids.

## Graphical abstract

In this study, we provide a detailed description of the shoulder and brachium muscles and the brachial plexus of the domestic cat (*Felis catus*). We identified muscular variants (articularis humeri, coracobrachialis longus, biceps brachii caput breve), clarified the independence of the anconeus medialis muscle from the triceps brachii muscle, and documented variations in the origin and distribution of the brachial plexus nerves. These findings refine muscle homologies within Felidae and allowed us to infer their evolutionary derivations.

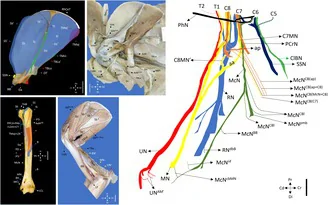

## 1 INTRODUCTION

The domestic cat (*Felis catus*) is a mammal species of the order Carnivora, suborder Feliformia, and family Felidae (Hassanin et al., 2021; Nyakatura & Bininda‐Emonds, 2012). The suborder Feliformia diverged from Carnivora in the early Eocene (Paleogene) approximately 52.7–46.7 million years ago (Mya), the family Felidae in the Oligocene (Paleogene) approximately 27.4–24.7 Mya, and the *F. catus* species approximately 0.4 Mya (Hassanin et al., 2021). The thoracic limb (forelimb) of the family Felidae evolved into a digitigrade support to carry out more efficient terrestrial locomotion, and it also has distal interphalangeal joints adapted to have retractile nails that allow them to climb trees. Thus, the felids are classified as species with scansorial locomotion, except for the cheetah (*Acinonyx jubatus*), which is a strictly terrestrial (cursorial) species and does not have retractile nails (Cuff et al., 2016; Gonyea & Ashworth, 1975). *Felis catus* is a species considered domestic because of its adaptation to live with humans (Serpell, 2013), and within the family Felidae, it is the species with the most anatomical studies. It is a representative model of mammals in terms of comparative anatomy of vertebrates (De Iuliis & Pulerà, 2019; Sebastiani & Fishbeck, 2005), whereas in veterinary anatomy, it is secondary, and only differences from the domestic dog (*Canis lupus familiaris*) have been described (Barone, 2020; Done et al., 2009; Getty, 1982; Liebich et al., 2020; Singh, 2018). There are specific veterinary anatomy textbooks for *F. catus* (Gil Cano et al., 2023; Hudson & Hamilton, 2010; König & Pérez, 2022); however, their anatomical descriptions of thoracic limb muscles are not detailed. König and Pérez (2022) only described them without the support of figures. Hudson and Hamilton (2010) provided graphical support with drawings and summarized descriptions. Gil Cano et al. (2023) provided only graphical support through photos of their dissections. Reighard and Jennings (1901) have anatomical descriptions with more detail and are complemented by muscle maps. Interestingly, several studies have reviewed the anatomical arrangement of the thoracic limb muscles in several wild species of felids, such as *Puma concolor* (Concha et al., 2004; da Costa da Silva et al., 2025), *Leopardus pardalis* (da Costa da Silva et al., 2025; Julik et al., 2012; Sánchez et al., 2019), *Leopardus geoffroyi* (Cardozo et al., 2025), *Leopardus wiedii* (da Costa da Silva et al., 2025), *Lynx lynx* (Ari, 2024; Viranta et al., 2016), *Atelocynus jubatus* (Hudson et al., 2011; Ross, 1883), *Panthera leo* (Barone, 1967; Vargas et al., 2017), *Panthera onca* (Sánchez et al., 2019), *Panthera uncia* (Smith et al., 2021), and *Panthera tigris* (Dunn et al., 2022). However, several of those studies compared their results with the anatomical descriptions of the former book “Anatomy of the Cat” by Reighard and Jennings (1901) (Cardozo et al., 2025; Concha et al., 2004; da Costa da Silva et al., 2025; Dunn et al., 2022; Sánchez et al., 2019; Smith et al., 2021), which is also used as an anatomical basis for textbooks on comparative anatomy of vertebrates (De Iuliis & Pulerà, 2019; Sebastiani & Fishbeck, 2005). Moreover, Reighard and Jennings (1901) used former terminology because of the publication time of their book (before the publication of the first edition of the *Nomina Anatomica Veterinaria* [NAV] in 1978). Thus, several recent myological studies of the thoracic limb in felids have used former terms or confused muscles. Some of those studies included muscle maps because they are useful for muscle reconstructions in fossils (Cardozo et al., 2025; Dunn et al., 2022; Julik et al., 2012; Smith et al., 2021) and for the diagnosis of damaged structures around the fractured bones and for surgical planning and approaches in veterinary medicine of felids.

Evolutionary muscular studies in felids have inferred the homology and derivation of the neck and head muscles in the serval (*Leptailurus serval*) and *Pa. tigris* (Diogo et al., 2012) and extrinsic thoracic limb muscles of *F. catus* (Vélez‐García et al., 2024). These studies were based on Diogo and Abdala (2010) and Diogo et al. (2018), who stated that to establish homologies and infer the derivation of the muscles from gross dissections, not only the topology should be used but also other information, such as the available literature, orientation of the muscle fibers, innervation, function, absence or presence of muscles, and embryological development. The latter should be based on the embryonic muscle splitting patterns observed in the most recent embryological studies performed in representative species of different clades (Bishop & Pierce, 2024; Smith‐Paredes et al., 2022). Thereby, although there is no study of the embryological development of the thoracic limb muscles in any representative carnivoran, evolutionary studies based on the close anatomical relationship, innervation, and embryological development in other representative mammals inferred the homology and derivations of extrinsic thoracic limb muscles in carnivorans of the families Felidae and Procyonidae (Diogo et al., 2012; Vélez‐García et al., 2024; Vélez‐García & Miglino, 2023). Therefore, this study aimed to describe in detail the thoracic limb muscles and the brachial plexus of *F. catus* to reconstruct muscle maps, report anatomical variants, establish homologies, and infer muscle derivations in felids. In this chapter, we report the attachments (origin and insertion), innervation, and arterial supply of the shoulder and brachium muscles and the origin and distribution of the brachial plexus (*Plexus brachialis*) of *F. catus* in these regions.

## 2 MATERIALS AND METHODS

### 2.1 Bioethical license

This research was approved by the Bioethics Committee of the Faculty of Veterinary Medicine and Animal Science of the Universidade de São Paulo (CEUAx agreement number 3928240820). The animals were not sacrificed for the purpose of this study.

### 2.2 Specimens and location of the research

Nineteen thoracic limbs from 10 *F. catus* specimens were dissected. These limbs were used in the classes of Applied Anatomy of the program of Veterinary Medicine of the Universidade de São Paulo. Each specimen was identified in order of dissection (FcS1–FcS10), and their right and left thoracic limbs were identified as RTL and LTL, respectively (FcS1‐RTL/LTL–FcS10‐RTL/LTL). Some of these specimens were used in previous investigations of their extrinsic thoracic limb muscles (Vélez‐García et al., 2024). However, the order of dissection was different in this study; thus, the identification number of the specimens was different (Table 1). The specimens were dissected at the Veterinary Anatomy Laboratory of the Universidade de São Paulo (São Paulo, Brazil).

**TABLE 1:** Identification of *Felis catus* specimens and dissected thoracic limbs.

Species	ID	Sex	Age	Limb	Studied anatomical parts
*Felis catus*	FcS1	F	A	RTL	IShMm, BMm, AnMm and BP
				LTL	IShMm, BMm and BP
	FcS2	F	J	RTL	IShMm, BMm, AnMm and BP
				LTL	IShMm, BMm and BP
	FcS3	F	J	RTL	IShMm, BMm and BP
				LTL	IShMm, BMm, AnMm and BP
	FcS4	M	A	RTL	IShMm, BMm and BP
				LTL	IShMm, BMm, AnMm and BP
	FcS5	M	A	RTL	IShMm, BMm, AnMm and BP
				LTL	IShMm, BMm and BP
	FcS6	M	A	RTL	IShMm, BMm and AnMm
				LTL	IShMm, BMm and BP
	FcS7	F	sA	LTL	IShMm, BMm, AnMm and BP
	FcS8	M	sA	RTL	IShMm, BMm, AnMm and BP
				LTL	IShMm, BMm, AnMm and BP
	FcS9	M	A	RTL	BP
				LTL	BP
	FcS10	F	A	LTL	IShMm, BMm and AnMm
				RTL	IShMm, BMm and AnMm

Abbreviations: A, adult; AnMm, Antebrachial muscles; BMm, Brachial muscles; BP, Brachial plexus; F, female; ID, specimen identification; IShMm, Intrinsic shoulder muscles J, juvenile; LTL, left thoracic limb; M, male; RTL, right thoracic limb; sA, sub‐adult.

### 2.3 Fixation and vascular repletion of specimens

The specimens were conserved frozen at the Laboratory of Veterinary Anatomy of the Universidade de São Paulo (USP). Seven *F. catus* specimens (FcS1‐FcS6 and FcS9) were completely thawed, fixed, and preserved according to the protocol for fixation and conservation of cadavers of the Veterinary Anatomy Laboratory of the Universidade de São Paulo (USP). These specimens were fixed with 10% formaldehyde via the common carotid artery and subsequently immersed and preserved in closed containers with 10% formaldehyde. After 48 h, the specimens were submerged and preserved in 30% hypersaturated saline solution. Two specimens (FcS7 and FcS8) were completely thawed and repleted with natural latex tinctured with red vinyl via the femoral artery. Afterward, they were fixed with 10% formaldehyde via the femoral vein and subjected to subcutaneous and intramuscular infiltrations. They were immersed and preserved in closed containers with 5% formaldehyde. One specimen (FcS10) was completely thawed and dissected without being fixed.

### 2.4 Dissection and documentation

First, the skin was removed from the dorsal regions of the neck and trunk to the distal extremity of the thoracic limbs. Second, the fascia around the muscles, nerves, and vessels was removed. Third, the pectoral muscles were detached from the sternum, and the ventral parts of the thorax (sternum, costal cartilages, viscera, and vessels—except the subclavian arteries) and neck (muscles and viscera) were removed until the emergence of the ventral branches of the cervical and thoracic spinal nerves was observed. Fourth, the origin of the brachial plexus and its nerves was determined based on the contribution of the ventral branches of the cervical and thoracic spinal nerves. The distribution of the brachial plexus nerves was observed during the dissection of the muscles. Fifth, the types of attachments (origin and insertion) of each muscle were classified as fleshy when the muscle fibers were observed to attach directly or tendinous and aponeurotic when tendons or plane tendons were attached directly, respectively. The muscle maps were reconstructed in the left thoracic limb bones of the first dissected specimen (FcS1‐LTL), representing the distribution area of the muscle attachments. These muscle maps were complemented with insertions of the extrinsic thoracic limb muscles based on previous descriptions (Vélez‐García et al., 2024) and the new specimens of the present study (FcS7, FcS8, and FcS10). In addition, the attachments of the elbow ligaments and the zones where the nerves make contact with the bone were also represented in the muscle maps. The thoracic limb bones were digitized using a three‐dimensional scanning application (Polycam 2.0.2) with a smartphone (Motorola moto g83 5G) and the attachments were colored on the 3D bone meshes using Blender 4.4.3 (Blender, 2025). The architecture of the muscles was defined based on their superficial shape and the fiber direction in longitudinal sections performed at the muscle belly. The anatomical descriptions were based on the NAV (International Committee on Veterinary Gross Anatomical Nomenclature, 2017). However, we used the term *m. anconeus lateralis* (AL) to refer to the *m. anconeus* of the NAV based on Barone (2020) since *F. catus* has a *m. anconeus medialis* (AM). The term *n. cleidobrachialis* was also used in this study based on our findings for the most cranial nerve of the brachial plexus. The names and abbreviations of the muscles, nerves, and arteries are summarized in Table 2. The origins of three brachial plexuses could not be studied due to damage during previous dissections. Thereby, the total sample for brachial plexus origin was only 16 limbs, although the distribution of their nerves could be observed in all limbs (Table 1). Photographs of the dissections were taken with a Canon T5i 18 MP camera equipped with a Canon macro lens of 50 mm. The writing was reviewed using two artificial intelligence‐based tools (RUBRIQ and Copilot).

**TABLE 2:** Abbreviations of muscles, nerves, and arteries.

Abbreviation	Name (Latin name)
*Muscles*	
AH	m. articularis humeri
AL	m. anconeus lateralis^1^
AM	m. anconeus medialis^1^
B	m. brachialis
BB	m. biceps brachii
CB	m. coracobrachialis
CBl	m. coracobrachialis longus
ClB	m. cleidobrachialis
CT	m. cutaneus trunci
D	m. deltoideus
Da	m. deltoideus pars acromialis
Ds	m. deltoideus pars scapularis
IS	m. infraspinatus
LD	m. latissimus dorsi
LDl	Lateral belly of LD (*m. latissimus dorsi pars lateralis*)^2^
LDm	Medial belly of LD (*m. latissimus dorsi pars medialis*)^2^
OT	m. omotransversarius
PAb	m. pectoralis abdominalis^2^
PAn	m. pectoralis antebrachialis^2^
PD	m. pectoralis descendens
PP	m. pectoralis profundus
PPCr	m. pectoralis profundus pars cranialis^2^
PPCd	m. pectoralis profundus pars caudalis^2^
PTr	m. pectoralis transversus
RhCa	m. rhomboideus capitis
RhCeT	m. rhomboideus cervicis et m. rhomboideus thoracis
Sb	m. subscapularis
SS	m. suprascapularis
SVC	m. serratus ventralis cervicis
SVT	m. serratus ventralis thoracis
TB	m. triceps brachii
TBa	m. triceps brachii caput accessorium
TBLa	m. triceps brachii caput laterale
TBLo	m. triceps brachii caput longum
TBm	m. triceps brachii caput mediale
Tc	m. trapezius pars cervicalis
Tt	m. trapezius pars thoracica
TFA	m. tensor fasciae antebrachii
TMaj	m. teres major
TMin	m. teres minor
*Nerves*	
AxN	Axillary nerve (*N. axillaris*)
AxN^Da^	Branch of AxN to Da
AxN^Ds^	Branch of AxN to Ds
AxN^ClB^	Branch of AxN to ClB
AxN^CrLBC^	Cranial lateral brachial cutaneous nerve (*N. cutaneus brachii lateralis cranialis*)
AxN^TMin^	Branch of AxN to TMin
CACdN	Caudal antebrachial cutaneous nerve (*N. cutaneus antebrachii caudalis*)
ClBN	Cleidobrachial nerve (*N. cleidobrachialis*)^3^
McN	Musculocutaneus nerve (*N. musculocutaneus*)
McN^CB^	Branch to m. coracobrachialis
McN^CBl^	Branch to m. coracobrachialis longus
McN^cbMN^	Communicating branch of McN with MN
McN^dmb^	Distal muscular branch (*ramus muscularis distalis*)
McN^EJC^	Branch of McN to elbow joint capsule
McN^MAC^	Medial antebrachial cutaneous nerve (*n. cutaneus antebrachii medialis*)
McN^pmb^	Proximal muscular branch (*ramus muscularis proximalis*)
MN	Median nerve (*N. medianus*)
RN	Radial nerve (N. radialis)
RNdb	Deep branch of RN (*Ramus profundus*)
RNsb	Superficial branch of RN (*Ramus superficialis*)
RN^TFA^	Branch of RN to TFA
RN^TBLo^	Branches of RN to TBLo
RN^TBLo‐TFA^	Common branch of RN to TFA and TBLo
SbNn	Subscapular nerves (*Nn. subscapulares*)
SbCrN	Cranial subscapular nerve (*N. subscapularis cranialis*)^4^
SbCdN	Caudal subscapular nerve (*N. subscapularis caudalis*)^4^
SSN	Suprascapular nerve (*N. suprascapularis*)
SSN^SS^	Branch of SSN to SS
SSN^IS^	Branch of SSN to IS
SSN^ShJC^	Branch of SSN to shoulder joint capsule
TDN	Thoracodorsal nerve (*N. thoracodorsalis*)
TLaN	Lateral thoracic nerve (*N. thoracicus lateralis*)
TLoN	Long thoracic nerve (*N. thoracicus longus*)
UN	Ulnar nerve (*N. ulnaris*)
UN^AM^	Branches of UN to AM
*Arteries*	
AA	Axillary artery (*A. axillaris*)
BA	Brachial artery (*A. brachialis*)
CdCHA	Caudal circumflex humeral artery (*A. circumflexa humeri caudalis*)
CRA	Collateral radial artery (*A. collateralis radialis*)
CrCHA	Cranial circumflex humeral artery (*A. circumflexa humeri cranialis*)
CSA	Cervical superficial artery (*A. cervicalis superficialis*)
CScA	Circumflex scapular artery (*A. circumflexa scapulae*)
CUA	Collateral ulnar artery (*A. collateralis ulnaris*)
DBA	Deep brachial artery (*A. profunda brachii*)
SbA	Subscapular artery (*A. subscapularis*)
SBA	Superficial brachial artery (*A. brachialis superficialis*)
SSA	Suprascapular artery (*A. suprascapularis*)
TCA	Transverse cubital artery (*A. transversa cubiti*)
TDA	Thoracodorsal artery (*A. thoracodorsalis*)

^1^ Terms based on Barone (2020).

^2^ Terms based on Vélez‐García et al. (2024).

^3^ Suggested term based on the present study. Other studies in felids named it *N. supraclavicularis* (Pérez & König, 2022) or *N. brachiocephalicus* (Souza‐Junior et al., 2022).

^4^ Terms adapted from the term “*Nn. subscapulares*” of NAV (International Committee on Veterinary Gross Anatomical Nomenclature, 2017).

### 2.5 Comparative anatomy for evolutionary derivation inferences

The findings of the thoracic limb muscles of *F. catus* were compared with those of other available studies in this species (Barone, 2020; Hudson & Hamilton, 2010; Pérez & König, 2022; Reighard & Jennings, 1901; Sebastiani & Fishbeck, 2005) and wild felids (Barone, 1967; Cardozo et al., 2025; Concha et al., 2004; da Costa da Silva et al., 2025; Dunn et al., 2022; Julik et al., 2012; Smith et al., 2021; Vargas et al., 2017). The homology and evolutionary derivation inferences for the muscles were based primarily on those outlined for vertebrates, such as topology, anatomical variants, innervation, presence and absence of muscles (Diogo et al., 2018), and the most recent study on embryonic muscle splitting patterns of amniotes (Smith‐Paredes et al., 2022).

## 3 RESULTS

### 3.1 Lateral scapular and shoulder muscles

#### 3.1.1 M. deltoideus (D)

The D has two parts: the pars acromialis (Da) and the pars scapularis (Ds) (Figure 1). The Da is unipennate and originates via tendinous and fleshy fibers from the ventral margin of the hamatus process of the acromion. The tendinous fibers originate in the superficial aspect, whereas the origin via fleshy fibers is deep. The Ds is fusiform and originates via an aponeurosis from the scapular spine and fleshy fibers from the superficial fascia of the m. infraspinatus (IS) and the distal extreme of the scapular spine (just proximal to the suprahamatus process) (Figure 2, Supplementary material 1). Its fleshy fibers are directed craniodistally to unite with Da at a few distances distal to the suprahamatus process. The cranial fleshy fibers of Da insert directly onto the cranial half of the lateral surface between the crest of greater tubercle and deltoid tuberosity (Figure 3, Supplementary material 2). The Da forms medial and caudal tendon fibers with Ds to insert onto the caudal half of the lateral surface between the crest of greater tubercle and deltoid tuberosity, brachial fascia over the m. brachialis (B), deltoid tuberosity, and proximal extreme of the humeral crest (Figure 3, Supplementary material 2). The axillary nerve (*N. axillaris*—AxN) and the caudal circumflex humeral artery (*A. circumflexa humeri caudalis*—CdCHA) supply both parts (Figure 1). One anatomical variant was found in the origin of one limb (FcS1‐LTL), in which the Da extended until the ventral surface of the suprahamatus process (Table 3).

**FIGURE 1:**
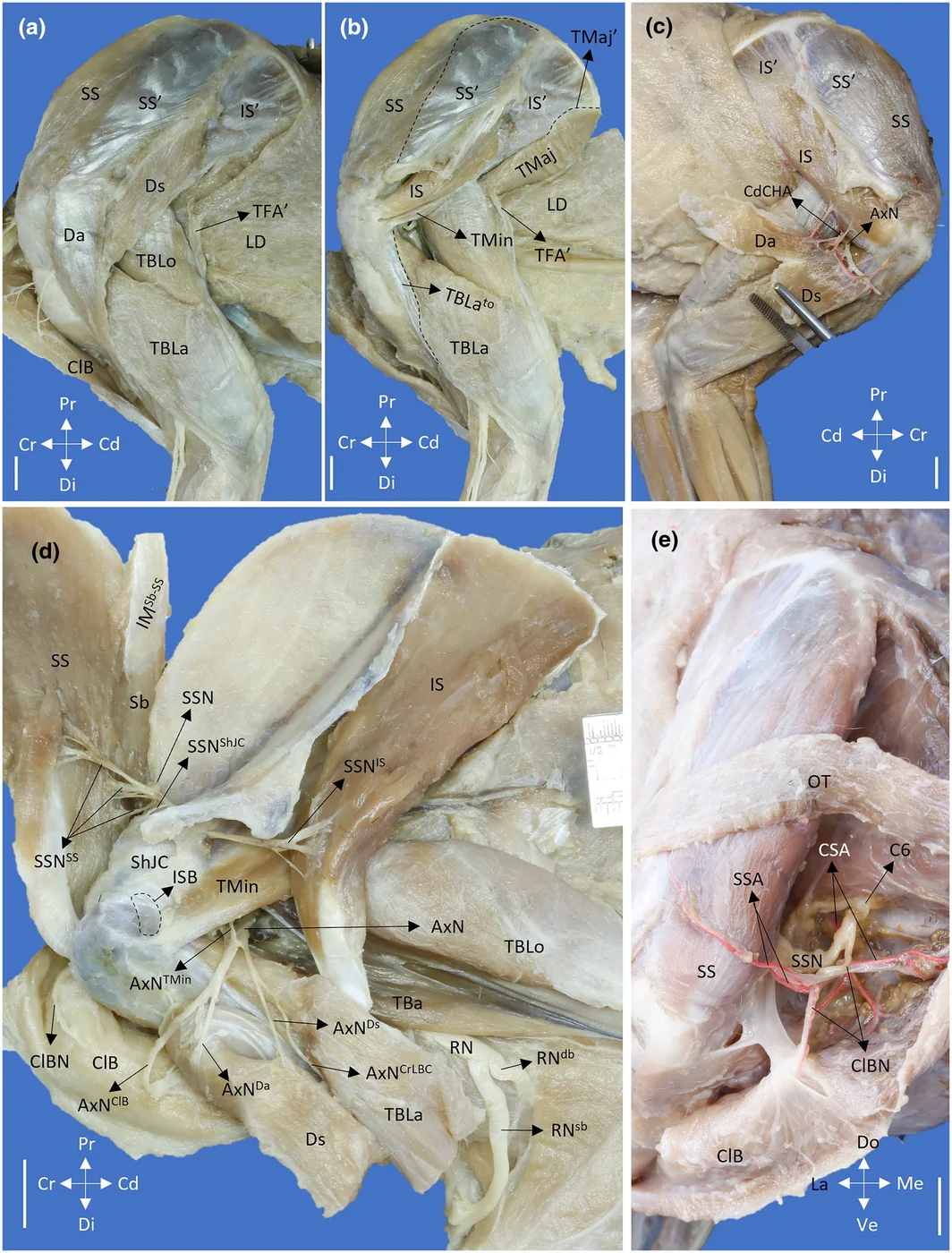
Intrinsic shoulder muscles of domestic cat (*Felis catus*). (a) Left superficial lateral view; (b) left deep superficial view after removal of the m. deltoideus; (c) right lateral view after distal retraction of the m. deltoideus to expose the nerves and vessels; (d) left deep lateral view highlighting the distribution of suprascapular and axillary nerves; (e) cranial view of the right prescapular region after cranial retraction of the m. cleidobrachialis to expose the nerves and vessels. Cd, caudal; CdCHA, caudal circumflex humeral artery; Cr, cranial; CSA, cervical superficial artery; Di, distal; Do, dorsal; IM^Sb‐SS^, intermuscular septum of Sb and SS; IS', superficial fascia of the IS; ISB, infraspinatus bursa; La, lateral; Me, medial; Pr, proximal; ShJC, shoulder joint capsule; SSA, suprascapular artery; SS', superficial aponeurosis of the SS; TBLa^to^, aponeurosis of origin of TBLa; TFA’, variant origin of the TFA from the TMaj; TMaj’, origin of the TMaj from the IS’; Ve, ventral. White bars: 10 mm.

**FIGURE 2:**
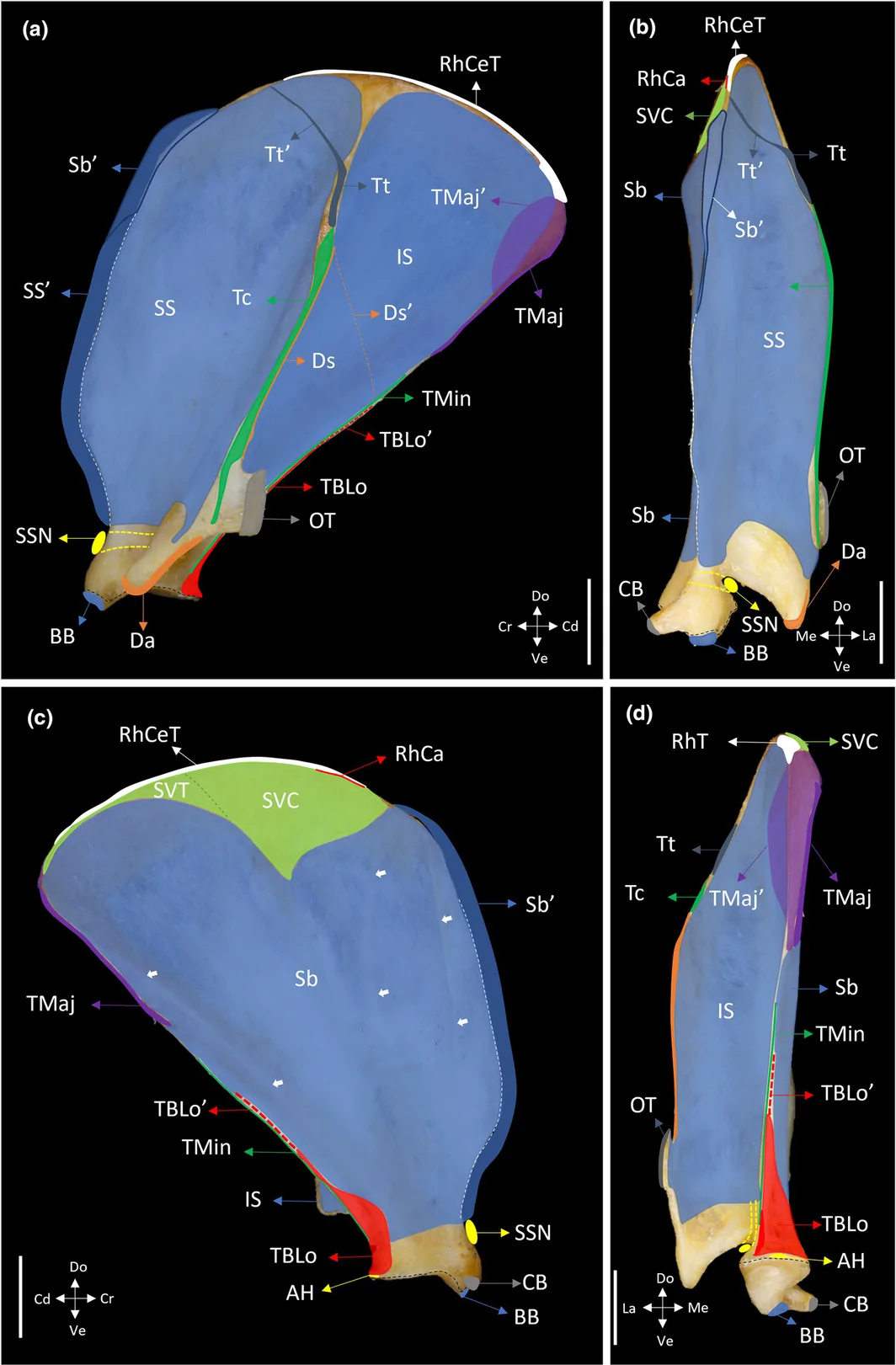
Muscle maps (origin and insertion) of the left scapula of domestic cat (*Felis catus*). (a) Lateral view; (b) cranial view; (c) medial view; (d) caudal view. Wide white arrows indicate the subscapular lines. Cd, caudal; Cr, cranial; Do, dorsal; Ds′, origin of Ds from the IS; La, lateral; Me, medial; Sb′, cranial projection of the Sb (intermuscular septum with SS); SS', cranial projection of the SS (intermuscular septum with Sb); TBLo’, oblique tendinous bands of the tendon of origin of TBLo; TMaj’, origin of TMaj from the IS; Tt’, origin of Tt from the superficial aponeurosis of the SS; Dark dashed lines, insertion of the shoulder joint capsule; Ve, ventral. Yellow dashed lines, contact zone of SSN with the scapula. The green dashed line represents the estimated separation of the SVT and SVC. The white dashed lines indicate the location of the intermuscular septum between Sb and SS. The white wide arrows indicate the lines of the subscapular fossa. White bars: 10 mm.

**FIGURE 3:**
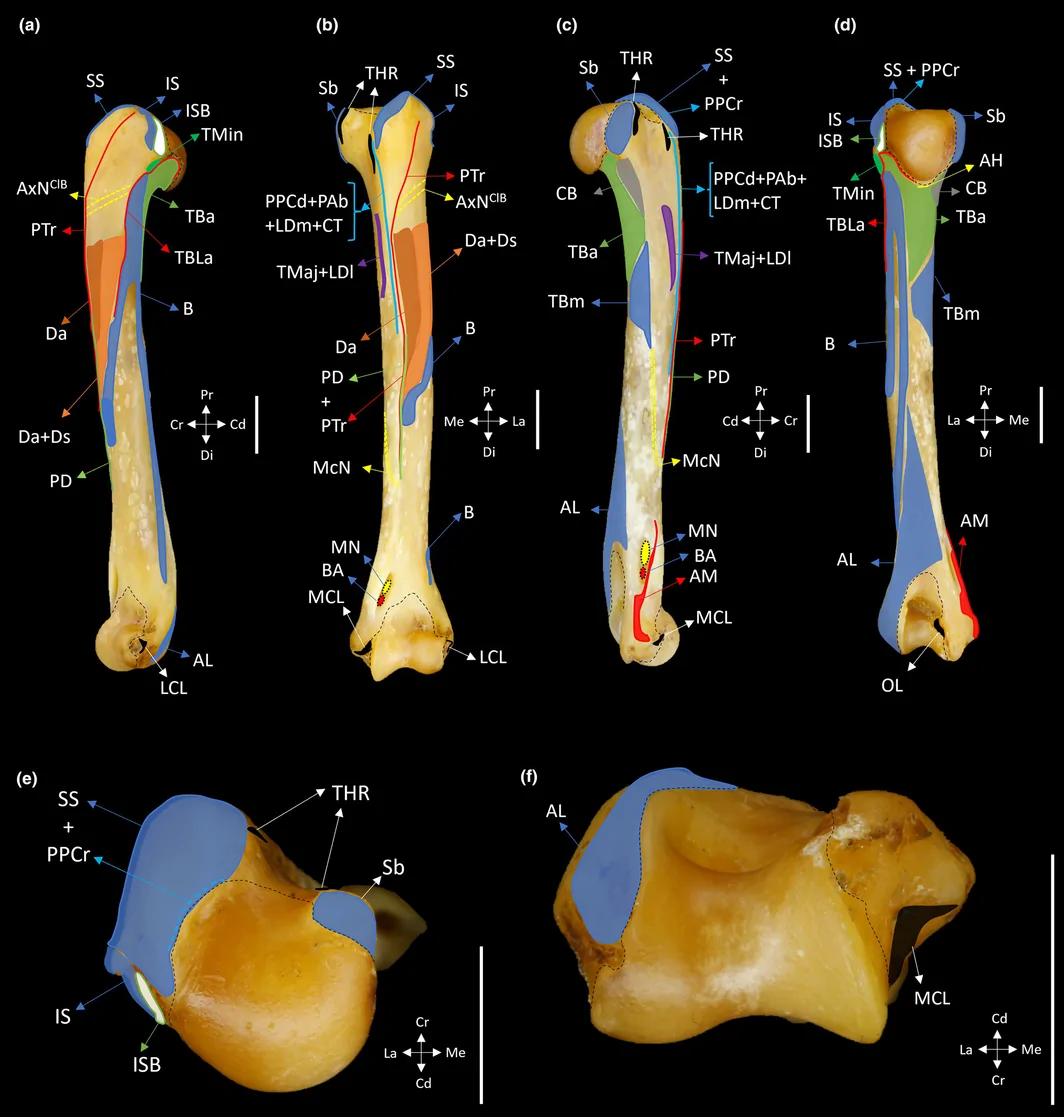
Muscle maps (origin and insertion) of the left humerus of domestic cat (*Felis catus*). (a) Lateral view; (b) cranial view; (c) medial view; (d) caudal view; (e) proximal view; (f) distal view. BA, brachial artery; Cd, caudal; Cr, cranial; Di; distal; ISB, synovial bursa of the IS; La, lateral; LCL, lateral collateral ligament of the elbow; MCL, medial collateral ligament of the elbow; Me, medial; OL, olecranon ligament; Pr, proximal; THR, transverse humeral retinaculum. +, common insertion of muscles or ligaments. Dark dashed lines, insertion of the shoulder (proximal) and elbow (distal) joint capsules. Yellow dashed lines, contact zones of AxN^ClB^ and McN with the humerus. White bars: 10 mm.

**TABLE 3:** Main anatomical variants of the shoulder and brachium muscles of domestic cat (*Felis catus*).

Muscle	Variant	Limb	Prevalence (%)
AH	Presence of AH and insertion onto the TBa	FcS8‐RTL	5.26 (1/19)
AL	Insertion covering the proximal extreme of the m. extensor digiti I et II (EDI‐II)	FcS1 (bilaterally) and FcS6 (bilaterally)	21.05 (4/19)
	Insertion causing a proximal bifurcation of EDI‐II	FcS3 (bilaterally) and FcS4 (bilaterally)	21.05 (4/19)
	The insertion reached the level to the lateral coronoid process of ulna	FcS6 (bilaterally)	10.53 (2/19)
AM	Displaced distally by the m. coracobrachialis longus	FcS3‐RTL	5.26 (1/19)
	Additional insertion via fleshy fibers proximal to its tendinous insertion	FcS1 (bilaterally), FcS2 (bilaterally), FcS3‐LTL, and FcS5‐RTL	31.58 (6/19)
	Additional arterial supply by a branch of the brachial artery	FcS7‐LTL	–
B	Origin of m. brachioradialis fused to B	FcS10 (bilaterally)	10.53 (2/19)
	Additional innervation by the deep branch of the radial nerve	FcS4‐RTL	5.26 (1/19)
BB	Presence of a vestigial caput breve	FcS2 (bilaterally)	10.53 (2/19)
	Additional aponeurotic fascicle inserted onto the insertion tendon of m. brachialis	FcS6‐RTL and FcS10 (bilaterally)	15.78 (3/19)
	Additional arterial supply by a variant bicipital artery	FcS2‐RTL and FcS5‐RTL	–
CB	Two bellies through which the musculocutaneus nerve passes	FcS8‐RTL	5.26 (1/19)
	Presence of m. coracobrachialis longus (CBl)	FcS3‐RTL, FcS6 (bilaterally), FcS7 (bilaterally), and FcS10‐RTL	31.58 (6/19)
	Presence of a small CBl inserted onto the medial deep brachial fascia	FcS6 bilaterally, FcS7 bilaterally and FcS10‐RTL	26.31 (5/19)
	Presence of a large CBl inserted onto the supracondylar foramen	FcS3‐RTL	5.26 (1/19)
	Arterial supply via a branch of subscapular artery	FcS7‐LTL	–
Da	Extended origin until the suprahamatus process	FcS1‐LTL	5.26 (1/19)
Sb	Six bellies because the fourth belly divided into two bellies	FcS3‐LTL	5.26 (1/19)
	The second belly formed an accessory belly, which formed another subscapular line in the scapula	FcS4 (bilaterally)	10.53 (2/19)
	The first and second bellies formed another tendon that inserted superficially to the main tendon onto the lesser tubercle, and caudally fused to this latter tendon	FcS5 (bilaterally)	10.53 (2/19)
	Additional arterial supply by a branch of caudal circumflex humeral artery	FcS7‐LTL	5.26 (1/19)
SS	Tendon of insertion overlapped the tendon of IS proximally	FcS6‐RTL	5.26 (1/19)
TBa	Intermixed fleshy fibers with TBm	FcS4‐LTL	5.26 (1/19)
TBLa	Additional arterial supply by deep brachial and collateral ulnar arteries	FcS8‐RTL	–
TBLo	Tendon of origin without oblique tendon fascicles	FcS6‐RTL	5.26 (1/19)
	Tendinous band originated from the fascia over IS	FcS5‐LTL	5.26 (1/19)
TBm	Tendinous fascicle originated from the intertubercular groove	FcS4‐LTL	5.26 (1/19)
TFA	A small fascicle originated from the the TMaj	FcS1‐LTL	5.26 (1/19)
	Muscle divided into two parts: cranial and caudal	FcS5‐LTL	5.26 (1/19)
	Muscle divided into two parts: superficial and deep	FcS8‐LTL	5.26 (1/19)
	No insertion onto the tendon of insertion of the TBLo	FcS5‐LTL	5.26 (1/19)
TMin	Origin from the ventral half of the caudal margin of the scapula	FcS2‐RTL, FcS1 (bilaterally) and FcS8 (bilaterally)	26.31 (5/19)
	Additional insertion fleshy fibers in the deep aspect	FcS4‐LTL and FcS6‐RTL	10.53 (2/19)

*Note*: Absence of prevalence due to the arterial supply was studied in a few specimens.

Most authors reported that in *Felis catus*, the unique origins of Ds and Da were the scapular spine and acromion, respectively (Barone, 2020; De Iuliis & Pulerà, 2019; Hudson & Hamilton, 2010; Sebastiani & Fishbeck, 2005). Pérez and König (2022) described the origin of Ds only from the aponeurosis of the IS. Reighard and Jennings (1901) reported the origin from the scapular spine, the fascia over the IS, and the connection with the aponeurosis of M. trapezius. We also found this last connection, which made it difficult to separate the insertion aponeurosis of the m. trapezius pars thoracica (Tc) from the aponeurosis origin of the Ds and fascia over the IS. All three aponeuroses seem to fuse, which may explain why several authors have distinct interpretations of the attachments of these muscles in this zone (infraspinous region). Reighard and Jennings (1901) reported that Da can reach the suprahamatus process, a characteristic that we found in only one limb. Controversially, they represented that arrangement as the typical pattern in their muscle maps (Reighard & Jennings, 1901), similar to other authors (Hudson & Hamilton, 2010). This also occurred with Dunn et al. (2022), who did not describe that origin in *Panthera tigris* but represented it in the muscle maps of the scapula. This origin is present in *Panthera leo* (Barone, 1967; Vargas et al., 2017), *Panthera uncia* (Smith et al., 2021), *Leopardus wiedii, Leopardus pardalis*, and *Puma concolor* (da Costa da Silva et al., 2025). However, it can be missing in the last two species (Concha et al., 2004; Julik et al., 2012). The Ds can also reach the suprahamatus process in *Pa. leo* (Barone, 1967; Vargas et al., 2017), whereas in *Le. pardalis* and *Pa. tigris*, it is the only bony origin of Ds because the other origin is restricted to the fascia over the IS (Dunn et al., 2022; Julik et al., 2012). In *Acinonyx jubatus*, Da is represented only by the suprahamatus process (Hudson et al., 2011), which differs from one study in the same species (Ross, 1883). No authors have reported the additional origins via fleshy fibers of Da and Ds in *F. catus* and other species, but in *Leopardus geoffroyi* (Cardozo et al., 2025). Interestingly, in this latter species, the Da only originates via fleshy fibers and fuses with Ds at their origins, and both reach the suprahamatus process (Cardozo et al., 2025). The Da can also originate from the fascia over the SS in *Le. pardalis* (da da Costa da Silva et al., 2025; Julik et al., 2012), *Pu. concolor*, and *Le. wiedii* (da da Costa da Silva et al., 2025). Similar to our findings, the authors reported partial insertions via fleshy and tendon fibers in *F. catus* (Pérez & König, 2022), *Le. geoffroyi* (Cardozo et al., 2025), and *Pa. tigris* (Dunn et al., 2022). We always observed strong fascial connections of Ds and Da with B and caput laterale of m. triceps brachii (TBLa), respectively, which made it difficult to define the separation of these muscles in this zone. Therefore, this could be the reason why the interpretations of the attachments of these muscles vary among authors. Among these cases, the Ds has been reported to be inserted onto TBLa in *F. catus* (Pérez & König, 2022), *Pa. leo* (Vargas et al., 2017), and *Le. pardalis* (Julik et al., 2012). In other cases, the authors reported that it fused partially with TBLa in one limb of the *Pa. uncia* (Smith et al., 2021) and both limbs of a *Pa. tigris* (Dunn et al., 2022). The fibers of Da can pass into B in *F. catus* (Reighard & Jennings, 1901) or insert onto B in *Le. pardalis* (Julik et al., 2012) and *Le. geoffroyi* (Cardozo et al., 2025).

#### 3.1.2 M. supraspinatus (SS)

The SS is a bipennate muscle that originates mainly via fleshy fibers from the supraspinous fossa, cranial surface of the scapular spine, and proximal third of the lateral aspect of the hamatus process (most surfaces of the hamatus process are free of the origin of the SS). The SS belly projects cranially to the cranial margin of the scapula, forming an intermuscular septum with the most cranial belly of m. subscapularis ‐Sb‐ (IM^SS‐Sb^), where it also originates (Figures 1 and 4). The origin of SS is also aponeurotic in the ventral two thirds of the cranial margin of the scapula and incisura scapulae (Figure 2, Supplementary material 1). It inserts via a short and strong tendon onto the proximal extreme of the greater tubercle of the humerus (Figure 3, Supplementary material 2). The tendon of the cranial part of the m. pectoralis profundus (PPCr) fuses medially with the SS tendon. The suprascapular nerve (*N. suprascapularis* ‐SSN‐) and the lateral and medial branches of the suprascapular artery (*A. suprascapularis* ‐SSA‐) supply it (Figure 1). The latter originates from the superficial cervical artery (*A. cervicalis superficialis* ‐SCA‐) (Figure 1(e)). In one limb (FcS6‐RTL), the SS presented a variant insertion in which its insertion tendon overlapped the tendon of IS proximally (Table 3).

**FIGURE 4:**
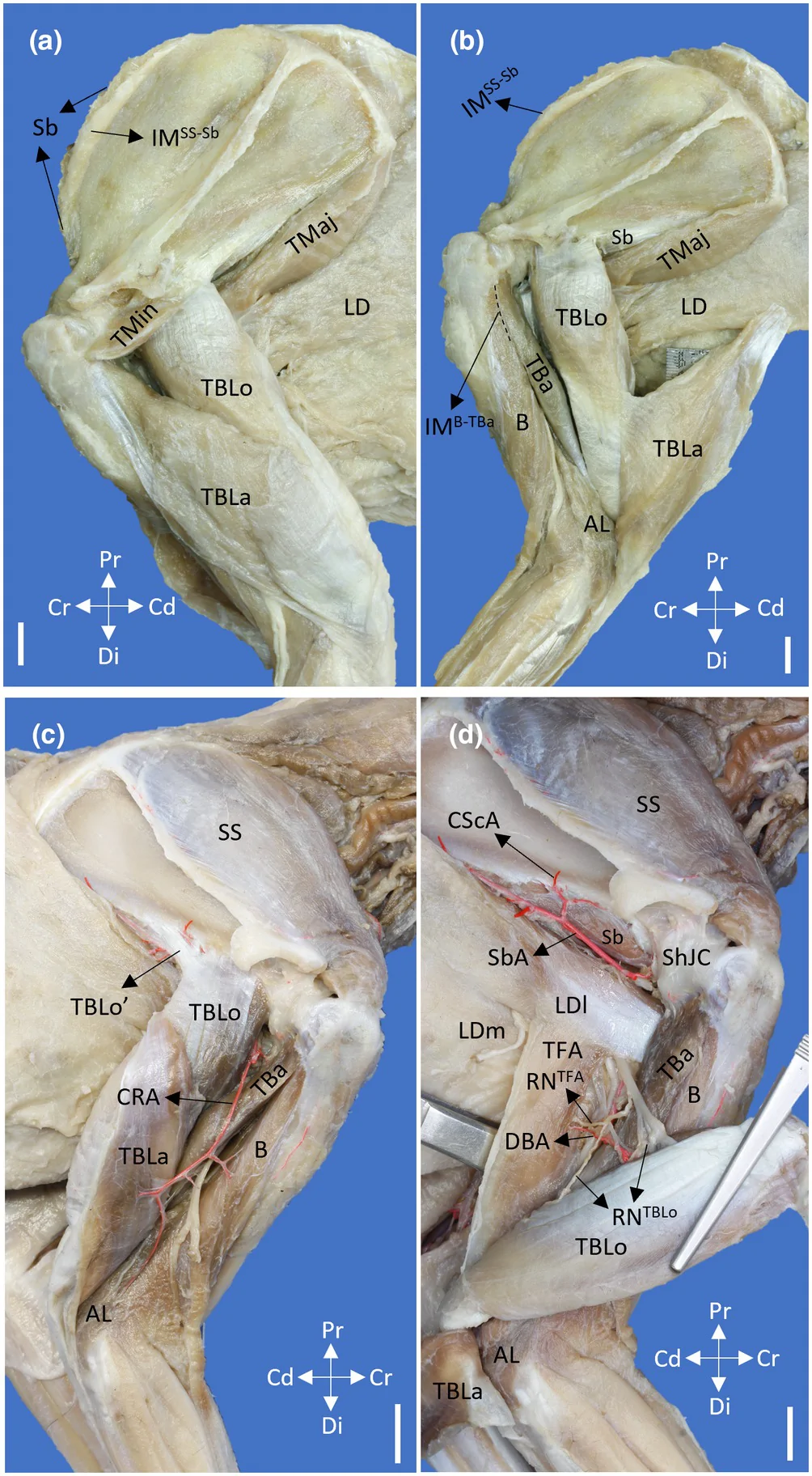
Lateral view of the shoulder and brachial muscles of domestic cat (*F. catus*). (a) Left lateral view after removing the SS and IS, (b) left lateral view after eliminating the TMin, (c) right lateral view after eliminating the IS and TMin and retracting the TBLa caudally, and (d) right lateral view after eliminating the IS and TMin and retracting the TBLo cranially. Cd, caudal; Cr, cranial; CRA, collateral radial artery; CScA, circumflex scapular artery; DBA, deep brachial artery; IM^SS‐Sb^, intramuscular septum of SS and Sb; Di; distal; IM^B‐TBa^, intramuscular septum of B and TBa; Pr, proximal; Sb, m. subscapularis; SbA, subscapular artery; ShJC, shoulder joint capsule; TBLo’, oblique tendinous bands of the tendon of origin of TBLo. White bars: 10 mm.

Most authors have reported only the origin of the SS from the supraspinous fossa and its insertion into the greater tubercle (Barone, 2020; De Iuliis & Pulerà, 2019; Hudson & Hamilton, 2010; Sebastiani & Fishbeck, 2005). Pérez and König (2022) reported that in *F. catus*, it also originates from the cranial margin of the scapula extending medially, and Hudson and Hamilton (2010) reported that it originates from the cranial extreme of the subscapular fossa (medial to the cranial margin of the scapula). However, we found a clear intermuscular septum in the cranial margin that separates the SS and Sb, and from which both muscles originate. Even Reighard and Jennings (1901) reported the origin between both SS and Sb and the origin from the fascia that covers the SS. We corroborated the presence of this strong fascia superficial to the SS, and through longitudinal sections, we also observed the fleshy fibers originating from it and the bipennate shape of the muscle. The cranial projection of the SS beyond the cranial margin of the scapula seems to be the typical pattern in felids based on the evidence of the descriptions and figures of all the authors. The SS also originates from the medial surface of the cranial margin of the scapula, reaching the cranial extreme of the subscapular fossa in *Lynx lynx* (Viranta et al., 2016), *A. jubatus* (Hudson et al., 2011), and *Le. geoffroyi* (Cardozo et al., 2025). In other cases, the SS fuses with the Sb in *Pa. uncia* (Smith et al., 2021), *Le. pardalis* (Julik et al., 2012), and *Pa. tigris* (Dunn et al., 2022) or shares fibers with Sb in *Le. geoffroyi* (Cardozo et al., 2025). In *Le. geoffroyi*, the SS rounds the cranial angle (Cardozo et al., 2025), which does not occur in *F. catus* because this zone is rounded by the Sb. Reighard and Jennings (1901) reported an additional insertion onto the shoulder joint capsule in *F. catus* similar to *Ly. lynx* (Viranta et al., 2016). In our dissections, the SS tendon always strongly adhered to the joint capsule, making it difficult to separate. In addition, it fuses with the tendon of the m. pectoralis profundus (PP), as occurs in the *Pa. uncia* (Smith et al., 2021), and similar to *Le. pardalis*, where it inserts onto this tendon (Julik et al., 2012). *Leopardus geoffroyi* differs from other felids since the SS inserts via fleshy fibers in the joint capsule and greater tubercle (Cardozo et al., 2025).

#### 3.1.3 M. infraspinatus (IS)

The IS is a pyramidal and bipennate muscle that originates via fleshy fibers from the infraspinous fossa, caudal surface of the scapular spine, medial surface of the suprahamatus process, and aponeurosis of origin of the m. teres minor (TMin) (Figures 1 and 2). It is covered by strong superficial fascia (IS’), from which its fleshy fibers originate. The aponeurosis of origin of Ds and the aponeurosis of insertion of m. trapezius pars thoracica (Tt) fuse with that strong fibrous tissue. In the two limbs (FcS8 bilaterally), the IS also originated proximally from the scapular cartilage. It inserts via a tendon onto the greater tubercle, just distal to the insertion of SS (*facies m. infraspinatus*) (Figure 3, Supplementary material 2). The synovial bursa is deep to its tendon (*Bursa subtendinea m. infraspinati*) and proximal to its insertion. The SSN, CdCHA, subscapular (*A. subscapularis* ‐SbA‐) and circumflex scapular (*A. circumflexa scapulae* ‐CScA‐) arteries supply it (Figure 1).

Most authors have reported only the origin of the IS from the infraspinous fossa and its insertion into the greater tubercle (Barone, 2020; De Iuliis & Pulerà, 2019; Hudson & Hamilton, 2010; Sebastiani & Fishbeck, 2005). Reighard and Jennings (1901) reported additional origins from TMaj and sometimes from other muscles (m. triceps brachii caput longum ‐TBLo‐, TMin, Ds, Sb, and Tt). Pérez and König (2022) reported additional origins from the caudal margin and aponeurosis of Ds. Among these additional origins described by these authors, the IS is covered by a strong fascia that surrounds the muscle belly and from which its fleshy fibers originate. As we described in the previous paragraph, this fascia seems to fuse with the aponeuroses of the Tt and Ds. Thus, when we cut these aponeuroses, the fleshy fibers of the IS originate from these, and the fleshy fibers of the Ds originate from that fascia of the IS. The origin via fleshy fibers from the aponeurosis of TMin was always present in our specimens, whereas the origins from Sb and TBLo were not. The origin of TMin is also present in *Le. geoffroyi*, whereas the origin of TBLo tendon varies in this species (Cardozo et al., 2025) and is present bilaterally in *Pa. tigris* (Dunn et al., 2022). Reighard and Jennings (1901) described another origin from a raphe with TMaj, which we did not observe but rather the continuation of the same strong fascia over the IS, from which the TMaj originates. In *Le. pardalis*, the IS originates from the origin of the TMaj and fuses partially with this muscle (Julik et al., 2012), similar to the *Pa. uncia* (Smith et al., 2021). Several authors have reported the synovial bursa of IS in *F. catus* (Barone, 2020; Pérez & König, 2022; Reighard & Jennings, 1901). The origin from the scapular cartilage as we observed in both limbs of one *F. catus* is not an anatomical variation since it is associated with the fact that part of cartilage has not yet ossified due to the juvenile age of the specimen, as was observed in other carnivorans (Pereira et al., 2016; Vélez García et al., 2024).

#### 3.1.4 M. teres minor (TMin)

The TMin is a fusiform muscle that originates via an aponeurosis from the ventral two thirds of the caudal margin of the scapula (Figure 1). There is a strong fibrous band that joins it to the origin tendon of TBLo. The TMin adheres medially to the shoulder joint capsule and inserts via a tendon onto the teres minor tuberosity of the humerus (Figure 3). The AxN and CdCHA supply it (Figure 1). As anatomical variations, we observed five limbs with an origin more dorsally extended, and in two forelimbs, the muscle also inserted via fleshy fibers deeply (Table 3).

Most authors have reported only the origin of TMin from the caudal margin of the scapula and its insertion into the greater tubercle (Barone, 2020; De Iuliis & Pulerà, 2019; Pérez & König, 2022; Reighard & Jennings, 1901; Sebastiani & Fishbeck, 2005). The origin from the infraglenoid tubercle, as stated by Liebich et al. (2020), was not found in our specimens. Hudson and Hamilton (2010) represented a small area adjacent to the caudal margin of the scapula in their muscle maps. Reighard and Jennings (1901) reported that TMin is often attached to the IS and TBLo in *F. catus*, and other authors reported the origin from the TBLo in *A. jubatus* (Ross, 1883), *Le. pardalis* (Julik et al., 2012), and *Pa. tigris* (Dunn et al., 2022). We observed a strong fascia that connects the aponeurosis of origin of TMin with the tendon of origin of TBLo, which could have been observed by other authors as an origin of TMin. In contrast with most species, the TMin of *Le. geoffroyi* originates mainly via fleshy fibers from the caudal margin and joint shoulder capsule (Cardozo et al., 2025). However, similar to our findings in *F. catus*, the aponeurotic origin of TMin is close to TBLo and does not originate from it (Cardozo et al., 2025). The insertion tendon of TMin fuses partially to the joint capsule in the *Pa. tigris* (Dunn et al., 2022) and, overall, in *Ly. lynx* (Viranta et al., 2016). The additional insertion via fleshy fibers, as occurred in our specimens, was also described in *Pa. tigris* (Dunn et al., 2022), *A. jubatus* (Ross, 1883), and one case of *Le. geoffroyi* (Cardozo et al., 2025). da Costa da Silva et al. (2025) did not report this muscle in their three dissected species. However, its presence has been confirmed in *Pu. concolor* (Concha et al., 2004) and *Le. pardalis* (Julik et al., 2012).

### 3.2 Medial muscles of the scapular and shoulder regions

#### 3.2.1 M. subscapularis (Sb)

The Sb is a multipennate muscle of five bellies (Sb^1‐‐5^) that originates mainly via fleshy fibers from the subscapular fossa and via tendinous fibers from three subscapular lines of the subscapular fossa (Figures 2, 4, and 5). The most cranial belly also originates via fleshy fibers from the intermuscular septum with the SS (IM^Sb‐SS^), the dorsal third of the cranial margin of the scapula, and the cranial, lateral, and dorsal surfaces of the cranial angle of the scapula (it reaches the zone of the dorsal margin just cranial to the origin of the m. serratus ventralis cervicis ‐SVC‐). The origin is aponeurotic in the ventral two thirds of the cranial margin of the scapula and scapular incisura. The intermediate tendon of the most cranial belly originates from the cranial subscapular line. The intermuscular septum between the third and fourth bellies originates from the second subscapular line, and the intermuscular septum between the fourth and fifth bellies originates from the third subscapular line. The origin was also via fleshy fibers from the ventral third of this latter line. The most caudal belly originates via fleshy fibers from the intermuscular septum with the m. teres major (TMaj) and via aponeurotic fibers from the caudal margin of the scapula between the origins of TMaj (dorsal) and TMin (ventral). The Sb inserts via a strong and short tendon onto the lesser tubercle. The cranial and caudal subscapular nerves (*Nn. subscapulares*: *N. subscapularis cranialis* ‐SbCrN‐ and *N. subscapularis caudalis* ‐SbCdN‐) and AxN innervate it, and the SSA, SbA, and CSA supply it. As anatomical variants, we observed the formation of accessory bellies, two tendons of insertion, and an additional arterial supply by CdCHA (Table 3).

**FIGURE 5:**
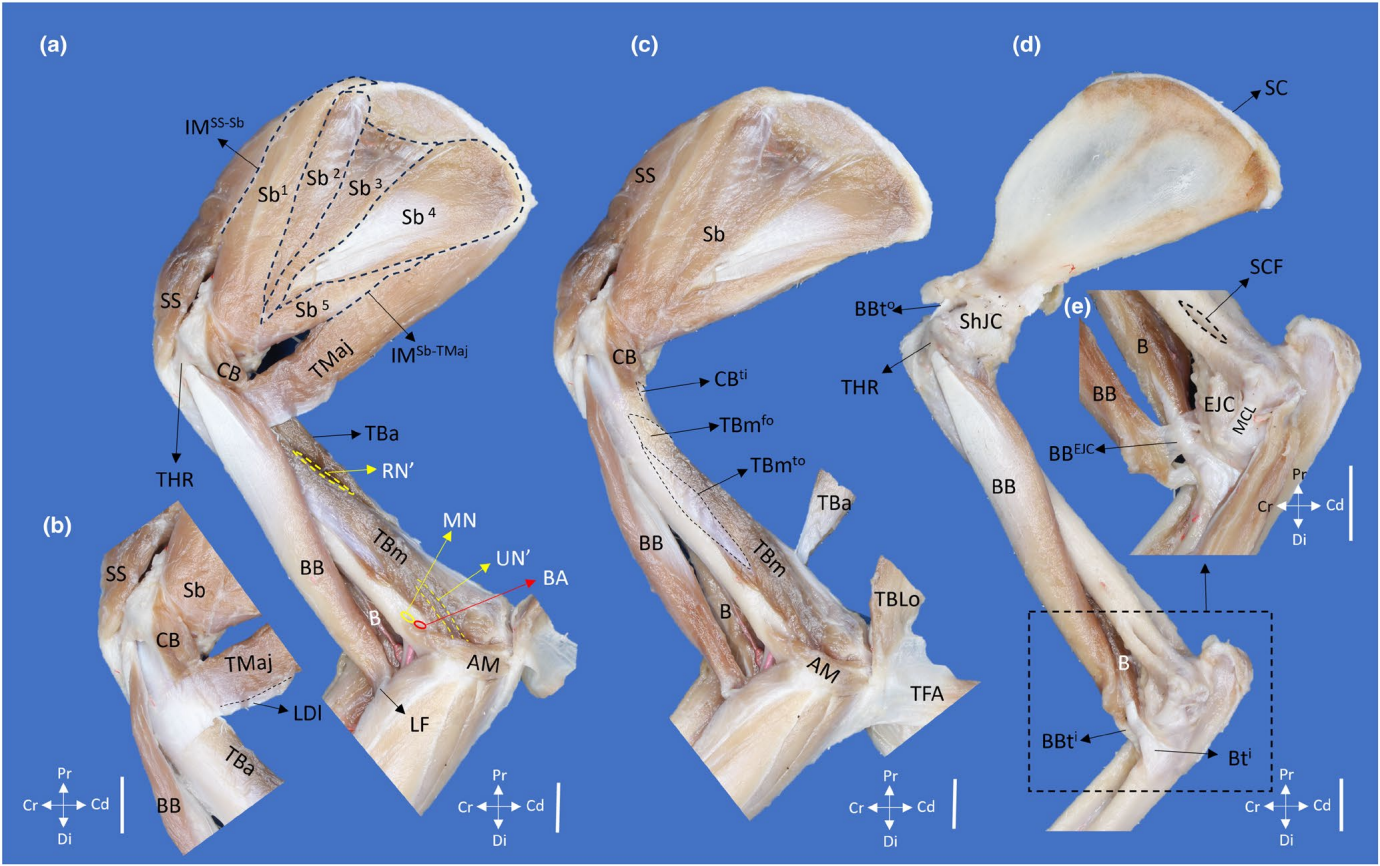
Medial views of the shoulder and brachial muscles of the right thoracic limb of domestic cat (*F. catus*). (a) Medial view after distal retraction of TBLo and TFA; (b) medial view after cranial retraction of BB to observe the common tendon of the insertion of the TMaj and LD; (c) medial view after removal of the TMaj to observe the insertion of the CB and the origin of the TBm; (d) medial view after removal of the TMaj; (e) medial view of the elbow after removal of the medial epicondylar muscles. BA, brachial artery; B^ti^, tendon of insertion of B; BB^to^, tendon of origin of BB; BB^ti^, tendon of insertion of BB; BB^EJC^, accessory aponeurotic fascicle of BB to the EJC; CB^ti^, tendinous fibers of insertion of CB; Cd, caudal; Cr, cranial; Di; distal; EJC, elbow joint capsule; IM^SS‐Sb^, intramuscular septum of SS and Sb; IM^Sb‐^™^aj^, intramuscular septum of Sb and TMaj; LF, lacertus fibrosus; Pr, proximal; RN’, intermuscular cleft to the passage of the radial nerve; Sb^1‐‐5^, bellies of the Sb; SC, scapular cartilage; SCF, supracondylar foramen; TBm^fo^, fleshy fibers of origin of TBm; TBm^to^, tendon of origin of TBm; THR, transverse humeral retinaculum; UN’, yellow dashed lines delimit the impression of the ulnar nerve in the TBm. White bars: 10 mm.

Most authors have reported only the origin of Sb from the subscapular fossa and its insertion onto the lesser tubercle (Barone, 2020; De Iuliis & Pulerà, 2019; Pérez & König, 2022; Reighard & Jennings, 1901; Sebastiani & Fishbeck, 2005). Reighard and Jennings (1901) reported the additional origin via tendon fibers from two or three lines of the subscapular fossa, which we corroborated. Pérez and König (2022) reported them as tendon bands from the aponeurosis that cover the Sb. Reighard and Jennings (1901) reported that Sb sometimes originates from the origin zone of TMaj, and when this occurs, the TMaj originates from Sb. However, we did not find that arrangement in our specimens; the most caudal belly of Sb always originated from the TMaj, and this latter conserved its origin zone from the scapula. Reighard and Jennings (1901) described the origin of the SS as a variant, whereas we found it in all specimens. The variant originating from the IS was not found. No authors have described the number of bellies in *F. catus*, although Barone (2020) reported that both domestic carnivorans (dog and cat) have four or five bellies. We found five bellies in most specimens and six as an anatomical variant. The Sb has five bellies in *Pa. leo* (Barone, 1967; Vargas et al., 2017), six or nine in *Le. pardalis* (Julik et al., 2012), six or seven in *Ly. lynx* (Viranta et al., 2016), and eight or nine in *Pa. uncia* (Smith et al., 2021). Several authors have reported fusions of Sb with SS and TMaj in *Le. pardalis* (Julik et al., 2012), *Pa. tigris* (Dunn et al., 2022), and *Le. geoffroyi* (Cardozo et al., 2025). We did not observe fusions among these muscles in any of our specimens, in which fibrous bands always separated them and were described as intermuscular septa. On the other hand, no authors have reported the origin from the cranial and dorsal margins of the cranial angle of the scapula, as we found in *F. catus*. The insertion onto the lesser tubercle has a typical pattern in most felids, although Pérez & König (Pérez & König, 2022) reported that the Sb tendon fuses with the joint capsule in *F. catus*, similar to *Le. pardalis* (Julik et al., 2012). Interestingly, when we dissected this tendon to review the insertion onto the lesser tubercle, it adhered to the joint capsule. On several occasions, when the tendon is cut, the capsule remains in the tendon, similar to the SS tendon at the lateral aspect of the joint capsule.

#### 3.2.2 M. teres major (TMaj)

The TMaj is a unipennate muscle that originates via aponeurotic fibers from the dorsal third of the caudal margin of the scapula and the caudal surface of the caudal angle of the scapula (Figure 2, Supplementary material 1). It also originates via fleshy fibers from the dorsal two thirds of the intermuscular septum of the most caudal belly of Sb (Figure 5), the lateral and dorsal surfaces of the caudal angle of the scapula (just caudal to the caudal insertion of the m. rhomboides cervicis et thoracis ‐RCeT‐), and the proximal fourth of the caudoproximal extreme of the IS belly (TMaj) (Figures 1 and 2, Supplementary material 1). It inserts via a common tendon with the lateral belly of the latissimus dorsi muscle (LDI) onto the intertubercular groove (Teres major tuberosity) (Figures 3 and 5, Supplementary material 2). The AxN, SbA, CdCHA, and thoracodorsal artery (*A. thoracodorsalis* ‐TD‐) supply it. We did not observe anatomical variants in this muscle.

Most authors report the origin of TMaj from the caudal margin and caudal angle of the scapula and the common insertion with the m. latissimus dorsi (LD) (Barone, 2020; De Iuliis & Pulerà, 2019; Pérez & König, 2022; Reighard & Jennings, 1901; Sebastiani & Fishbeck, 2005). Reighard & Jennings reported additional origins from Sb and IS, which all our specimens had. The TMaj did not have fusions with the Sb but rather an interseptal origin with fibrous bands that separate the fleshy fibers of both muscles. However, these separating fibrous bands can belong to both muscles (FcS2, FcS3, and FcS4) or only to Sb (FcS5 and FcS6). Ross (1883) described the additional origin of Sb in *At jubatus*, and other authors reported that both muscles fuse at this level in other species (Cardozo et al., 2025; Dunn et al., 2022; Julik et al., 2012; Smith et al., 2021). In *F. catus*, Hudson and Hamilton (2010) reported the insertion of TMaj as independent of LD, and the insertion of LD in common with PP. However, the common insertion of LD with both muscles is present in most felids (Vélez‐García et al., 2024), except in *A. jubatus* and *Ly. lynx* where the insertion of TMaj is independent and caudal to the LD based on muscle maps of Hudson et al. (2011) and Viranta et al. (2016), respectively. Furthermore, the TMaj of *A. jubatus* can have a fleshy insertion (Ross, 1883). Although most authors have distinct descriptions of the insertion area, this corresponds to the teres major tuberosity (Concha et al., 2004; da Costa da Silva et al., 2025; Dunn et al., 2022; Smith et al., 2021). For example, Barone (2020) reported that it inserts distally onto the medial lip of the intertubercular groove; Pérez and König (2022), onto the crest of the lesser tubercle; and other authors, onto the proximal region of the medial surface of the humerus (Barone, 1967; Cardozo et al., 2025; De Iuliis & Pulerà, 2019; Reighard & Jennings, 1901; Ross, 1883; Sebastiani & Fishbeck, 2005).

#### 3.2.3 M. coracobrachialis (CB)

The CB is a bipennate muscle that originates via a long tendon from the coracoid process of the scapula and passes medially to the Sb tendon, which is protected by a synovial sheath. Its belly inserts mainly via fleshy fibers onto the caudal aspect of the crest of the lesser tubercle of the humerus and via tendon fibers in its lateral aspect (adjacent to the caput accessorium of m. triceps brachii ‐TBa‐). A small branch of musculocutaneus nerve (*N. musculocutaneus* ‐McN^CB^‐) and CdCHA supply it. As anatomical variants, the CB formed accessory bellies with different arrangements; its innervation was not directly from McN but from other branches of the brachial plexus, and one arterial supply was via a branch of SbA (Table 3).

In one forelimb (FcS8‐RTL), the CB formed two bellies through which the McN passed (Figure 6 and Supplementary material 4.1). In two forelimbs (FcS2 bilaterally), the CB formed a vestigial fleshy belly that joined to the proximal extreme of the belly of the m. biceps brachii (BB) (Figure 6). In six forelimbs, the vestigial bellies corresponded to the long portion of CB (*m. coracobrachialis longus* ‐CBl‐), which was a fusiform belly located medial and distal to the CB and originated from the coracoid process via a common tendon with the CB that passed internally through the CB belly. In five forelimbs (FcS6 bilaterally, FcS7 bilaterally and FcS10‐RTL), an underdeveloped belly formed a distal aponeurosis fused to the deep brachial fascia between the BB and the caput mediale of m. triceps brachii (TBm) (Figure 6 and Supplementary material 4.2). In two of those forelimbs (FcS6‐RTL and FcS7‐LTL), it also inserted onto the origin tendon of TBm (Figure 6). In one limb (FcS3‐RTL), it was a well‐developed unipennate muscle that extended to the supracondylar foramen (SCF) (Figure 6). In this limb, when its tendon of origin crossed through the CB belly, it was divided into two parallel tendons (superficial–medial– and deep–lateral–), between which were the muscle fibers. The deep tendon (lateral) was inserted proximal to the SCF. The superficial tendon (medial) disappeared at the level of the SCF, where a fleshy belly appeared and inserted via fleshy fibers onto the entire proximal margin of the SCF, displacing the *m. anconeus medialis* (AM) distally. In this limb (FcS3‐RTL), the McN innervated the CBl via a branch (McN^CBl^) that originated distally to the proximal muscular branch (*ramus muscularis proximalis* ‐McN^pmb^‐) (Figure 6 and Supplementary material 4.3), whereas in the other three limbs (FcS6‐RTL, FcS7‐LTL, and FcS10‐RTL), that branch originated proximally to McN^pmb^ (Supplementary material 4.2). The innervation in the other limbs and the arterial supply in all limbs were not observed. In one limb (FcS7‐LTL), the arterial supply to the CB was via a branch of SbA that also supplied Sb and TBa (Table 3). This arterial branch corresponded to the branch formed from the CdCHA of other specimens, but in this limb, it originated from SbA.

**FIGURE 6:**
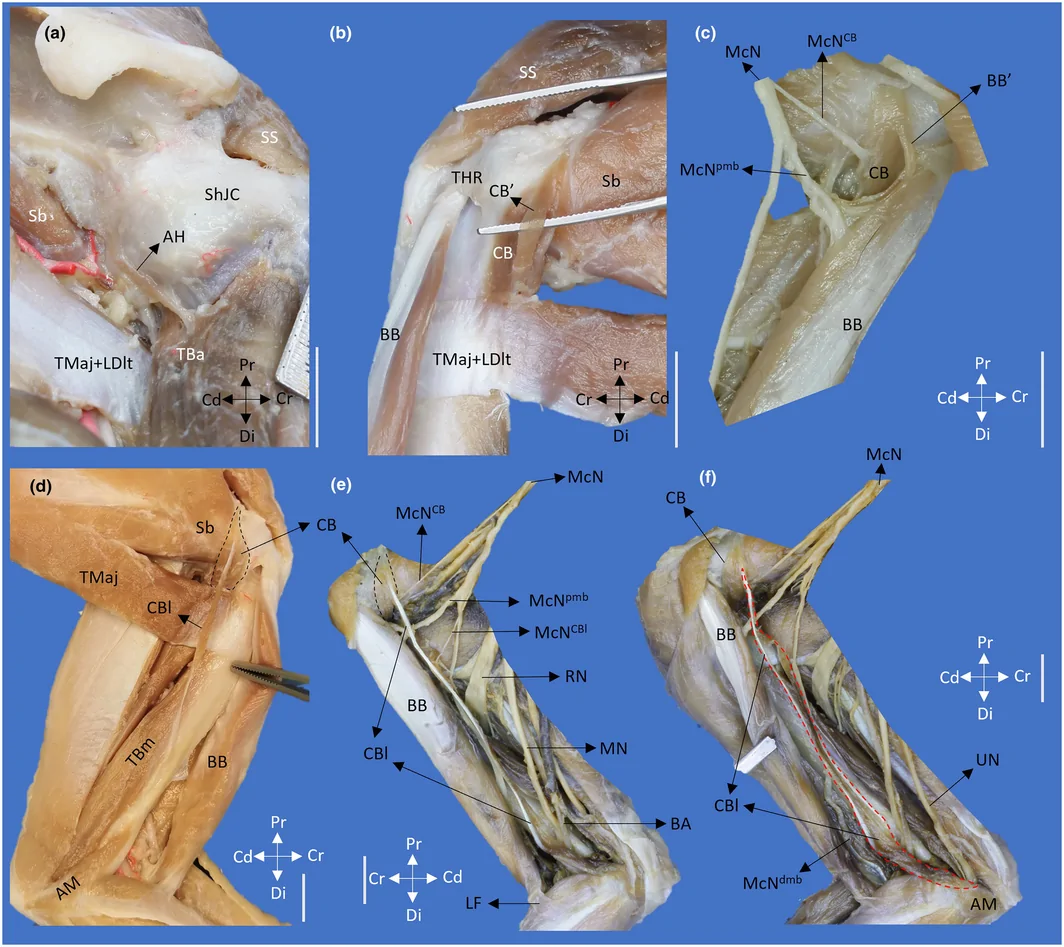
Variations in the articularis humeri, coracobrachialis and biceps brachii muscles of domestic cat (*F. catus*). (a) Caudolateral view of the shoulder joint capsule with the AH; (b) medial view of the right shoulder showing the duplication of the CB; (c) medial view of the left shoulder showing the vestigial caput breve of the BB (BB’); (d) medial view of the left shoulder and brachium showing the vestigial CBl; (e) medial view of the right brachium showing the CBl; (f) medial view of the right brachium delimiting the CBl with red dashed lines. CB’, accessory belly of CB; Cd, caudal; Cr, cranial; Di; distal; LF, lacertus fibrosus; Pr, proximal; TMaj + LDt, common tendon of insertion of TMaj and LDl. White bars: 10 mm.

The CB of most felids originates from the coracoid process, but in *Pa. uncia*, it originates from the medial margin of glenoid cavity (Smith et al., 2021). Barone (2020) described the presence of a complete synovial sheath in the origin tendon, similar to our findings. The descriptions of the CB insertion area vary among authors, but these findings are similar to our findings in *F. catus*. Controversially, Pérez and König (2022) described the insertion between TBm and B, although the relationship with B is impossible due to the presence of TBa. In *Pa. leo*, Barone (1967) reported that it inserts slightly cranially to the common insertion of TMaj and LDl, differing from that reported by Vargas et al. (2017), who described the typical pattern of other felids. In *Pa. uncia*, the CB inserts additionally onto the TBa (Smith et al., 2021). We observed that fascial tissue adhered the CB to the TBa in all specimens. Among felids, the CBl has only been reported in *F. catus* as an anatomical variant (Barone, 2020; Reighard & Jennings, 1901), which we found with a prevalence of 31.58% based on our sample (6/19).

#### 3.2.4 M. articularis humeri (AH)

The AH was observed only in one forelimb (FcS8‐RTL, Table 3), which was a small muscle band formed by parallel fibers located caudally on the shoulder joint capsule (ShJC) (Figure 6). It originated via fleshy fibers from the middle point of the caudal aspect of the margin of the glenoid cavity and inserted via fleshy fibers onto the proximal part of the accessory belly of the m. triceps brachii (TBa) (Figures 2 and 3). The innervation and arterial supply were not observed.

In felids, the AH has been correctly described by Barone (2020) and Pérez and König (2022) in *F. catus* and Barone (1967) and Vargas et al. (2017) in *Pa. leo*. Other authors wrongly described the CB as “*m. articularis humeri*” (Vélez García et al., 2025). Barone (2020) and Pérez and König (2022) do not report AH as an anatomical variant. Furthermore, the NAV does not specify that the presence of AH is variable in *F. catus* since the term “*m. articularis humeri*” is not between parentheses (International Committee on Veterinary Gross Anatomical Nomenclature, 2017). In contrast, we only found AH in one forelimb, which, based on our sample, corresponded to a prevalence of 5.26% (1/19). According to Pérez and König (2022), the AH originates just proximal to the margin of the glenoid cavity and inserts onto the humeral neck, which differs from the unique AH of the present study. Barone (1967) reported in *Pa. leo* that the AH originates from the scapular neck (medial to TBLo tendon) and passes between the fibers of the TBa to reach the humeral neck. We did not find the insertion onto the humerus; the insertion was confined to the proximal part of TBa. Cardozo et al. (2025) reported that the AH described by Barone (1967) in *Pa. leo* could be “a secondary bundle of the m. teres minor” described by Ross (1883) in *A. jubatus*. However, based on the topology of AH, it is not a band of TMin (Vélez García et al., 2025).

### 3.3 Cranial muscles of the brachium

#### 3.3.1 M. biceps brachii (BB)

The BB is a bipennate muscle with one head that originates via a cylindrical tendon from the supraglenoid tubercle that passes within the articular cavity of the shoulder joint and through the intertubercular groove of the humerus (Figures 2 and 5). The BB inserts via a flat tendon onto the caudal aspect of the radial tuberosity protected by a synovial bursa located medial to the radial tuberosity (*Bursa bicipitoradialis* ‐BBR‐) (Figure 7, Supplementary material 3). The muscle belly develops the lacertus fibrosus, which inserts onto the antebrachial fascia over the m. pronator teres (PT), and also develops another aponeurosis that passes medial to the insertion tendon of m. brachialis (B), which inserts onto the cranial aspect of the elbow joint capsule (Figure 5). A second proximal muscular branch of McN (McN^pmb^), the cranial circumflex humeral (*A. circumflexa humeri cranialis* ‐CrCHA‐), bicipital (*A. bicipitalis*), and transverse cubital (*A. transversa cubiti* ‐TCA‐) arteries supply it. The bicipital artery originates from the brachial superficial artery (*A. brachilis superficialis* ‐BSA‐) to supply the BB distally. As anatomical variants, we observed a vestigial caput breve formed from CB, additional aponeurotic fascicles of insertion and additional arterial supply (Table 3). In three limbs, the aponeurosis that inserted onto the elbow joint capsule formed another aponeurotic bundle that inserted onto the insertion tendon of B (Table 3, Supplementary material 4.4). In two limbs, another branch (variant bicipital artery) that originated from the brachial artery also supplied it at the middle level of its belly (Table 3, Supplementary material 4.4).

**FIGURE 7:**
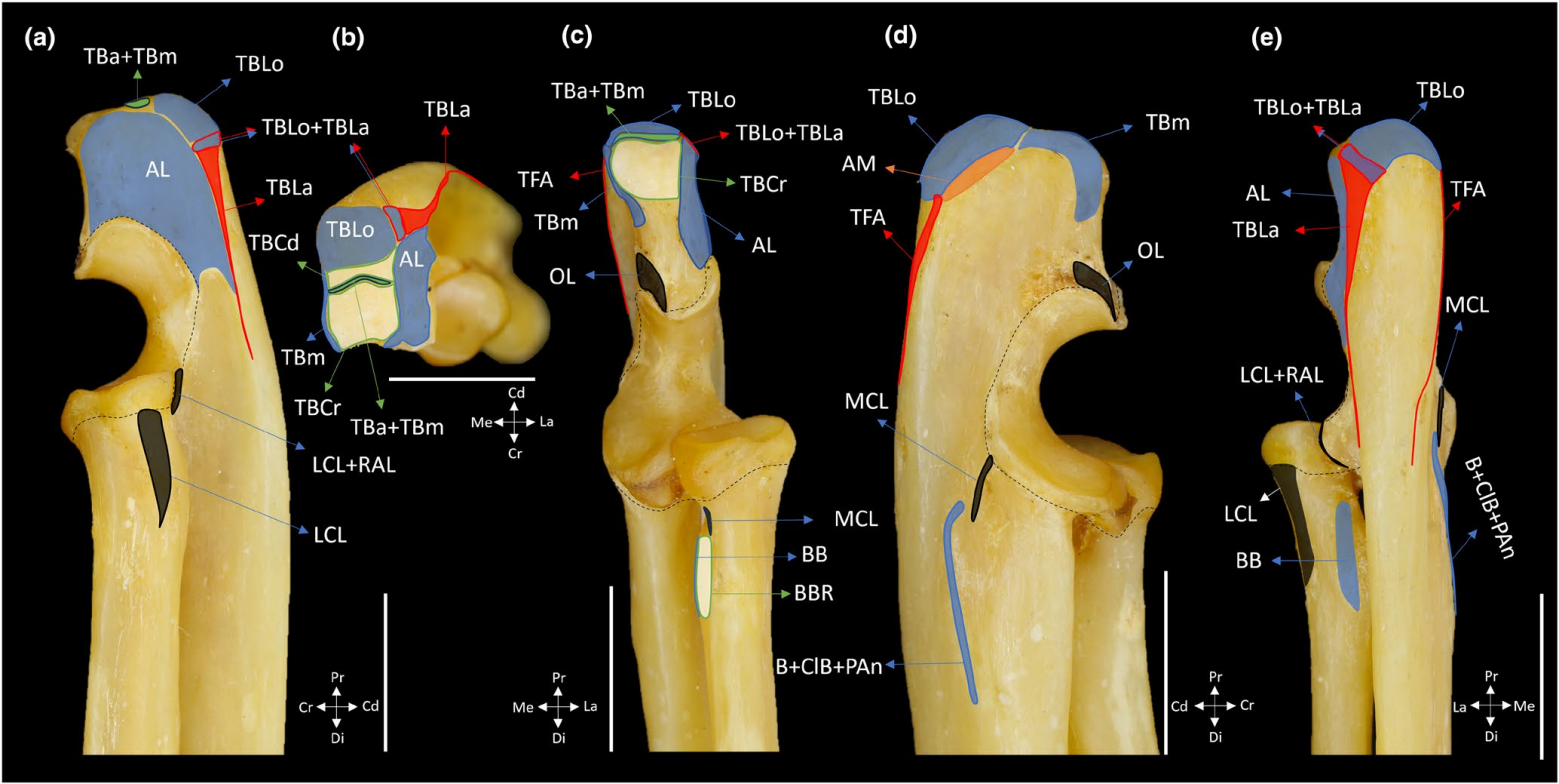
Muscle maps in the proximal extremes of the left radius and ulna of domestic cat (*F. catus*). (a) Lateral, (b) proximal, (c) cranial, (d) medial, and (e) caudal views. BBR, bursa bicipitoradialis; Cd, caudal; Cr, cranial; Di; distal; LCL, lateral collateral ligament of elbow; MCL, medial collateral ligament of elbow; OL, olecranon ligament; Pr, proximal; RAL, radial annular ligament; TBCd, caudal tricipital bursa; TBCr, cranial tricipital bursa. +, common insertion of muscles or ligaments. Dark dashed lines, insertion of the elbow joint capsule. White bars: 10 mm.

The BB in most felids has one head that originates from the supraglenoid tubercle and inserts onto the radial tuberosity. In some felids, it can have an additional insertion onto the ulna, such as *Ly. lynx* (Ari, 2024; Viranta et al., 2016), *Pa. uncia* (Smith et al., 2021), and *A. jubatus* (Böhmer et al., 2020; Hudson et al., 2011; Ross, 1883). Although in *Pa. uncia*, the additional insertion onto the ulna can be absent (Smith et al., 2021). None of the references of *F. catus* reported the presence of the lacertus fibrosus or a similar structure or the variant fascia connecting the tendon to the elbow joint capsule. We found the lacertus fibrosus in all our specimens, similar to *Pa. leo* (Barone, 1967), *Ly. lynx* (Viranta et al., 2016), and *Pa. tigris* (Dunn et al., 2022). The BB belly can have a variable small subdivision in *Le. geoffroyi* (Cardozo et al., 2025) or be divided into two bellies in *Ly. lynx* (Ari, 2024). No authors reported a vestigial caput breve of BB in *F. catus* or other felids.

#### 3.3.2 M. brachialis (B)

The B is a bipennate muscle that originates mainly via fleshy fibers from the craniolateral and caudal surfaces of the humerus, having the shape of an inverted “V.” The craniolateral origin covers the caudal surface to the teres minor tuberosity, deltoid tuberosity, and proximal extreme of humeral crest. The caudal origin covers the proximal three fourths of the caudal surface of the humerus and the proximal two thirds of the medial aspect of the lateral supracondylar crest (medial to the ECR). The B did not have origin from the middle part of the groove of m. brachialis of the humerus (*sulcus m. brachialis*) (Figure 3, Supplementary material 2). The B inserts via a common tendon with the cleidobrachialis (ClB) and pectoantebrachialis (PAn) muscles onto the ulna in a sulcus distal to the insertion of the medial collateral ligament of the elbow (Figures 5 and 7). There is a fat body between the tendons of insertion of B and BB (Supplementary material 4.4). The distal muscular branch of McN (*N. musculocutaneus ramus muscularis distalis* ‐McN^dmb^‐), TCA and collateral radial artery (*A. collateralis radialis* ‐CRA‐) supply it (Figure 4). As anatomical variants, the m. brachiorradialis (BR) fused to the caudal aspect of the B belly in two limbs (FcS10 bilaterally) (Table 3, Supplementary material 4.5), and the radial nerve (RN) innervated it complementarily in one limb (Table 3, Supplementary material 4.5).

Our anatomical findings of B agree with the origin and insertion described by Reighard & Jennings and their muscle maps (Reighard & Jennings, 1901). Hudson and Hamilton (2010) did not represent the origin from the lateral supracondylar crest, similar to several authors who reported only the origin from the humeral shaft (Barone, 2020; De Iuliis & Pulerà, 2019; Pérez & König, 2022; Sebastiani & Fishbeck, 2005). The representation and description of a unique proximal origin of the B from the humeral shaft also occur in other felids (Concha et al., 2004; da Costa da Silva et al., 2025; Hudson et al., 2011; Smith et al., 2021; Viranta et al., 2016). The B forms an intermuscular septum to separate it from TBa in *F. catus*, while the origin of B can partially fuse with TBm in *Pa. uncia* (Smith et al., 2021) or tendon of origin of TBLa in *Le. pardalis* (Julik et al., 2012). Based on the muscle maps of *Pa. tigris* (Dunn et al., 2022), the B originates from the entire lateral surface of the humeral shaft without having an origin with a “V” shape, differing from *F. catus* (Reighard & Jennings, 1901, our results) and other species (Cardozo et al., 2025; Julik et al., 2012; Smith et al., 2021). Hudson and Hamilton (2010) schematized an insertion more proximally than most of the studies in *F. catus* and other felids. Pérez and König (2022) reported that B inserts via a common tendon with the ClB onto the radius, which differs from our study and most studies in *F. catus*. Furthermore, the common insertion also involves the superficial part of the m. pectoralis descendens (m. pectoantebrachialis) in *F. catus* (Vélez‐García et al., 2024), *Pa. uncia* (Smith et al., 2021), *Pa. tigris* (Dunn et al., 2022), and *Le. geoffroyi* (Cardozo et al., 2025). In *A. jubatus*, three tendons of the B have been described, two of which insert onto the ulna, and another onto the BB tendon (Ross, 1883). However, common insertion onto the ulna of B with other muscles was not reported by the cited authors (Böhmer et al., 2020; Hudson et al., 2011; Ross, 1883).

### 3.4 Caudal muscles of the brachium

#### 3.4.1 M. tensor fasciae antebrachii (TFA)

The TFA is a flat, parallel‐fibred muscle located medially in the tricipital region. It originates via fleshy fibers from the lateral belly of the m. latissimus dorsi (LD) and via aponeurotic fibers from the common tendon of several extrinsic thoracic limb muscles (medial belly of the m. latissimus dorsi ‐LDm‐, caudal part of the m. pectoralis profundus ‐PPCd‐, m. pectoralis abdominalis ‐PAb‐, and m. cutaneus trunci ‐CT‐) (Figure 8). The TFA inserts via an aponeurosis onto the antebrachial fascia, the tendon of the caput longum of the m. triceps brachii (TBLo), and the medial margin of the olecranon (adjacent to the TBLo tendon) (Figures 7 and 8). The radial nerve innervates it via a branch that also extends to the TBLo. The deep brachial (DBA) and superficial brachial (SBA) arteries supply it. As anatomical variants, we observed four variants (Table 3). In one limb (FcS1‐LTL), the TFA also originated via a weak fascicle from the TMaj (Figure 1). In one limb (FcS5‐LTL), the TFA was divided into two bellies, one cranial that originates from the common tendon of the extrinsic muscles (LDm, PPCd, PAb, and CT) and one caudal from LDl. In addition, in that same limb, the TFA did not insert onto the TBLo tendon (Supplementary material 4.6). In one limb (FcS8‐LTL), the TFA was divided into two bellies, superficial and deep, where the former had the typical origin, whereas the deep belly only originated from the common tendon of LDl and TMaj. Both bellies were fused caudally and distally.

**FIGURE 8:**
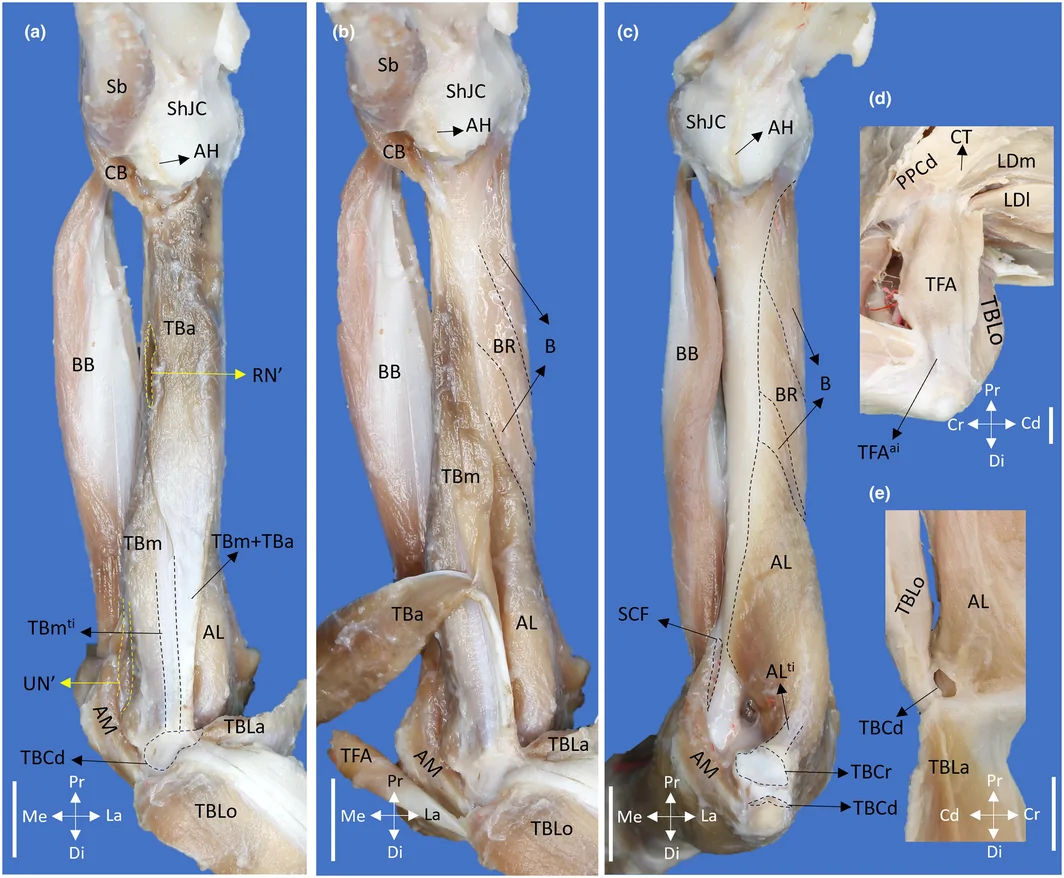
(a) Caudal view of a right brachium after retracting the TFA, TBLo, and TBLa distally; (b) caudal view of a right brachium after retracting the TBa distally; (c) caudal view of a right brachium after eliminating the TFA and TB; (d) medial superficial view of a right brachium to observe the TFA; (e) lateral view of the elbow to observe the caudal tricipital bursa (TBCd). AL^ti^, tendinous fibers of AL insertion; BR, m. brachioradialis; Cd, caudal; Cr, cranial; Di; distal; La, lateral; Me, medial; Pr, proximal; RN’, the proximal yellow dashed lines are indicating the intermuscular cleft between TBa and TBm for the passage of the RN; SCF, supracondylar foramen; ShJC, shoulder joint capsule; TBCd, caudal tricipital bursa; TBCr, cranial tricipital bursa; TBm + TBa, common tendon of insertion of TBm and TBa; TBm^ti^, tendon of insertion of TBm; TFA^ai^, aponeurosis of insertion of TFA; UN’, the distal yellow dashed line indicates the space for the passage of the UN between the AM and TBm. White bars: 10 mm.

Formerly, the TFA was named “*m. epitrochlearis*” (Reighard & Jennings, 1901; Sebastiani & Fishbeck, 2005), and some authors continue using that name (Böhmer et al., 2020; De Iuliis & Pulerà, 2019). Reighard and Jennings (1901) reported that the TFA originates only from LD close to the insertion of CT and, as anatomical variants, from the TMaj and PP. We found a typical pattern in which TFA originated from both common tendons formed by the LD bellies (LDl and LDm) with other muscles and from TMaj in one limb. Barone (2020) reported a similar origin from both bellies of LD, although the author described only the second common tendon with CT, which is also with the PPCd in *F. catus* (Vélez‐García et al., 2024) and, similarly, in *Ly. lynx* (Viranta et al., 2016). Pérez and König (2022) described it only as a muscle that originates from LD and CT. Other authors describe the origin only from LD (De Iuliis & Pulerà, 2019; Sebastiani & Fishbeck, 2005), similar to *Pa. tigris* (Dunn et al., 2022). Interestingly, the TFA in the *Pa. leo* can form three bellies with separate origins from the IS, TMaj, and LD (Barone, 1967) or from the scapular spine, caudal margin of the scapula, and LD (Vargas et al., 2017). The weak variant that originated from the TMaj in one limb of *F. catus* (FcS1‐LTL) is similar to the middle belly of *Pa. leo* since this also originates from the TMaj (Barone, 1967). Concha et al. (2004) in *Pu. concolor* reported a wide range of origin, covering the scapular spine, caudal margin of the scapula, LD, and TMaj. No authors have reported the duplication of the TFA into deep and superficial bellies, as we observed in one limb of *F. catus* (FcS8‐LTL). In *Pa. uncia*, in addition to originating from the superficial fascia of the LD, it also originates from the fascia of the TBm (Smith et al., 2021). The available muscle maps in *F. catus* do not represent the TFA insertion onto the olecranon (Hudson & Hamilton, 2010; Reighard & Jennings, 1901). We represented it based on the continuation of the insertion aponeurosis of the TFA until the olecranon and proximal part of the ulnar shaft. In other felids, the authors do not represent the TFA insertion because the muscle does not have bony insertions (Dunn et al., 2022; Julik et al., 2012; Smith et al., 2021). Cardozo et al. (2025) described the additional insertion onto the olecranon but did not represent it in their muscle maps. The additional insertion onto the olecranon is also reported in *Pa. leo* (Barone, 2020; Vargas et al., 2017) and *Ly. lynx* (Viranta et al., 2016). An additional insertion occurs onto the TBm in *Le. pardalis* (Julik et al., 2012) and *Pa. uncia* (Smith et al., 2021), in addition to fusing with the cleidobrachialis muscle in the former species (Julik et al., 2012). The muscle is absent in *A. jubatus* (Böhmer et al., 2020; Hudson et al., 2011) and only appears as an anatomical variant since it has been found in two specimens (Böhmer et al., 2019; Ross, 1883). Ross (Ross, 1883) described it as a “triceps accessorius” inserting onto the olecranon. da Costa da Silva et al., 2025 did not report TFA in three felid species they dissected (*Pu. concolor*, *Le. Pardalis*, and *Le. wiedii*), whereas previous studies confirmed its presence in two of them (*Pu. concolor* and *Le. pardalis*) (Concha et al., 2004; Julik et al., 2012).

#### 3.4.2 M. triceps brachii (TB)

The triceps brachii muscle has four heads: long (*caput longum* ‐TBLo‐), lateral (*caput laterale* ‐TBLa‐), medial (*caput mediale* ‐TBm‐), and accessory (*caput accessorium* ‐TBa‐). The radial nerve innervates all four heads.

The TBLo is a bipennate head that originates via a strong tendon from the caudal margin of the scapula and the infraglenoid tubercle. In most specimens, additional oblique tendinous bands reinforced this origin (Figures 2 and 4). The tendon of origin is developed strongly at the level of the infraglenoid tubercle, and is flat with fleshy fibers internally that also originate from the caudal margin. The TBLo inserts via a tendon onto the caudoproximal aspect of the olecranon tuberosity, which also fuses with the TBLa tendon laterally, proximal to their insertions (Figures 7 and 8). The SbA, CdCHA, CRA, DBA, and collateral ulnar artery (CUA) supply it. The radial nerve innervates it via two branches, one of which also extends to the TFA (Figure 4). Two anatomical variants were observed in this head (Table 3): In one limb (FcS6‐RTL), the oblique fascicles of the tendon of origin were absent. In one limb (FcS5‐LTL), the TBLo also originated via a tendinous fascicle from the fascia over the IS, which passed laterally to the TMin and joined caudally to the main tendon of origin (Supplementary material 4.7).

The TBLa is a fusiform head that originates via an aponeurosis from the brachial fascia, caudal to the deltoid tuberosity, tricipital line, and caudolateral aspect of the humeral neck (proximally adjacent to the origin of the TBa). It sends oblique fleshy fibers to TBLo at the middle of the brachium and forms a tendon that has an aponeurotic shape (Figures 1 and 3). This tendon inserts onto the caudolateral margin of the olecranon (covering the *m. anconeus lateralis* ‐AL‐) and is medially fused with the TBLo tendon to insert onto the caudolateral surface of the olecranon tuberosity (Figures 7 and 8). The CdCHA and CRA supply it (Figure 4). In one limb (FcS8‐RTL), the DBA and CUA also supply it.

The TBa is a bipennate head that originates via fleshy fibers from the entire caudal surface of the humeral neck (between the CB and TBLa), the proximal quarter of the caudal surface of the humeral shaft, and the intermuscular septum with B (IM^B‐TBa^). The TBa forms a tendon in the middle of the brachium, which also receives fleshy fibers from TBm. It inserts via a common tendon with TBm onto the cranial aspect of the olecranon tuberosity (Figure 7, Supplementary material 3). The CdCHA, CRA, and DBA supply it. As an anatomical variant, one limb (FcS4‐LTL) had intermixed fleshy fibers with the proximal part of TBm and originated via tendon fibers from the IM^B‐TBa^ (Table 3).

The TBm is a unipennate head located distal to TBa and originates via an aponeurosis from the proximal second quarter of the medial surface of the humeral shaft. In addition, fleshy fibers originate from the second to third sixths of the caudal surface of the humeral shaft, medial and proximal to its aponeurosis of origin (Figure 5). The fleshy belly of TBm joins obliquely to TBa tendon at the distal third of the brachium, which inserts onto the proximal surface of the olecranon tuberosity, cranial to the origin of the TBLo tendon. The distal fleshy fibers of TBm insert directly onto the craniomedial aspect of the olecranon and via a tendon onto the proximal and cranial part of the olecranon tuberosity. This latter tendon is formed parallel to the common tendon with the TBa (Figures 7 and 8). The DBA, CUA, and a branch directly from the brachial artery (BA) supply it. As an anatomical variant, it additionally originated via a tendinous fascicle from the intertubercular groove, cranial to the common tendon of TMaj and LDl, in one limb (FcS4‐LTL) (Table 3, Supplementary material 4.7).

There were two synovial bursae between the triceps brachii tendons, caudal, and cranial. The caudal synovial bursa was between the TBLo tendon (caudal limit) and the AL and the common tendon of the TBm and TBa (cranial limit). The cranial synovial bursa was between the cranial part of the olecranon tuberosity (cranial limit) and the AL tendon and common tendon of TBa and TBm (caudal limit) (Figures 7 and 8). Cranial to this bursa, there was a fat pad between AL and TBm (Supplementary material 4.8).

Our descriptions and muscle maps align with those of Reighard and Jennings (1901). However, they described the TBm as divided into three portions: intermediate, long, and short, which correspond to the TBm, TBa, and AM of the present study, respectively. The origins of TBm and TBLo via tendon fibers and the insertion of TBm via fleshy fibers differ from those of other authors (Reighard & Jennings, 1901). In contrast, we found a mixed origin with tendon and fleshy fibers in both heads and a mixed insertion into TBm in *F. catus*. Some authors have described TB in less in *F. catus*. Pérez and König (2022) do not describe TBa. Hudson and Hamilton (2010) schematized a longer origin of TBm than Reighard and Jennings (1901) and our findings. In addition, they named TBa the “intermediate head,” and the insertions were not represented separately (Hudson & Hamilton, 2010). Barone (2020) reported that the TBLo origin is restricted to the infraglenoid tubercle and the adjacent part of the scapular neck, which differs from our study and others, where the caudal margin is also part of the origin. The presence of two synovial bursae has only been reported by Reighard and Jennings (1901), similar to our findings. The four heads of TB have similar arrangements in most felids. The origin of TBLo via fleshy and tendon fibers is also present in *Le. pardalis* (Julik et al., 2012) and *Le. geoffroyi* (Cardozo et al., 2025), and TBm insertion occurs only via fleshy fibers in the latter species (Cardozo et al., 2025). Interestingly, Cardozo et al. (2025) described a strong aponeurosis at the origin of TBLo that partially fuses to the origin aponeurosis of TMin, which we identified as the oblique fibers of the origin tendon of TBLo in *F. catus*. In *Pu. concolor*, Concha et al. (2004) did not describe TBa, but more recent studies in this species reported its presence (Barreto‐Mejía et al., 2022; da da Costa da Silva et al., 2025).

#### 3.4.3 M. anconeus lateralis (*m. anconeus* ‐AL‐)

The AL is a pyramidal muscle that originates via fleshy fibers from the distal third of the caudal surface of the humeral shaft, the proximal zone around the olecranon fossa, the lateral supracondylar crest, and the lateral epicondyle (Figures 3, 4, and 8). It also originates via a few fleshy fibers from the caudal aspect of the tendon of origin of the m. extensor carpi ulnaris (ECU). It inserts via tendon fibers onto the proximolateral part of the olecranon tuberosity and via fleshy fibers onto the lateral surface of the olecranon (proximal to the m. extensor digiti I et II ‐EDI‐II‐) (Figure 7, Supplementary material 3). The AL fuses deeply to the lateral surface of the elbow joint capsule. The CRA, CUA, and recurrent branch of the cranial interosseus artery supply it. As anatomical variants, we observed differences in the area of insertion (Table 3). In four limbs (FcS1 bilaterally and FcS6 bilaterally), the insertion covered the proximal part of the m. extensor digiti I et II (EDI‐II). In two of those limbs (FcS6 bilaterally), it extended more distally, reaching the level of the lateral coronoid process of the ulna. In four limbs (FcS3 bilaterally and FcS4 bilaterally), the insertion caused a proximal bifurcation of the origin of EDI‐II.

Reighard and Jennings (1901) did not report the types of origin and insertion (fleshy or tendon fibers) of the AL. We found that the origin of AL extended more proximally from that represented by Reighard and Jennings (1901) but did not reach the distal half of the caudal surface of the humerus as that reported by Pérez and König (2022) or the more distal origin represented by Hudson and Hamilton (2010). Reighard & Jennings (1901) reported extension to the medial epicondyle of the humerus as a variant, whereas we found this extension in all the specimens. Sebastiani and Fishbeck (2005) reported the AL origin from the medial epicondyle as the only origin. The origin reaching the distal part of the humeral shaft is also found in the *Pa. leo* (Barone, 1967), *Pa. tigris* (Dunn et al., 2022), *Pa. uncia* (Smith et al., 2021), *Pu. concolor, Le. pardalis*, and *L. wiedii* (da da Costa da Silva et al., 2025). However, among these species, the specific zone covering the humeral shaft is described as the distal half in *Pa. leo* (Barone, 1967), *Le. pardalis* (Julik et al., 2012), *Le. geoffroyi* (Cardozo et al., 2025), the distal third in *Ly. lynx* (Ari, 2024), and the distal quarter in *Pa. uncia* (Smith et al., 2021). We observed that the AL originated from slightly less than the distal half of the humeral shaft in *F. catus*, similar to *Le. geoffroyi* (Cardozo et al., 2025). We only observed one belly in *F. catus*, whereas other species can have two or three bellies, such as *Pu. concolor* (Concha et al., 2004), *Le. pardalis* (Julik et al., 2012), *Pa. uncia* (Smith et al., 2021), and *Le. geoffroyi* (Cardozo et al., 2025). No authors have described the origin from the adjacent tendon of origin of the ECU, although Julik et al. (2012) reported a fusion with it in *Le. pardalis*. Ross (1883) reported the AL as absent in *A. jubatus*, while other authors reported it in that species (Böhmer et al., 2020; Hudson et al., 2011). In *A. jubatus*, the muscle originates almost exclusively from the distal quarter (Hudson et al., 2011). We found that it fused with the elbow joint capsule in *F. catus*, similar as described in *Le. pardalis* (Julik et al., 2012) and *Ly. lynx* (Ari, 2024). Reighard and Jennings (1901) reported it as an adhesion in the elbow joint capsule, similar to Concha et al. (2004) in *Pu. concolor*.

#### 3.4.4 M. anconeus medialis (AM)

The AM is a triangular muscle that originates via fleshy fibers from the entire proximal margin of the supracondylar foramen (SCF) and the distal two thirds of the caudal aspect adjacent to this margin. It also originates via tendon fibers from the medial epicondyle of the humerus (AM^to^) (Figure 9). The AM did not originate from the proximal third of the caudal aspect of the supracondylar foramen because the BA and median nerve (MN) passed at this level (Figure 5). The AM inserts via fleshy fibers onto the medial margin of the olecranon tuberosity and via tendon fibers onto the proximal part of the olecranon tuberosity (AM^ti^) (Figures 7, 8, and 9). The ulnar nerve (UN) (Figure 9, Supplementary material 4.9), CUA and a recurrent branch of the caudal interosseous artery supply it. As anatomical variants, we observed differences in the insertion and an additional arterial supply (Table 3). In one limb (FcS3‐RTL), the CBl displaced it distally and caudally, and because of this, the AM originated from the distal half of the caudal surface of the SCF (without originating from the proximal margin of the SCF). In six limbs, fleshy fibers inserted proximally to its tendon fibers. In one limb (FcS7‐LTL), the BA also supplied it proximal to the SCF.

**FIGURE 9:**
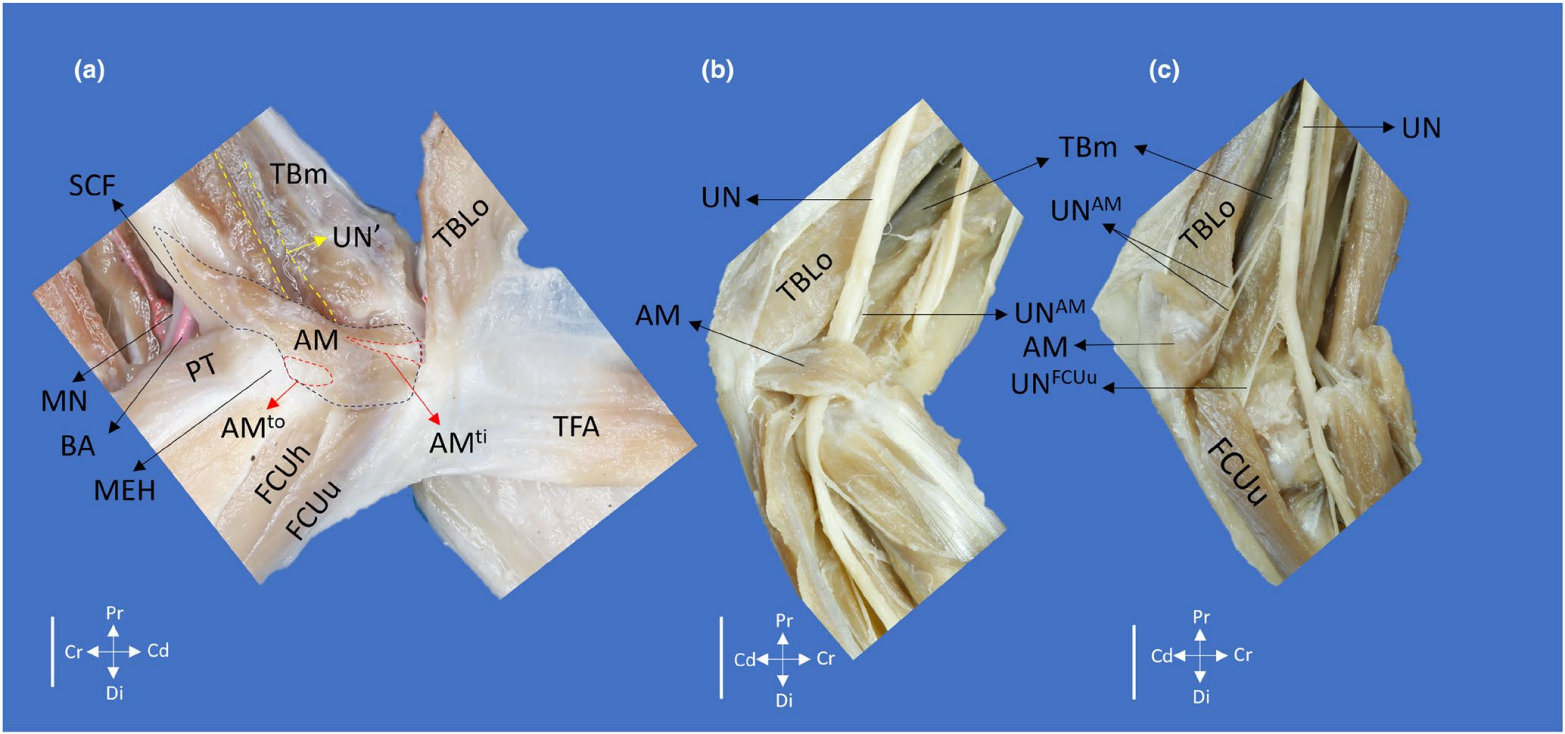
Medial views of the topology and innervation of the AM in domestic cat (*F. catus*). (a) Medial view of a right elbow retracting caudally and distally from the TFA and TBLo. (b) Medial view of a left elbow to observe the passage of the ulnar nerve medial to the AM. (c) Medial view of a left elbow retracting caudally from the AM to observe its innervation by the ulnar nerve (UN^AM^). AM^ti^, tendinous insertion of AM; AM^to^, tendinous origin of AM; BA, brachial artery; Cd, caudal; Cr, cranial; Di; distal; FCU, m. flexor carpi ulnaris; FCUh, caput humerale of FCU; FCUu, caput ulnare of FCU; MEH, medial epicondyle of the humerus; Pr, proximal; PT, m. pronator teres; SFC, supracondylar foramen; UN^AM^, branches of UN to AM; UN^FCUu^, branch of UN to FCUu; UN’, impression of the UN on the TBm. White bars: 10 mm.

Barone (1967, 2020) is the only author who uses the term AM (from the French language “anconé medial”), which we agree to use for evolutionary and embryological reasons. Other authors named it a short part of the TBm in *F. catus* (Hudson & Hamilton, 2010; König & Pérez, 2022; Reighard & Jennings, 1901; Sebastiani & Fishbeck, 2005) or the TB caput mediale accessorium in other felids (Cardozo et al., 2025; Dunn et al., 2022; Julik et al., 2012; Smith et al., 2021; Viranta et al., 2016). Reighard and Jennings (1901) reported the same origin as our study but did not describe the type of origin (fleshy or tendon), and they described the insertion via only fleshy fibers. We found that it originated only via fleshy fibers and inserted via fleshy and tendon fibers. König and Pérez (2022) recognized that the short portion of TBm is the “m. epitrochleoanconeaus.” Even in other felids, authors use it as a synonym for “caput mediale accessorium” (Cardozo et al., 2025; Smith et al., 2021) and include another synonym, namely “anconeus internus” (Cardozo et al., 2025; Ross, 1883). Böhmer et al. (2020) wrongly named it “caput accessorium” in their atlas because the actual TBa is labelled together with TBm. However, they confirmed the presence of this muscle in *A. jubatus* (Böhmer et al., 2020), similar to the findings of Ross (1883), while Hudson et al. (2011) did not report it. In *Ly. lynx*, Viranta et al. (2016) reported that the AM originated only proximal to the supracondylar foramen and was absent in two of six specimens. Ari (2024) reported that it originated from the medial supracondylar crest (the author should refer to the supracondylar foramen) in the two *Ly. lynx* studied.

### 3.5 Brachial plexus

#### 3.5.1 Origin of brachial plexus nerves

In most limbs (13/16), the brachial plexuses originate from the ventral branches of the last three cervical spinal nerves (C6, C7, and C8) and the first thoracic spinal nerve (T1) (Figure 10). In two limbs (FcS3 bilaterally), the brachial plexus also originated from the fifth cervical nerve (C5‐T1) (Figures 11 and 12), and in one limb (FcS4‐RTL), it also originated from the second thoracic nerve (C6‐T2) (Figure 12, Table 4). The origin from C6‐T1 in most of our specimens is in agreement with other studies in the same species (Aubert et al., 2004; Ghoshal & Magilton, 1972; Hakkı Nur et al., 2020; Pérez & König, 2022; Reighard & Jennings, 1901; Roos & Vollmerhaus, 2005; Sebastiani & Fishbeck, 2005; Silva & Sánchez, 2013) and other felids (Barreto‐Mejía et al., 2022; Chagas et al., 2014; Grzeczka et al., 2024; Hall et al., 2023; Silva & Sánchez, 2013; Takcı & Arı, 2025). In *F. catus*, Aubert et al. (2004) found only one brachial plexus originating from C5 to T1 in a sample of 40 limbs (20 specimens) (2.5%), while the contribution of T2 was not observed. This latter contribution was observed by other authors who did not report the number of *F. catus* specimens (Ghoshal & Magilton, 1972; Pérez & König, 2022; Roos & Vollmerhaus, 2005). Other studies that specified the number of specimens did not find a contribution from C5 or T2 (Hall et al., 2023; Silva & Sánchez, 2013). Therefore, combining the results of these studies and our study (40 of Aubert et al., 2004; 4 of Hall et al., 2023; 12 of Silva & Sánchez, 2013; and 16 of the present study), the prevalence of C5 (C5‐T1) or T2 (C6‐T2) contributing to brachial plexus in *F. catus* is 4.17% (3/72) and 1.39% (1/72), respectively. In contrast, the contribution from C5‐T1 is greater in the felids *Herpailurus yagouaroundi* (57% Souza Junior et al., 2022) and *Le. geoffroyi* (66.66% Souza‐Junior et al., 2018). In other felids, no authors reported the contribution of C5 or T2 (Barreto‐Mejía et al., 2022; Chagas et al., 2014; Grzeczka et al., 2024; Hall et al., 2023; Silva & Sánchez, 2013; Takcı & Arı, 2025). Controversially, Hakkı Nur et al. (2020) reported an origin from C5 that contributed to the origin of SSN in one of five *F. catus* specimens in the Results section (20%). However, in their table of origins and Conclusion section, they reported that C5 never contributed to their sample (10 limbs from five specimens) and the additional contribution to the origin of SSN was only from C7 (Hakkı Nur et al., 2020).

**FIGURE 10:**
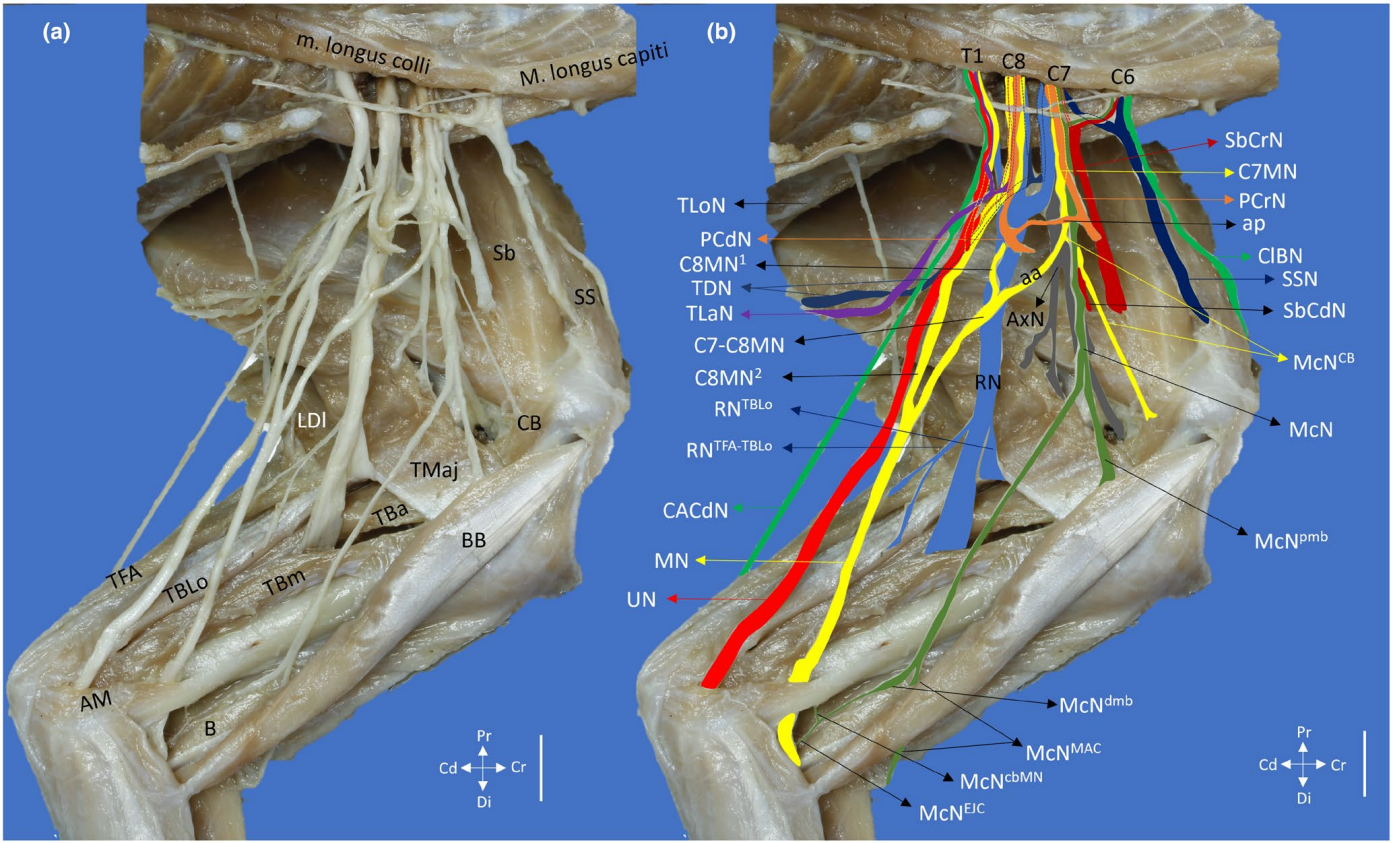
Medial view of the origin and distribution of the brachial plexus nerves in the shoulder and brachium in the left forelimb of a domestic cat (*F. catus*). (a) Photo indicating the muscles without indicating the nerves; (b) photo indicating the nerves and their origin and distribution. aa, ansa axillaris; ap, ansa pectoralis; C7MN, branch of C7 to give rise to the MN; C7‐C8MN, branch formed by C7 and C8 to give rise to the MN; C8MN^1^, cranial branch of C8 to give rise to the MN; C8MN^2^, caudal branch of C8 to give rise to the MN; Cd, caudal; Cr, cranial; Di; distal; McN^CB^, branch to CB (In this case, the branch did not originate from the musculocutaneus nerve but from the ansa axillaris); Pr, proximal. White bars: 10 mm.

**FIGURE 11:**
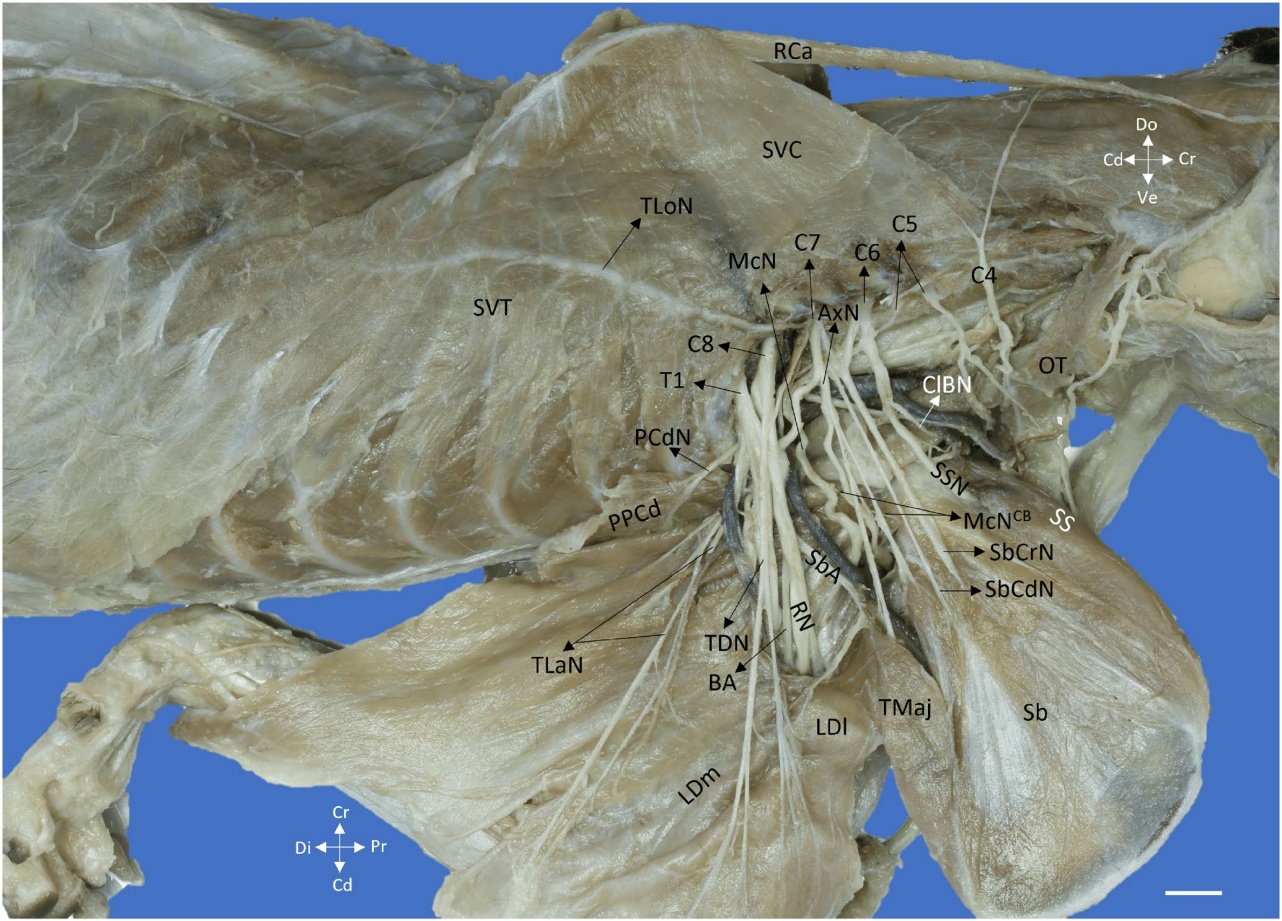
Dorsolateral view of a right brachial plexus with contribution of C5 after displacing the right thoracic limb laterally in a domestic cat (*F. catus*). BA, brachial artery; SbA, subscapular artery; Cd, caudal; Cr, cranial; Di; distal; Do, dorsal; La, lateral; Me, medial; Pr, proximal; Ve, ventral. White bars: 10 mm.

**FIGURE 12:**
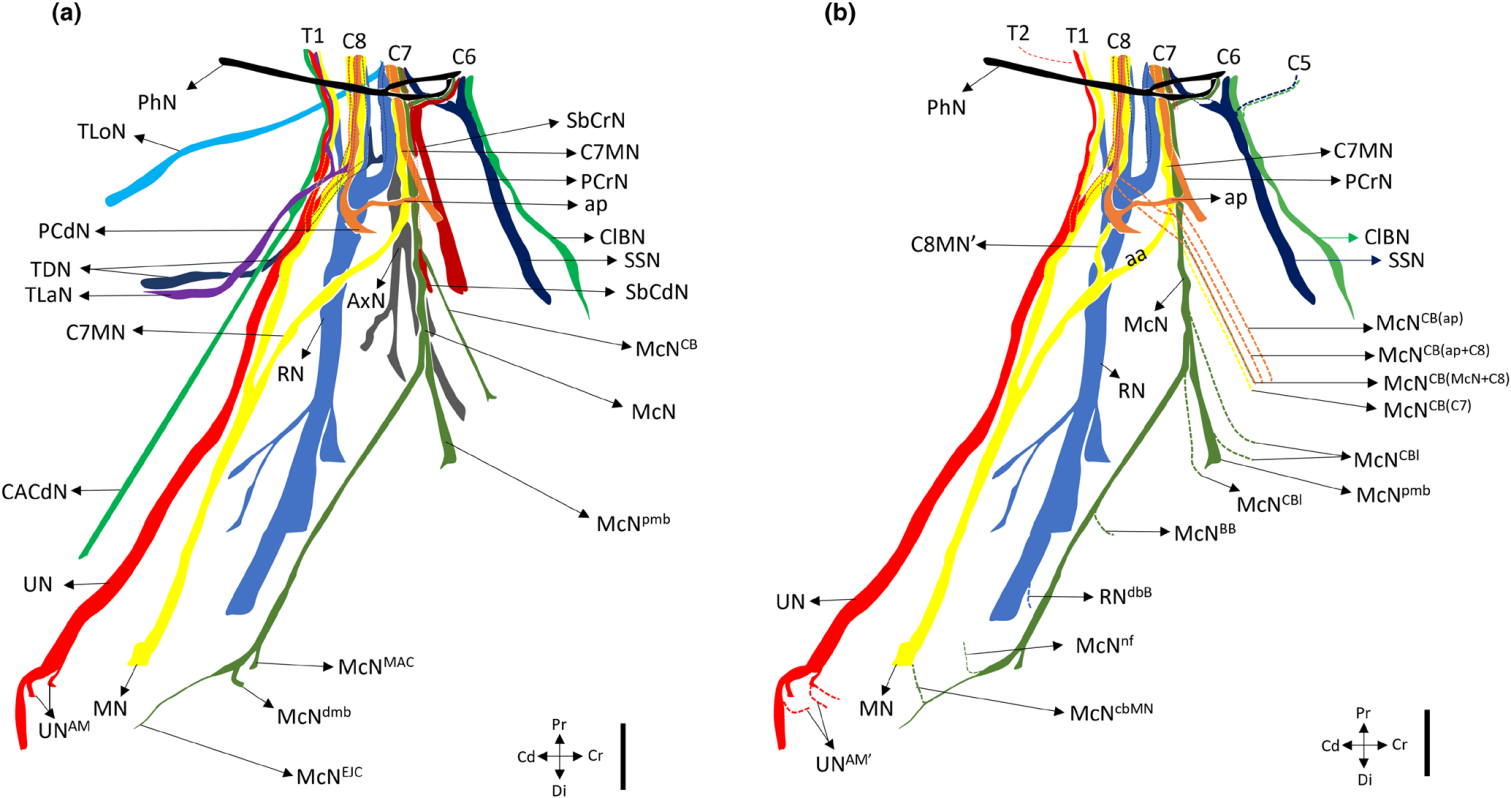
Brachial plexus schemes of domestic cat (*F. catus*). (a) Typical pattern of the brachial plexus. (b) Anatomical variants of brachial plexus nerves. aa, ansa axillaris; ap, ansa pectoralis; C7MN, branch of C7 to give rise to the MN; C7‐C8MN, branch formed by C7 and C8 to give rise to the MN; C8MN’, variant branch of C8 to give rise to MN; Cd, caudal; Cr, cranial; Di; distal; McN^CB(ap)^, branch to CB from ansa pectoralis; McN^CB(ap+C8)^, branch to CB from ap and C8; McN^CB(McN+C8)^, branch to CB from McN and C8; McN^CB(C7)^, branch to CB from C7; McN^CBl^, variant origins of branch to ClB; McN^nf^, branch of McN to nutrient foramen of humerus; PhN, phrenic nerve (*N. phrenicus*); Pr, proximal; RN, radial nerve; RN^dbB^, branch of the deep branch of RN to B; UN^AM’^, variant branches of UN to AM. Black bars: 10 mm.

**TABLE 4:** Origin and distribution of the brachial plexus in the domestic cat (*Felis catus*).

Nerve (abbreviation)	Origin	% (cases)	Muscle innervation
Brachial plexus	C5‐T1	12.5 (2/16)	
	C6‐T1	81.25 (13/16)	
	C6‐T2	6.25 (1/16)	
ClBN	C5‐C6	12.5 (2/16)	ClB
	C6	87.5 (14/16)	
SSN	C5‐C6	12.5 (2/16)	SS and IS
	C6	87.5 (14/16)	
SbCrN	C6	6.25 (1/16)	Sb (bellies 1–3)
	C6‐C7	93.75 (15/16)	
SbCdN	C6‐C7	68.75 (11/16)	Sb (bellies 3–5)
	C7	31.25 (5/16)	
McN	C6‐C7	100 (16/16)	CB, (CBl), B, and BB
McN^CB^ *	McN	(6/14)	CB
	C7	7.14 (1/14)	
	McN and C7MN	7.14 (1/14)	
	McN and C8	14.26 (2/14)	
	C7 (ap) and C8	7.14 (1/14)	
	2 McN^CB^	7.14 (1/14)	
	C7MN‐C8(ap)	7.14 (1/14)	
	C8(ap)	7.14 (1/14)	
AxN	C6‐C7	81.25 (14/16)	Sb, TMaj, TMin, (AH), Ds, Da, and ClB
	C6‐C8	6.25 (1/16)	
	C7‐C8	6.25 (1/16)	
RN	C6‐T1	31.25 (5/16)	TFA, TBLo, TBm, TBa, TBLa, AL, (B), BR, ECRL, ECRB, EDC, EDL, ECU, S, AbDIL, and EDI‐II
	C7‐T1	62.5 (10/16)	
	C6‐C8	6.25 (1/16)	
MN	C7‐T1	100 (16/16)	PT, FCR, FDS, FDPhsl, FDPhsm, FDPhp, FDPr, IF, and PQ
UN	C8‐T1	93.75 (15/16)	AM, FCUh, FCUu, FDPhsl, FDPu, and FDB.
	C8‐T2	6.25 (1/16)	
CACdN	C8‐T1	6.25 (1/16)	
	T1	93.75 (15/16)	
PCrN	C6‐C7	12.5 (2/16)	PAn, PD, and PTr
	C7	75 (12/16)	
	C7‐C8(ap)	12.5 (2/16)	
Ansa pectoralis*	C7	20 (3/15)	
	C7MN	33.33 (5/15)	
	PCrN	20 (3/15)	
	C7MN and PCrN	13.33 (2/15)	
	PCdN	13.33 (2/15)	
PCdN*	C7‐C8	60 (9/15)	PPCr and PPCd, (PTr)
	C7‐T1	26.67 (4/15)	
	C8‐T1	13.33 (2/15)	
TDN	C7‐C8	81.25 (13/16)	LD
	C8	6.25 (1/16)	
	C8‐T1	12.5 (2/16)	
TLaN*	C8‐T1	100 (15/15)	PAb and CT
TLoN	C7	93.75 (15/16)	SVT
	C7‐C8	6.25 (1/16)	

*Note*: (ap) branch from ansa pectoralis; (2 McN^CB^) two branches to CB; The muscles between parentheses indicate the variant innervation (AH, CBl, B and PTr).

Abbreviations: BR, m. brachioradialis; ECRB, m. extensor carpi radialis brevis; ECRL, m. extensor carpi radialis longus; ECU, m. extensor carpi ulnaris; EDC, m. extensor digitorum communis; EDI‐II, m. extensor digiti I et II; EDL, m. extensor digitorum lateralis; FDB, m. flexor digitorum brevis, FDP, m. flexor digitorum profundus; FDPhsl, caput humerale superficiale laterale of FDP; FDPhsm, caput humerale superficiale mediale of FDP, FDPhp, caput humerale profunda of FDP; FDS, m. flexor digitorum superficialis; FCR, m. flexor carpi radialis; FCU, m. flexor carpi ulnaris; FCUh, caput humerale of FCU; FCUu, caput ulnare of FCU; IF, mm. interflexorii; PQ, m. pronator quadratus; PT, m. pronator teres; S, m. supinator.

* Cases where the origin of the nerves could not be totally observed (less than 16) due to damage during previous dissections, and thus in the column of percentage, the number of nerves is specified.

The ventral branch of the fifth cervical spinal nerve (C5) extends laterally, branching into two branches, one that passes between the omotransversarius (OT) and the cleidocephalicus muscles at the level of the prescapular region to innervate the cranial aspect of the shoulder. The other branch perforates the m. cleidocephalicus pars cervicalis (CCc) to innervate the craniolateral aspect of the shoulder. In two limbs (FcS3 bilaterally), C5 formed a branch to the origin of the cleidobrachial (*N. cleidobrachialis* ‐ClBN‐) and suprascapular (*N. suprascapularis* ‐SSN‐) nerves (Tables 3 and 4). The cleidobrachial nerve has been named “*N. supraclavicularis*” by other authors in *F. catus* (Pérez & König, 2022; Roos & Vollmerhaus, 2005), and in other felids, *N. brachiocephalicus* (Grzeczka et al., 2024; Souza Junior et al., 2022; Souza‐Junior et al., 2018) or branch of the suprascapular nerve to ClB (Barreto‐Mejía et al., 2022) or branch of the suprascapular nerve to m. brachiocephalicus (Hall et al., 2023). However, we adapted the term *n. cleidobrachialis* since the m. brachiocephalicus is also composed of m. cleidocephalicus, and the nerve only innervates the ClB and skin superficial to it. Similar to our findings in *F. catus*, the contribution of C5 is to the ClBN and suprascapular nerve in *Le. geoffroyi* (Souza‐Junior et al., 2018). In *H. yagouaroundi*, it only contributes to the suprascapular nerve (Souza Junior et al., 2022).

The ventral branch of the sixth cervical spinal nerve (C6) forms a branch cranially, from which the cleidobrachialis and suprascapularis nerves originate. It branches caudally into two divisions: dorsal and ventral. The dorsal division forms the subscapular (*Nn. subscapulares* ‐SbNn‐) and (*N. axillaris* ‐AxN‐), and sometimes contributes to the origin of the radial nerve (*N. radialis* ‐RN‐). The ventral division forms the musculocutaneus nerve (*N. musculocutaneus* ‐McN‐) and sometimes contributes to the origin of cranial pectoral nerves (*Nn. pectorales craniales* ‐PCrN‐) (Table 4).

The ventral branch of the seventh cervical spinal nerve (C7) also forms two divisions (dorsal and ventral). The dorsal division forms the axillary, radial, and thoracodorsal (*N. thoracodorsalis* ‐TDN‐) nerves, the ventral division forms the musculocutaneus, cranial pectoral, and long thoracic (*N. thoracicus longus* ‐TLoN‐) nerves, and two communicating branches, one to the caudal pectoral nerves (*Nn. pectorales caudales* ‐PCdN‐) named *ansa pectoralis* and the other to the median nerve (*N. medianus* ‐MN‐) named branch of C7 to the median nerve (C7MN). The ansa pectoralis passes distal to the origin of the lateral thoracic artery (*A. thoracica lateralis* ‐TLaA‐) to reach the caudal pectoral nerves (Supplementary material 4.10). The branch of C7 to the median nerve (C7MN) extends caudally and parallel to the musculocutaneus nerve (joined by fascia perineural) and distally separates and communicates with the median nerve medial to the brachial artery (*A. brachialis* ‐BA‐) at the level of TMaj. As anatomical variants, the ansa pectoralis can originate from the branch of C7 to the median nerve, cranial pectoral nerves, or caudal pectoral nerves (Table 4). In two limbs (FcS9 bilaterally), the ansa pectoralis originated from the branch of C8 that gave rise to caudal pectoral nerves and after communicating with the cranial pectoral nerves (Supplementary material 4.11). In two limbs (FcS2 bilaterally), the branch of C7 to the median nerve had two communications, one communicating branch with C8 and another with the median nerve. The first communication was at the axillary level (forming an *ansa axillaris* ‐aa‐, Figure 10), and the distal communication was at the brachial level (medial to the TMaj) (Figure 10, Supplementary material 4.12). In one limb (FcS4‐RTL), the branch of C7 to the median nerve sent a communicating branch to McN. In one limb (FcS8‐RTL), the branch of C7 to the median nerve sent a branch to ansa pectoralis.

The ventral branch of the eighth cervical spinal nerve (C8) forms a dorsal division, from which the radial and thoracodorsal nerves originate (Figure 11), and in one limb, the long thoracic nerve. The ventral division forms a common branch to the lateral thoracic and caudal pectoral nerves and another branch to median and ulnar (*N. ulnaris* ‐UN‐) nerves. The ventral branch of the first thoracic spinal nerve (T1) forms a dorsal division to contribute to the origin of RN and a ventral division that gives rise to the median, ulnar, and caudal cutaneous antebrachial nerve (*N. cutaneus antebrachii caudalis* ‐CACdN‐). In one limb (FcS4‐RTL), a very small branch of the second thoracic spinal nerve (T2) communicated with T1 and only contributed to the origin of the ulnar nerve (Figure 12). Pérez and König (2022) also reported this occasional contribution to the ulnar nerve.

#### 3.5.2 Distribution of brachial plexus nerves

The cleidobrachialis nerve (ClBN) extends cranially to the distal extreme of SS belly among the superficial cervical vessels and dorsal to the clavicle, reaching the clavicular intersection and directing distally to the brachium (Figures 1, 10, and 11). It sends a branch to m. cleidobrachialis (ClB), perforates this muscle and extends superficially to ClB into the cranial and medial surfaces of the brachium skin. This distribution is similar to that previously described by Pérez and König (2022). In *Caracal caracal*, it only innervates the skin of shoulder and neck (Grzeczka et al., 2024), whereas in other felids, it only innervates the ClB (Barreto‐Mejía et al., 2022; Souza Junior et al., 2022; Souza‐Junior et al., 2018) or m. brachiocephalicus (Hall et al., 2023). The new specimens of the present study (FcS7‐LTL, FcS8, and FcS10) had the same innervation reported in extrinsic thoracic limb muscles (Vélez‐García et al., 2024). However, the PCdN also innervated the m. pectoralis transversus (PTr) in one limb (FcS7‐LTL) (Supplementary material 4.13).

The suprascapular nerve (SSN) extends between the SS and Sb with the suprascapular vessels at the level of the scapular notch or incisura scapulae (Figures 1, 10, and 11), passing laterally to the scapular neck and branching to the SS, IS, and shoulder joint capsule (ShJC) (Figure 1).

The subscapular nerves (SbNn) are two branches that innervate Sb, which are named cranial and caudal subscapular nerves in this study (SbCrN and SbCdN, respectively). The cranial subscapular nerve (SbCrN) innervated the first, second, and third bellies of Sb. The caudal subscapular nerve (SbCdN) innervates the third, fourth, and fifth bellies (or sixth bellies) of Sb (Figures 10 and 11).

The axillary nerve (AxN) forms several branches. The first cranial branch extends to Sb, and the caudal branch bifurcates to TMaj and the most caudal belly of Sb (fifth or sixth belly) (Figures 10 and 11). The main trunk extends laterally with the caudal circumflex humeral vessels between Sb and TMaj. In this space, it sends branches to the ShJC (AxN^ShJC^) and TMin (AxN™^in^). The nerve continues laterally between TMin and TBLo and sends branches to Ds, Da, and ClB. One branch extends deep and parallel to Ds, reaching the cranial and lateral aspects of the brachium skin, which corresponds to the cranial lateral brachial cutaneous nerve (*N. cutaneus brachii lateralis cranialis* ‐AxN^CrLBC^‐) (Figure 1).

The musculocutaneus nerve (McN) first gives rise to a branch to CB (McN^CB^) just distal to its origin (C6‐C7) (Figure 11), although in several limbs, this branch originated from other nerves (Table 4). The second branch of the musculocutaneus corresponds to the proximal muscular branch (*ramus muscularis proximalis* ‐McN^pmb^‐), which innervates the BB at its proximal part (Figure 10). The musculocutaneus continues distally to the origin of TBm, between the belly of BB and the shaft of humerus (Figures 3 and 10). The third branch corresponds to the medial antebrachial cutaneous nerve (*n. cutaneus antebrachii medialis* –McN^MAC^‐), which passes between BB (medially) and the distal third of the shaft of humerus and more distally between BB (medially) and B and ClB (laterally). The fourth branch corresponds to the distal muscular branch (*ramus muscularis distalis* ‐McN^dmb^‐), which originates just distal to the medial antebrachial cutaneous nerve and innervates the B. The fifth branch extends medially to B until the elbow joint capsule (capsular branch ‐McN^EJC^‐). We observed several anatomical variants in the origin of the branch to CB and the formation of other branches (Table 4). In one limb (FcS2‐LTL), the branch to CB originated from C7 (ap) and a branch of C8. In two limbs (FcS4‐RTL and FcS7‐LTL), the branch to CB originated from musculocutaneus and a branch of C8. In one limb (FcS5‐LTL), the branch to CB originated only from C7 (Figure 12). In one limb (FcS9‐RTL), the branch to CB originated from C7MN and ap (C8), and in another limb (FcS9‐RTL), it originated only from ap (C8) (Supplementary material 4.14). In one limb (FcS3‐LTL), the musculocutaneus gave rise to two branches to CB (two McN^CB^, Supplementary material 4.3). In one limb (FcS5‐RTL), the proximal muscular branch of musculocutaneus nerve sent another small branch to the proximal fourth of BB belly. In one limb (FcS3‐LTL), distal to the proximal muscular branch of musculocutaneus nerve, the musculocutaneus nerve gave rise to a branch to the CBl (McN^CBl^) (Figure 12). In one limb (FcS6‐RTL), the proximal muscular branch of musculocutaneus nerve and another branch that originated directly from the musculocutaneus nerve (distal to the origin of branch to CB) innervated the CBl (Figures 6 and 12). In one limb (FcS7‐LTL), a branch that originated between the branch to CB and proximal muscular branch of musculocutaneus nerve innervated the CBl. In four limbs (FcS1‐LTL, FcS2‐RTL, and FcS5 bilaterally), the musculocutaneus nerve gave rise to a small branch at the middle third of the brachium to BB (McN^BB^, Supplementary material 4.15). In one limb (FcS2‐RTL), that branch (McN^BB^) supplied the belly of BB together with a variant bicipital artery that originated from the brachial artery (Supplementary material 4.4). In two limbs (FcS1‐LTL and FcS3‐RTL), the musculocutaneus nerve formed another branch adjacent to the elbow capsular branch (McN^EJC^) that corresponds to the communicating branch with the median nerve (*ramus communicans* cum *n. mediano* ‐McN^cbMN^‐) inside the supracondylar foramen. In two limbs (FcS3‐RTL and FcS5‐LTL), the musculocutaneus nerve gave rise to another branch that extends to the humerus together with the humeral nutrient artery (*a. nutricia humeri*) (Figure 12).

The radial nerve (RN) results in three divisions: lateral, intermediate, and medial. The lateral division sends branches to TBLo and TFA (Figure 10). The intermediate division sends branches to TBa, TBLa, AL, and TBm. The medial division gives rise to the superficial (*ramus superficialis* ‐RN^sb^‐) and deep (*ramus profundus* ‐RN^db^‐) branches for the craniolateral aspect of the antebrachium (Figure 1). The RN^db^ innervates all craniolateral muscles of the antebrachium, and in one limb (FcS4‐RTL), it innervates the B (Supplementary material 4.5).

The median nerve (MN) extends medially to the axillary artery and proximally caudal to the brachial artery, passing toward the medial aspect of the brachial artery at the level of the TMaj, where it receives the branch of C7. The PAn covered the median nerve at the brachium level. The median nerve continued craniomedial to the brachial artery, passing together through the supracondylar foramen (Figures 9 and 10). It innervates most caudomedial antebrachial muscles, except the m. flexor carpi ulnaris (FCU) and flexor digitorum brevis (FDB).

The ulnar nerve (UN) extends between TFA and TBm at the brachial level where it forms an impression on the medial surface of TBm (Figure 5). It sends a branch to AM proximal to this and crosses deeply to this muscle, where it forms another branch to AM (Supplementary material 4.9) and others to EJC (capsular branch), and the caput ulnare of the m. flexor carpi ulnaris (FCUu). Distally to AM (at the antebrachial level), the UN innervates the FCUu, caput humerale of FCU, caput ulnare, and humerale superficiale mediale of m. flexor digitorum profundus (FDPu and FPhsm). The main anatomical variants were observed in the branching to AM. In one limb (FcS3‐LTL), the branch proximal to the AM divided into two branches to the proximal and distal aspects of AM (in this limb, the UN did not form a branch deep to the AM) (Figure 9). In two limbs (FcS4‐LTL and FcS5‐RTL), the UN formed three branches to AM: one proximal to AM and two deep to AM (Supplementary material 4.9). In two limbs (FcS4‐LTL and FcS6‐RTL), the capsular branch originated from the distal branch to AM.

The caudal antebrachial cutaneous nerve (CACdN) extends medially to the ulnar nerve and caudally to the median nerve. The caudal antebrachial cutaneous nerve passes toward the superficial plane at the middle level of the brachium between the TFA and PAb. It does not innervate muscles. According to the NAV (International Committee on Veterinary Gross Anatomical Nomenclature, 2017) and other authors who have published on the anatomy of felids, this nerve is a branch of the UN (Barreto‐Mejía et al., 2022; Grzeczka et al., 2024; Hakkı Nur et al., 2020). However, we found it as a nerve with an independent origin from the first thoracic spinal nerve (T1), as observed in previous studies of the same species (Ghoshal & Magilton, 1972; Pérez & König, 2022; Roos & Vollmerhaus, 2005) and *P. uncia* (Hall et al., 2023). Other authors did not report this caudal antebrachial cutaneous nerve in their felid species (Souza Junior et al., 2022; Souza‐Junior et al., 2018; Takcı & Arı, 2025).

## 4 DISCUSSION

We found that the axillary nerve (AxN) is the main innervation of the m. cleidobrachialis (ClB) in *F. catus*, as observed in previous studies of the same species (Pérez & König, 2022; Roos & Vollmerhaus, 2005; Vélez‐García et al., 2024), but also in *Pu. concolor* (Barreto‐Mejía et al., 2022) and *C. caracal* (Grzeczka et al., 2024). However, due to the complementary or unique innervation by the n. cleidobrachialis (ClBN) in felids, a previous study inferred that the ClB could have derived not only from the deltoid complex but also from the supracoracoid group (Vélez‐García et al., 2024). This latter derivation is supported by the splitting pattern found during embryological development in mammals by Smith‐Paredes et al. (2022), who reported that the homologous part of ClB (m. delotideus pars clavicularis ‐Dc‐) splits from the supracoracoid group together with *m. pectoralis descendens* (PD) (“superficial portion of the m. pectoralis major” of Smith‐Paredes et al., 2022), SS, and IS. This muscle derivation also explains the common origin of both cleidobrachial (ClBN) and suprascapular (SSN) nerves, and the parallel arrangement of ClB and the superficial part of the *m. pectoralis descendens* (*m. pectoantebrachialis* ‐PAn‐) until their insertions in carnivorans (Vélez‐García et al., 2024; Vélez‐García & Miglino, 2023). Thus, although previous studies in Carnivora inferred that ClB was the clavicular part of D (“*m. deltoideus pars clavicularis*” of NAV) (Diogo et al., 2012; Vélez‐García et al., 2024; Vélez‐García & Miglino, 2023), it more likely evolved from the supracoracoideus group, similar to other representative mammals (Smith‐Paredes et al., 2022) (Table 5), whereas Ds and Da derived from the deltoid complex together with TMin (Smith‐Paredes et al., 2022) (Table 6). Furthermore, functionally the ClB is an antagonist muscle to the deltoid complex muscles (Ds, Da, and TMin) since it is a shoulder extensor, and also an elbow flexor due to its insertion onto the ulna in felids (Vélez‐García et al., 2024). We observed elbow flexor function when we simulated contraction in one limb without formalin fixation (Supplementary material 5). These are reasons to eliminate the name *m. deltoideus pars clavicularis* and only conserve the name *m. cleidobrachialis* in a new edition of the NAV, along with the term *n. cleidobrachialis*. We advocate that function, embryological derivations, and evolution should be considered in future discussions on veterinary gross anatomical nomenclature.

**TABLE 5:** Homology and derivation inferences of the ventral musculature of the shoulder and brachium muscles of Felidae from Amniota.

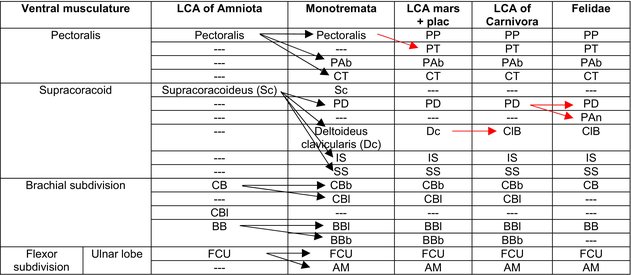

*Note*: The first two columns and their divisions are based on the embryological study of Smith‐Paredes et al. (2022). The next columns are based on those reported in mammals by several authors (Diogo et al., 2018; Gambaryan et al., 2015; Vélez‐García et al., 2024). Black arrows indicate the derivation hypotheses based on Smith‐Paredes et al. (2022). Red arrows indicate the hypotheses of the present study.

**TABLE 6:** Homology and derivation inferences of the dorsal musculature of the shoulder and brachium muscles of Felidae from Amniota.

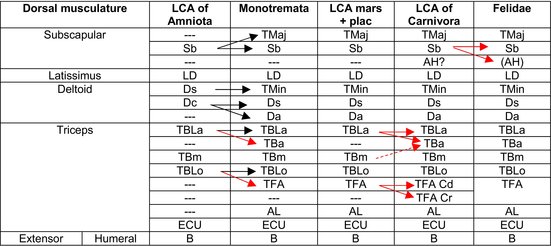

*Note*: The first two columns and their divisions are based on the embryological study of Smith‐Paredes et al. (2022). The next columns are based on those reported in mammals by several authors (Diogo et al., 2018; Gambaryan et al., 2015; Vélez‐García et al., 2024). Black arrows indicate the derivation hypotheses based on Smith‐Paredes et al. (2022). Red arrows indicate the hypotheses of the present study.

Previously, the pectoral muscles of mammals were considered to be derived only from the pectoral division (*m. pectoralis*) of Amniota (Diogo et al., 2018). However, Smith‐Paredes et al. (2022) reported that, in mammals, the *m. pectoralis profundus* (PP‐) (“*m. pectoralis minor*” of Smith‐Paredes et al., 2022), *m. pectoralis transversus* (PT‐) (“deep portion of the m. pectoralis major” of Smith‐Paredes et al., 2022), and *m. cutaneus trunci* (CT‐) (“*m. panniculus carnosus*” of Smith‐Paredes et al., 2022) are the only muscles derived from the pectoral division, whereas the PD is derived from the supracoracoid division. Therefore, although previous studies inferred that PD and PT are derived from the pectoral division in Carnivora (Caniformia and Feliformia) (Vélez‐García et al., 2024; Vélez‐García & Miglino, 2023), these muscles are actually derived from the supracoracoideus and pectoralis muscles, respectively, similar as observed in other mammals (Smith‐Paredes et al., 2022) (Table 6). This developmental derivation of PT could explain the communication of the cranial (PCrN) and caudal pectoral (PCdN) nerves through an ansa pectoralis (ap) in felids. The variant innervation of the caudal pectoral nerve (PCdN) to PT in one limb of *F. catus* (FcS7‐LTL) can be explained by the same developmental origin of the PP and PT. Thus, the presence of the ansa pectoralis could be a plesiomorphy of the brachial plexus in Carnivora since both arrangements are normally present in several representative caniforms (Davis, 1964; Enciso‐García & Vélez‐García, 2022; Vélez‐García, de Carvalho Barros, & Miglino, 2023) and as an anatomical variation in the domestic dog (*Canis lupus familiaris*) (Kamali, 2024). Furthermore, the ansa pectoralis is also present in some noncarnivoran scrotifers, such as the pangolin (*Manis pentadactyla pentadactyla*) (Kawashima et al., 2015) and a cetartiodactyl, such as the goat (*Capra aegagrus hircus*) (Kamali, 2025a). In addition, complementary innervation of caudal pectoral nerves to m. pectoralis transversus has been reported in the typical pattern of caniforms of the family Procyonidae (Enciso‐García & Vélez‐García, 2022; Vélez‐García, de Carvalho Barros, & Miglino, 2023).

Previously, a study on the innervation patterns to LD, TMaj, and Sb in representative species of Amniota inferred that TMaj and Sb of mammals could be derived from the LD of Reptilia (Koizumi, 2022). However, the embryonic muscle splitting pattern revealed that there is no homologous TMaj between Reptilia and Mammalia. Thus, in mammals, the TMaj is derived from the subscapular group (Smith‐Paredes et al., 2022). This coincides with the common branch of the axillary nerve (AxN) to both muscles (TMaj and Sb) and the independent innervation to LD by the thoracodorsal nerve (TDN) in *F. catus* (Table 6).

Specific veterinary anatomical references in *F. catus* do not report innervation to the AH (Hudson & Hamilton, 2010; Pérez & König, 2022; Roos & Vollmerhaus, 2005). However, Liebich et al. (2020) report that the axillary nerve innervates the AH in domestic mammals that have it, such as *F. catus*. This innervation agrees with the caniform *Nasua nasua* (Vélez García et al., 2025), in which the topology is similar to that present in other carnivorans, such as the caniform *Ursus maritimus* (Polar bear) (Kjaersgaard, 1974) and the feliforms *Pa. leo* (Barone, 1967) and *F. catus* (Pérez & König, 2022). Vélez García et al. (2025) inferred that the AH could derive from Sb based on the topology medial to the TBLo tendon and the innervation by a branch of the axillary nerve that first extends to Sb. Therefore, although we did not observe innervation in the unique AH present in our sample, it conserved the topology described previously (Pérez & König, 2022), and its evolutionary derivation is therefore also most likely from the Sb (Table 6). The AH is present in other noncarnivoran scrotifers, such as perissodactyls and some terrestrial cetartiodactyls (Kjaersgaard, 1974). According to a histological study in a domestic horse (*Equus ferus caballus*), the AH is a receptor organ of the shoulder joint capsule due to its high content of muscle fiber spindles (Lalatta‐Costerbosa et al., 1992). Based on the work of Lalatta‐Costerbosa et al. (1992), the AH is an atavistic muscle that could have helped to stabilize the shoulder joint capsule by sending proprioceptive signals to the central nervous system to produce a response through Sb activation to stabilize the shoulder in carnivoran ancestors.

The CB and BB share the same derivation from the cranial division of the brachium in mammals (Smith‐Paredes et al., 2022), and the musculocutaneus nerve innervates both muscles in all felids. However, in several forelimbs, we observed that the motor branch to the CB originated from other nerves, such as the ansa pectoralis or the branch of C7 to the median nerve (MNC7). Based on the evolutionary history of the brachial plexus in scrotifers, the musculocutaneus and median nerves conserved a common origin; thus, there is an ansa axillaris that connects both nerves in terrestrial cetartiodactyls (Backus et al., 2016; Kamali, 2025a), perissodactyls (Backus et al., 2016), pholidota (Kawashima et al., 2015), and some arctoids of the order Carnivora (Davis, 1964; Vélez‐García, de Carvalho Barros, & Miglino, 2023). In this latter order, even in one species that normally does not have an ansa axillaris, such as Ca. *lupus familiaris*, it was found as an anatomical variant in one specimen (Kamali, 2022), similar to our findings in one *F. catus* specimen. In the first divergent mammals, such as monotremes (platypus and echidnas), the branch to CB (McN^CB^) also has several origins related to the formation of the musculocutaneus and pectoral nerves (Koizumi & Sakai, 1997). Thus, the variable origin of the branch to CB and the variable presence of ansa axillaris in extant felids and canids would indicate that the last common ancestor of Carnivora could have had an ansa axillaris.

The long portion of the CB corresponds to *m. coracobrachialis longus* (CBl), which is reported within the suborder Feliformia in a representative species of the family Nandiniidae, the African palm‐civet (*Nandinia binotata*) (Carlsson, 1900); two euplerids (family Eupleridae), such as the fossa (*Cryptoprocta ferox*) (Böhmer et al., 2020; Carlsson, 1911) and Eastern falanouc (*Eupleres goudotii*) (Carlsson, 1902); and viverrids of the genus *Genetta* (Genets) (Taylor, 1974). In *Cr. ferox*, Carlsson (1911) noted the CBl as a rudimentary muscle with an insertion proximal to the medial epicondyle and more developed than in *F. catus*. In Caniformia, it is present in several species of arctoids (Vélez‐García, Carrión Blanco, et al., 2023). Therefore, the variable presence of CBl in *F. catus* indicates that it is an atavistic muscle that potentially could be present in the last common ancestor of carnivorans (Table 5). No authors have reported the caput breve BB (BBb) in feliforms, while several arctoids, such as the kinkajou (*Potos flavus*), red panda (*Ailurus fulgens*), and bear species of the genera *Ursus* and *Tremarctus*, have it (Vélez‐García, Carrión Blanco, et al., 2023). Therefore, similar to CBl, the variant and vestigial presence of BBb, as occurred in one *F. catus* in our sample, may be a product of homoplasy. Functionally, the abduction force could be increased in *F. catus* specimens that present a well‐developed CBl. Furthermore, the BB could act as a potential supinator in felids allowing more force in rotational movements. Interestingly, when we simulated the contraction of BB in a limb without formalin fixation, we observed that the muscle also supinates (Supplementary material 6). Thus, the presence of both CBl and BBb could be a pleisomorphy associated with an ancestor with high arboreal and manual abilities based on the type of locomotion of carnivorans that have these muscles.

Prior to the embryological study of Smith‐Paredes et al. (2022), the B was considered to be derived from the same division of BB since both are innervated by the musculocutaneus nerve, are agonist muscles for elbow flexion, and previous embryological studies supported (Diogo et al., 2018). However, with novel marking techniques, Smith‐Paredes et al. (2022) reported that B actually derives from the most cranial subdivision of the triceps division in representative embryos of amniotes (Smith‐Paredes et al., 2022). Therefore, although the musculocutaneus nerve innervates both BB and B, the developmental derivation is distinct in these muscles (Tables 5 and 6), which could explain the variable additional innervation of the B by the radial nerve in several species., Innervation of the B by the musculocutaneus and radial nerves have been observed in one limb of *F. catus* of the present study and have been described in other feliforms, such as *Pa. uncia* (Hall et al., 2023), *N. binotata* (Carlsson, 1900), *Eu. goudotti* (Carlsson, 1902), and the ring‐tailed mongoose (*Galidia elegans*) (Carlsson, 1910). It has also been reported in the caniforms as *Ai. fulgens* (Carlsson, 1925) and the crab‐eating raccoon (*Procyon cancrivorus*) (Vélez‐García, de Carvalho Barros, & Miglino, 2023), and noncarnivoran scrotifers such as the perissodactyl *Eq. ferus caballus* (Barone, 2020; Budras et al., 2009) and the cetartiodactyls as the bovine (*Bos primigenius taurus*) (Budras & Habel, 2011) and goat (*Capra aegagrus hircus*) (Kamali, 2025b). However, the consistent innervation of B by the distal branch of musculocutaneus nerve (McN^dmb^) and its anatomical topology support the establishment of homologies across mammalian species.

The TFA, TB, and AL are derived from the tricipital division of the brachium (Smith‐Paredes et al., 2022), extend the elbow, and are innervated by the radial nerve in all felids. Based on the branching of the radial nerve to the TBLo and TBLa in *F. catus*, we infer that TFA and TBa could be derived from these heads, respectively (Table 6), as was suggested in caniforms (Vélez García et al., 2024). This is also supported by the variable fusions that can be present between TBa and TBLa in the feliform as the striped hyena (*Hyaena hyaena*) (Spoor & Badoux, 1985) and the caniform *Pr. cancrivorus* (Vélez García et al., 2024). Therefore, the presence of TBa (four heads of the TB) is a plesiomorphy in the order Carnivora, which is also present in monotremes (Gambaryan et al., 2015) and several cetartiodactyls (Kamali, 2025b).

Previous anatomical studies on *F. catus* did not report innervation to the AM (Barone, 2020; De Iuliis & Pulerà, 2019; Hakkı Nur et al., 2020; Hudson & Hamilton, 2010; Pérez & König, 2022; Reighard & Jennings, 1901; Sebastiani & Fishbeck, 2005), and other studies likewise failed to describe this innervation in other felids (Hall et al., 2023; Souza Junior et al., 2022; Souza‐Junior et al., 2018; Takcı & Arı, 2025). One study reported a branch of the ulnar nerve to the m. anconeus in *F. catus* (Roos & Vollmerhaus, 2005), and another to a portion of TBm in *F. catus* and *Ly. lynx* (Agduhr, 1915), and other studies to the accessory head of TBm in *F. catus* (Ghoshal & Magilton, 1972; Silva & Sánchez, 2013), *Pa. onca*, and *Pu. concolor* (Silva & Sánchez, 2013). These references actually referred to AM based on our observations in *F. catus* and other felids, such as *Pu. concolor* (Barreto‐Mejía et al., 2022) and *Caracal caracal* (Grzeczka et al., 2024). Unfortunately, several studies did not report nor describe the AM, despite the muscle being in their figures (da da Costa da Silva et al., 2025; Hall et al., 2023; Souza‐Junior et al., 2018; Souza Junior et al., 2022; Takcı & Arı, 2025). Although studies performed by the same coauthors reported the presence of the AM (with a different name) in *Ly. lynx* (Ari, 2024) and *Pa. uncia* (Smith et al., 2021), in subsequent reports of the brachial plexus nerves in both species, they did not describe the innervation of that muscle (Hall et al., 2023; Takcı & Arı, 2025). In *Le. geoffroyi*, Cardozo et al. (2025) confirmed the presence of this muscle, but Souza‐Junior et al. (2018) did not report its innervation. Several species from other feliform families possess an AM, except hyaenids (Spoor & Badoux, 1985; Watson & Young, 1879), viverrids of the genera *Proteles* (Watson, 1882), *Civettictis* (Alfred, 1880; Devis, 1868; Macalister, 1873; Young, 1880), the euplerid *E. goudotii* (Carlsson, 1902), and the nandiniid *N. binotata* (Carlsson, 1900). The AM is embryologically derived from the antebrachium muscle mass together with the FCU and m. palmaris longus in mammals (Smith‐Paredes et al., 2022). Therefore, although the AM is functionally an elbow extensor, it is an independent muscle and not a head of TB in felids.

## 5 CONCLUSIONS

The present study described in detail and reconstructed the muscle maps of the shoulder and brachium muscles in *F. catus*, which should be considered in veterinary procedures and evolutionary studies of thoracic limb muscles in species of the order Carnivora. The homologies of the shoulder and brachium muscles of *Felis catus* could be established based on topology, attachments (origin and insertion), innervation (brachial plexus nerve distribution), and anatomical variants. Unlike other studies, the presence of m. articularis humeri was variable, and the m. anconeus medialis was not a part of the caput mediale of m. triceps brachii. The variable presence of m. articularis humeri, m. coracobrachialis longus, and the vestigial caput breve of m. biceps brachii could be related to atavistic muscles that could be present in a common ancestor of the order Carnivora. Although the present study was limited to anatomical descriptions and evolutionary inferences of muscles and nerves of the shoulder and brachium in *F. catus*, it provides a foundation for future research. Such studies should consider anatomical variants and muscle shape to assess potential functional implications (e.g., increased force due to pennate shapes or the presence of additional muscles) and clinical consequences (e.g., nerve entrapment, as observed in one musculocutaneus nerve). Furthermore, it is necessary to perform specific developmental studies in this species to corroborate patterns of muscle and nerve formation. Such data, when compared across taxa, may provide insights into possible evolutionary pathways.

## AUTHOR CONTRIBUTIONS

J.F.V.G. and M.A.M. contributed to the study conception and design. Material preparation, acquisition and analysis of the data were performed by J.F.V.G. The first draft of the manuscript was written by J.F.V.G. J.F.V.G. and I.V.C. designed the figures and digitized the muscle maps. M.A.M. commented on the posterior versions of the manuscript. All the authors read and approved the final manuscript.

## Supporting information


**Supplementary material 1.** Video of muscle maps in a digitized left scapula of *Felis catus*. Review this video together Figure 2.

**Supplementary material 2.** Video of muscle maps in a digitized left humerus of *Felis catus*. Review this video together Figure 3.

**Supplementary material 3.** Video of muscle maps in digitized and articulated left radius and ulna of *Felis catus*. Review this video together Figure 7.

**Supplementary material 4.** Photographs of anatomical variants.

**Supplementary material 5.** Video of ClB contraction simulation in a limb without formalin fixation. Video taken with IPhone15.

**Supplementary material 6.** Video of BB contraction simulation in a limb without formalin fixation. Video taken with IPhone15.

## Data Availability

The data that supports the findings of this study are available in the supplementary material of this article.
