# Supplementary material for: Updating the forelimb anatomy of the domestic cat (Felis catus, Felidae) based on evolutionary inferences of its muscles and nerves I: Shoulder and brachium
Source: J Anat. 2026 Apr 19;249(5):1138–69. doi: 10.1111/joa.70151 (PMC13398976; doi:10.1111/joa.70151)
Supplement: Supplementary file 1 [file JOA-249-1138-s005.pdf]

4.1

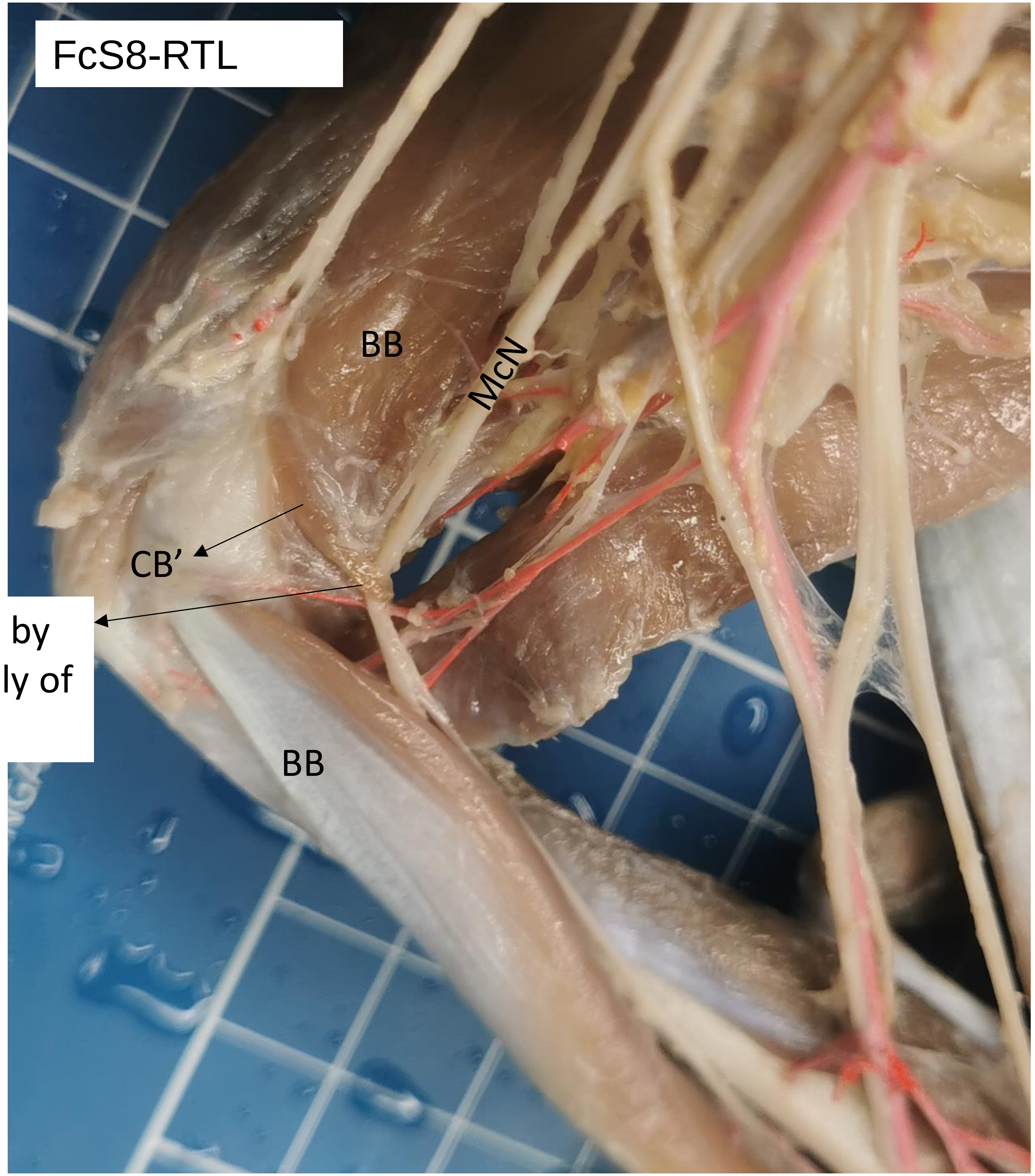

McN entrapment by the accessory belly of CB (CB’)

View without McN

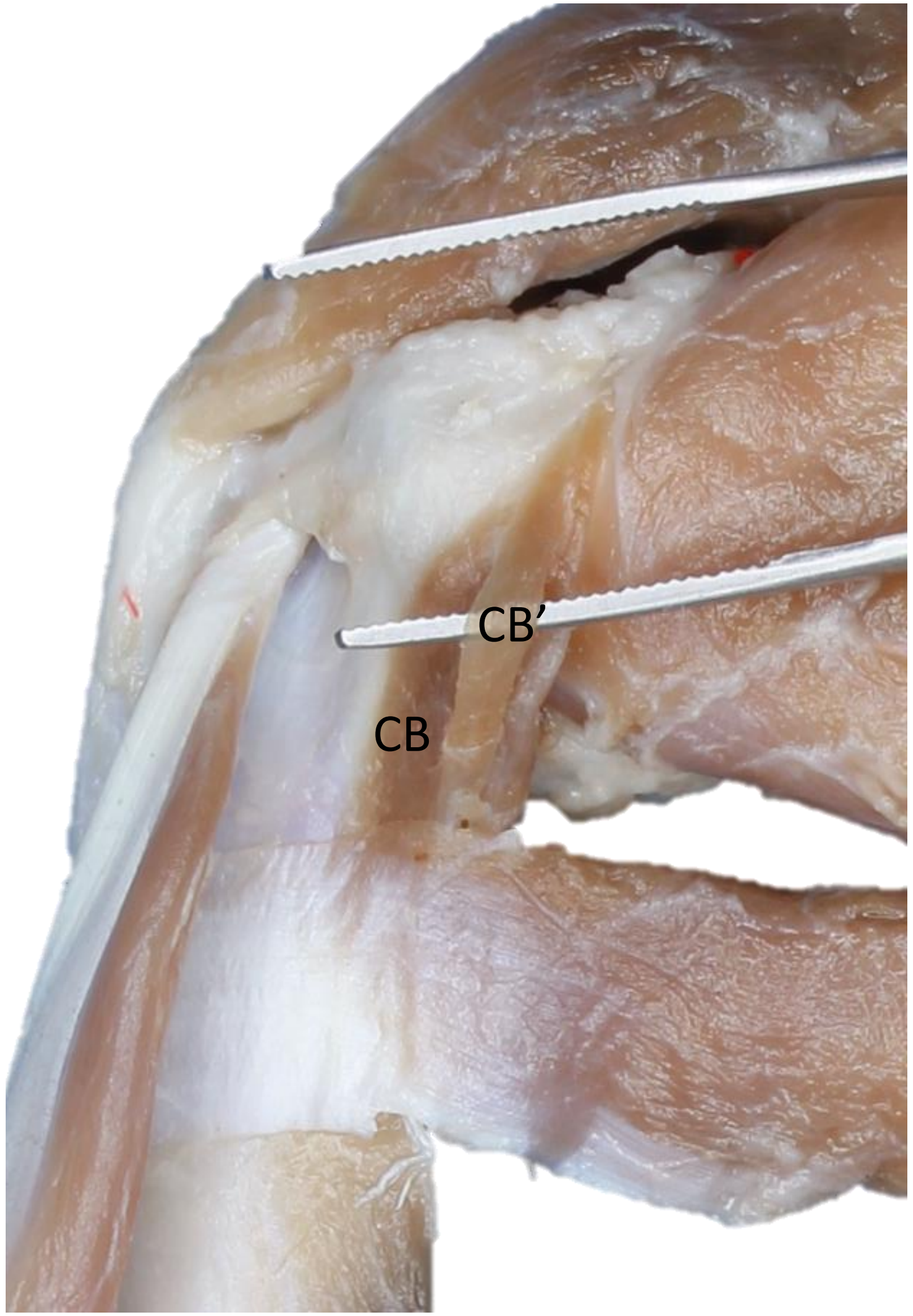

4.2

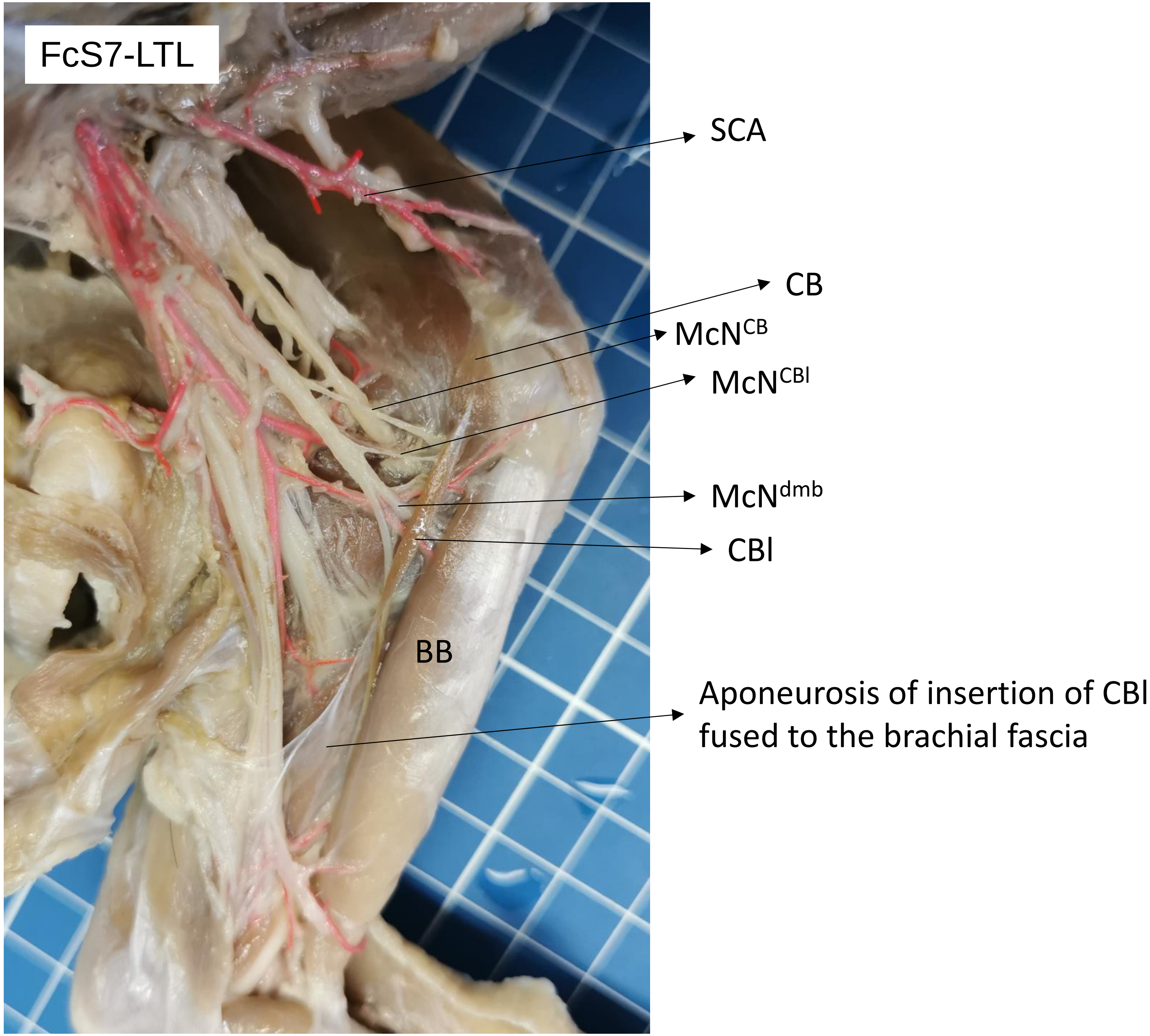
FcS7-LTL
SCA
CB
McN^CB
McN^CBl
McN^dmb
CBl
BB
Aponeurosis of insertion of CBl
fused to the brachial fascia

4.3

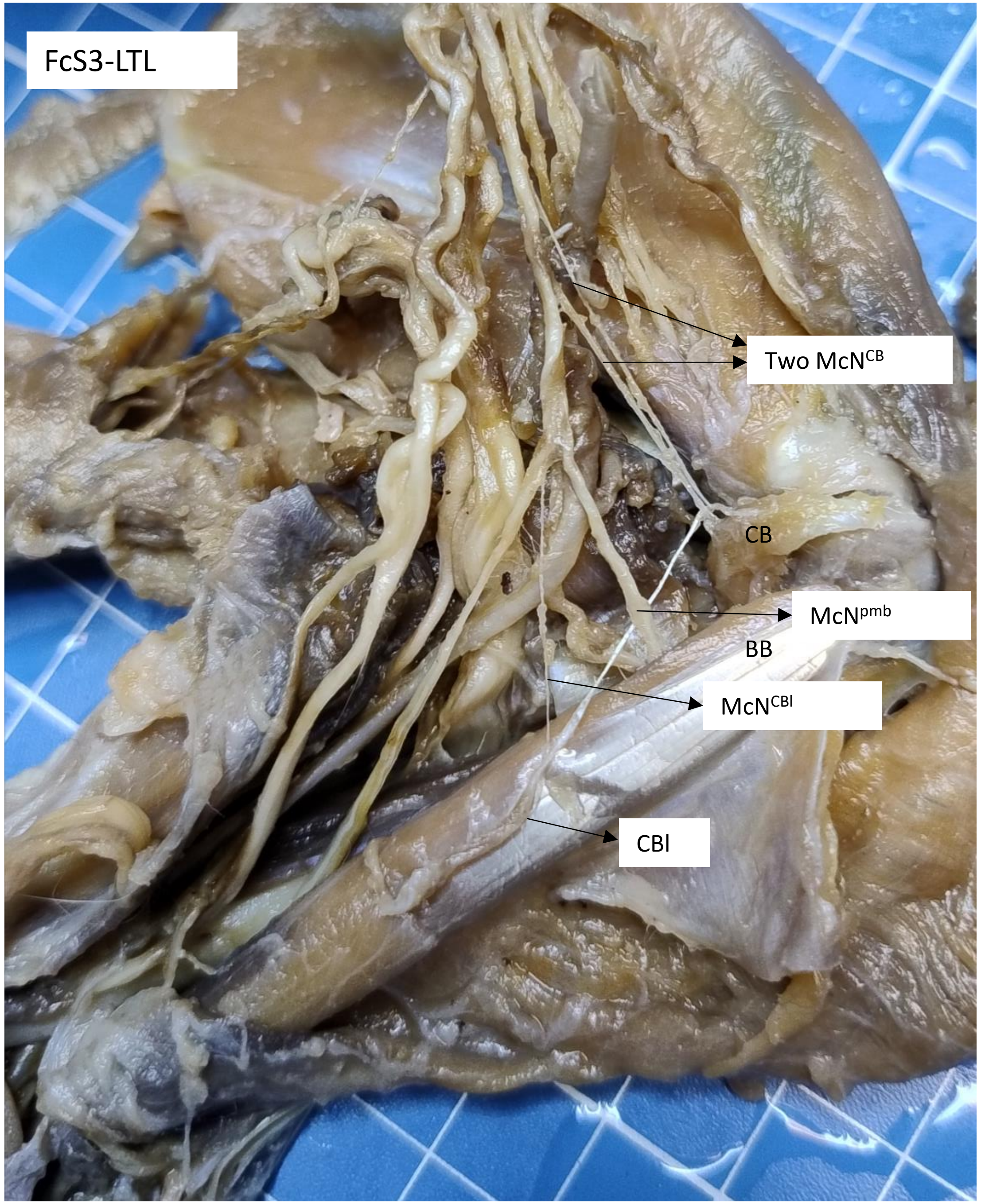

4.4

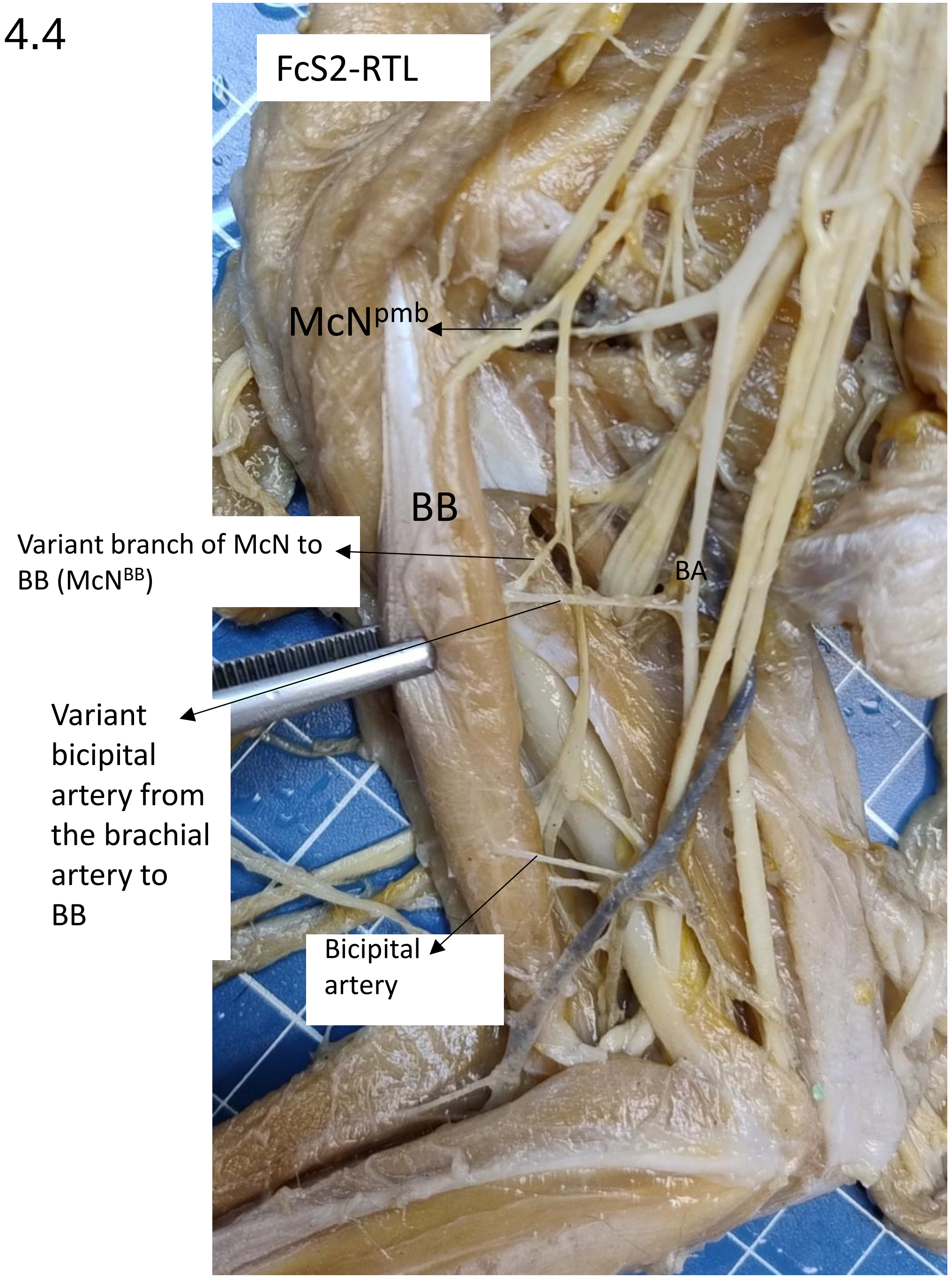
FcS2-RTL
McN^pmb
BB
Variant branch of McN to BB (McN^BB)
BA
Variant bicipital artery from the brachial artery to BB
Bicipital artery

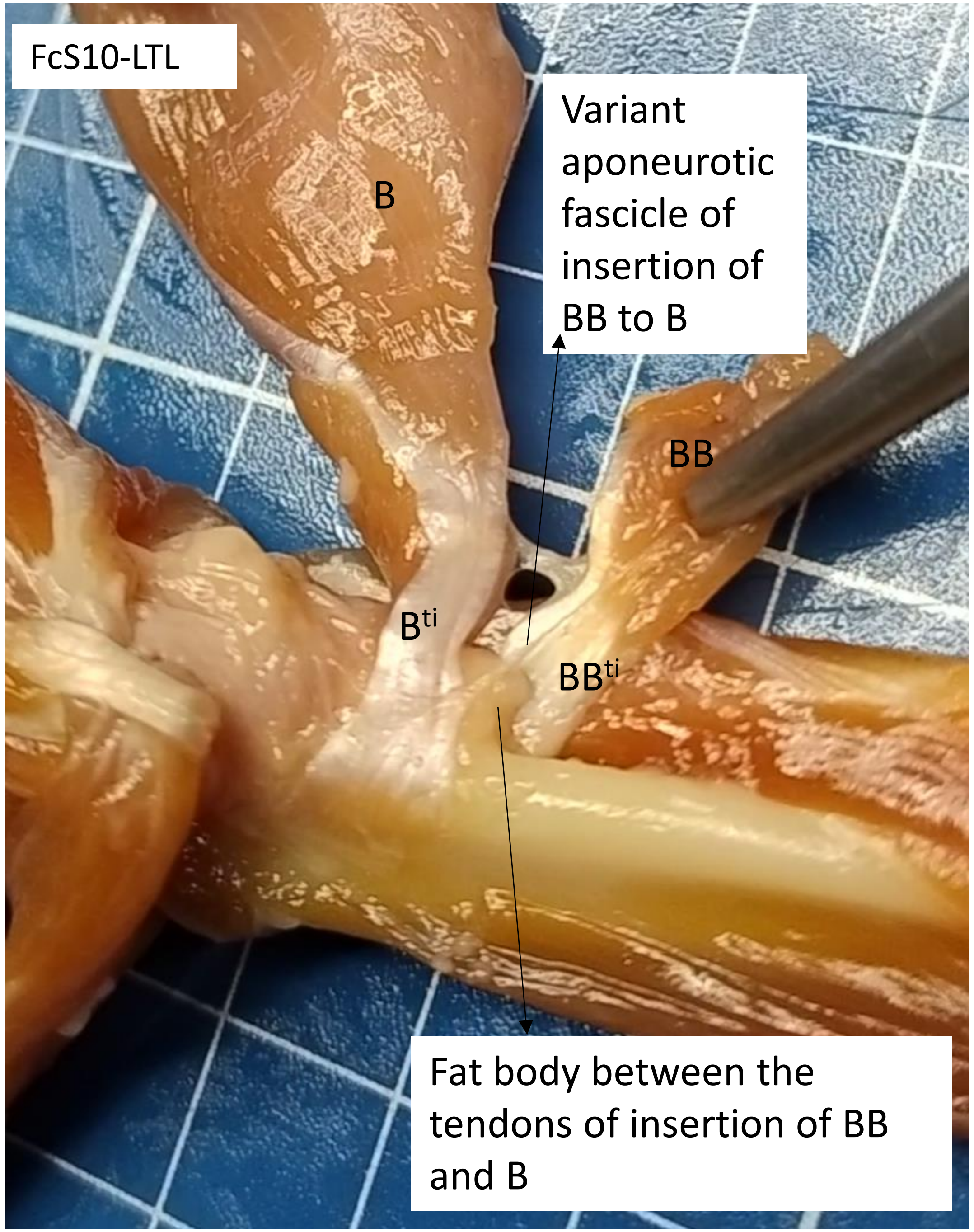
FcS10-LTL
B
Variant aponeurotic fascicle of insertion of BB to B
BB
B^ti
BB^ti
Fat body between the tendons of insertion of BB and B

4.5

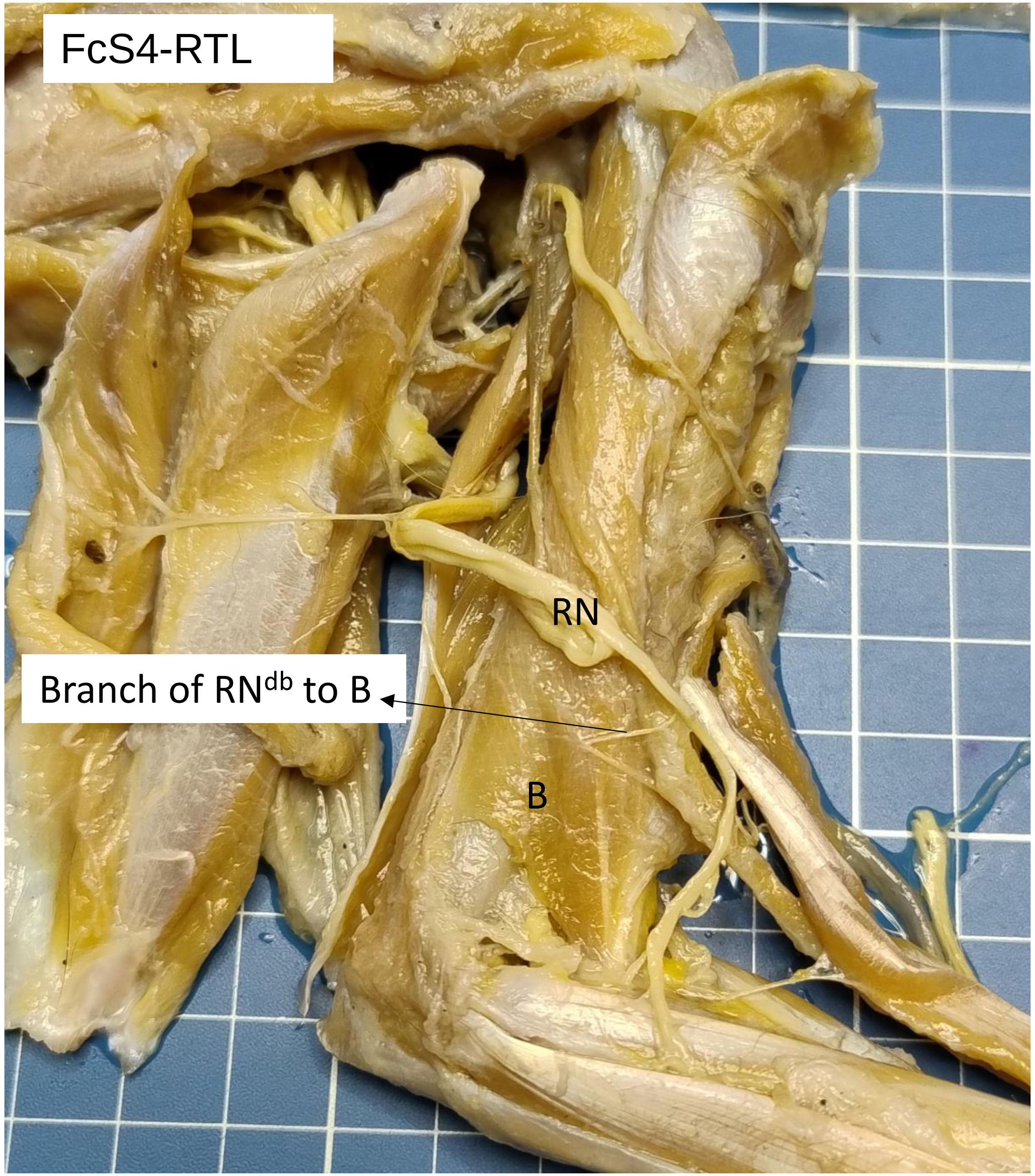

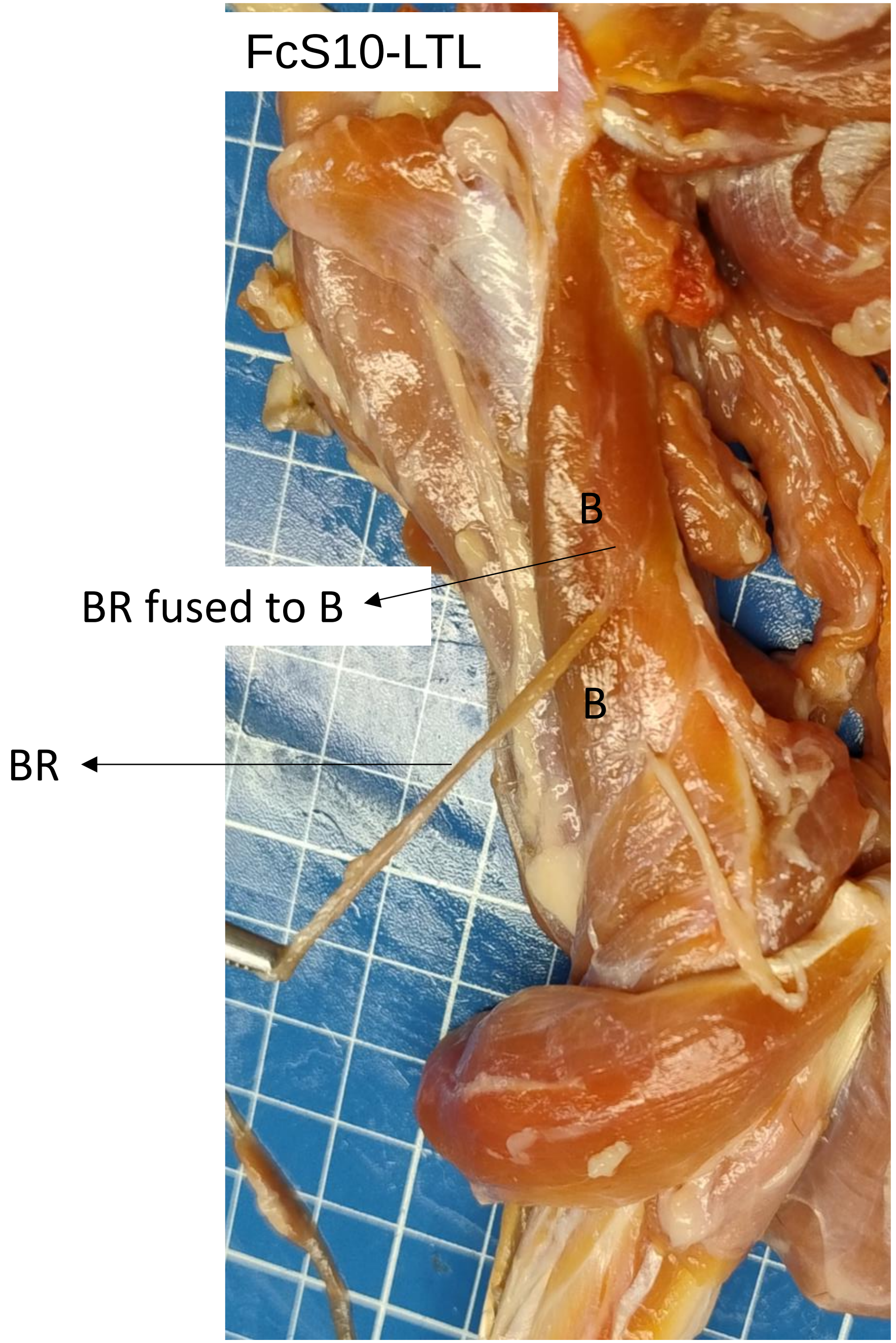

4.6

FcS5-LTL

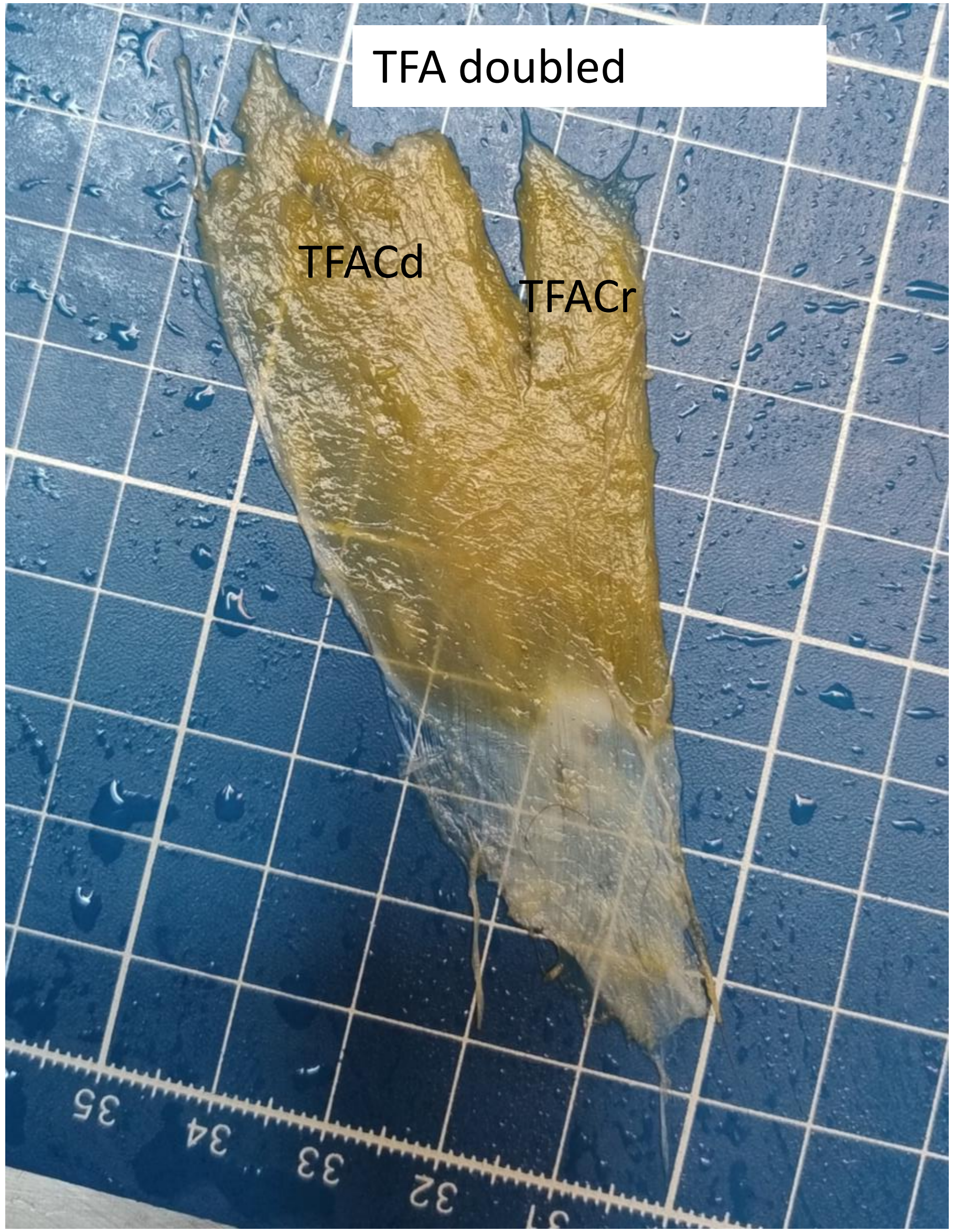

FcS5-LTL

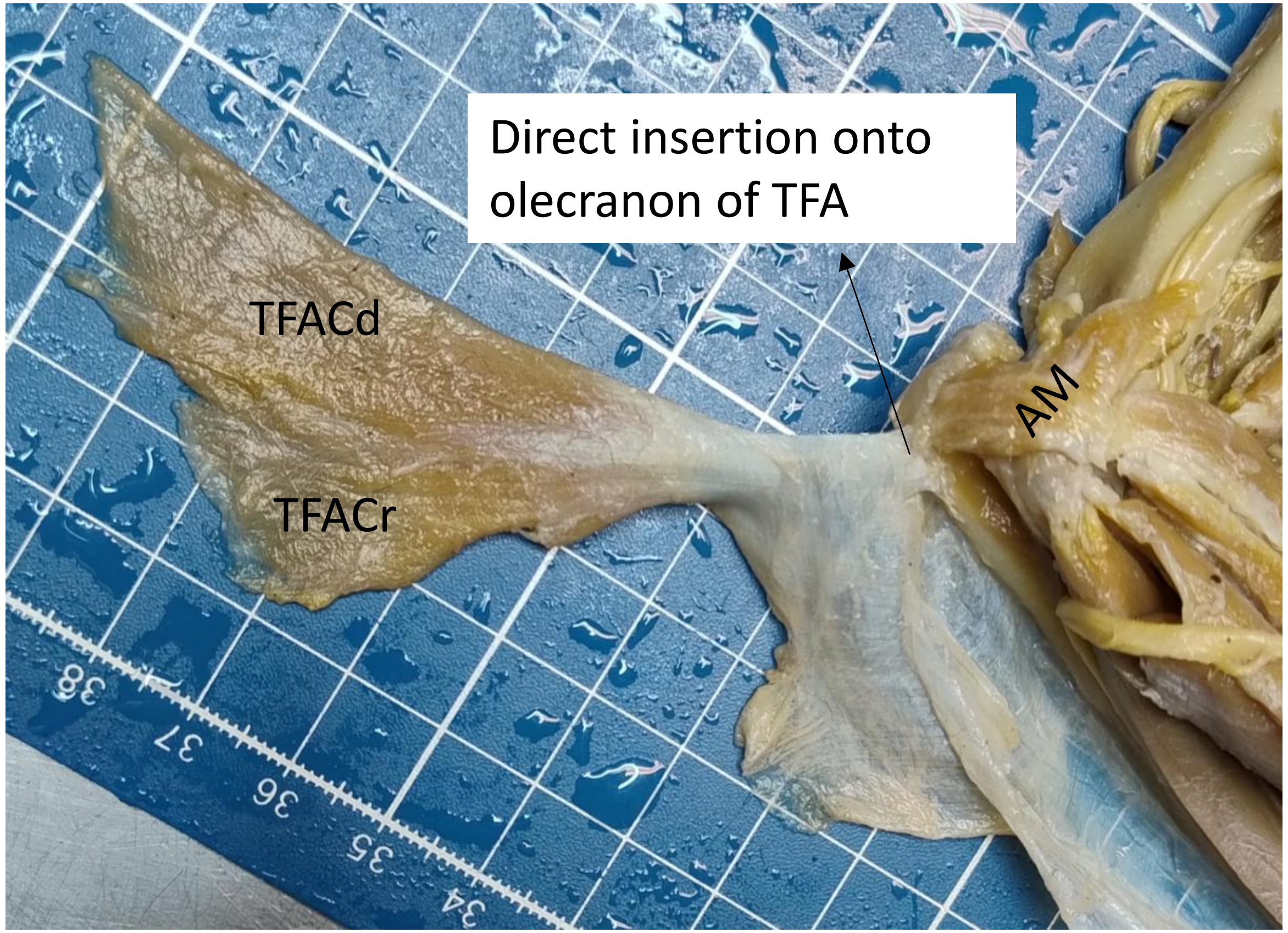

4.7

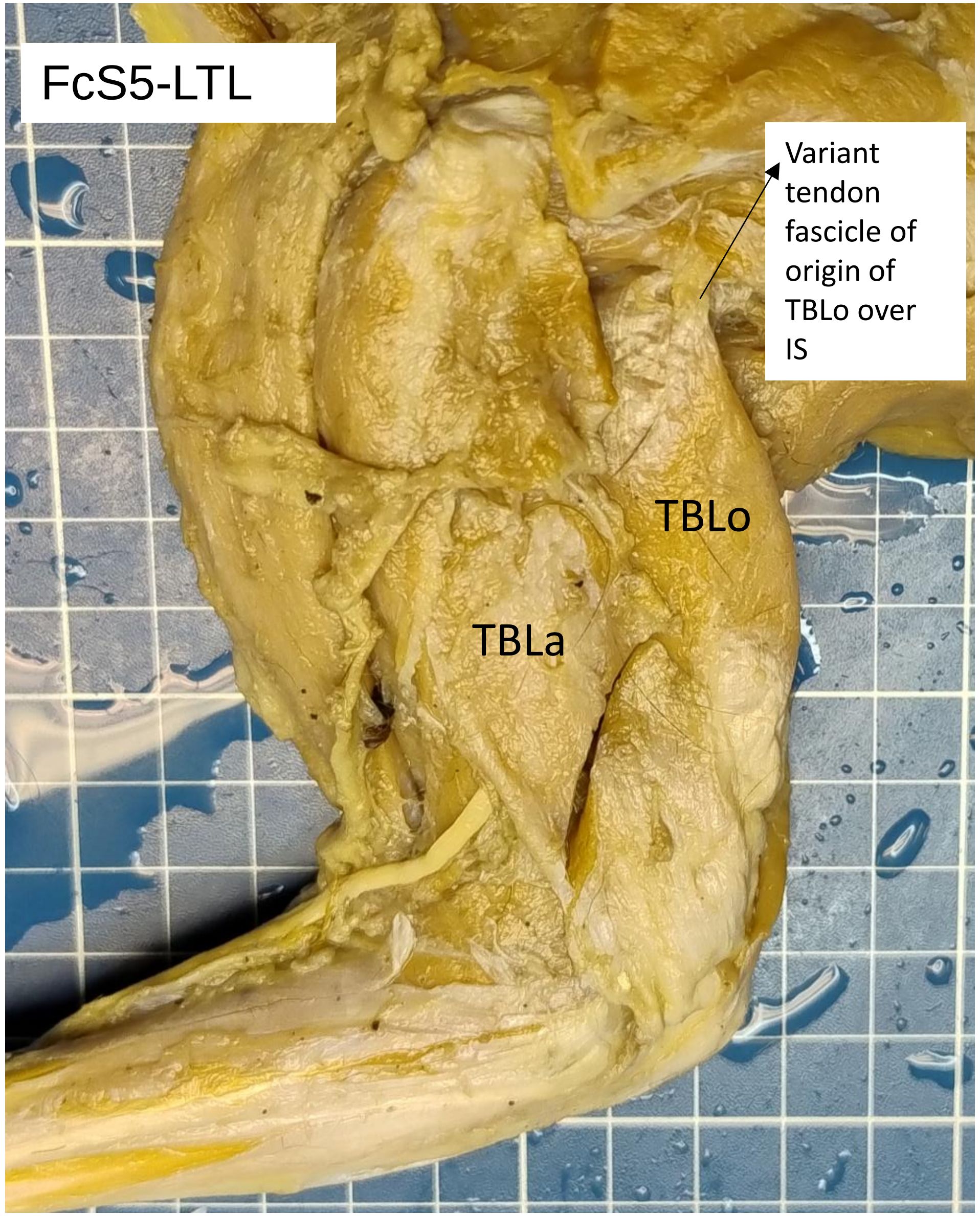
FcS5-LTL
Variant tendon fascicle of origin of TBLo over IS
TBLo
TBLa

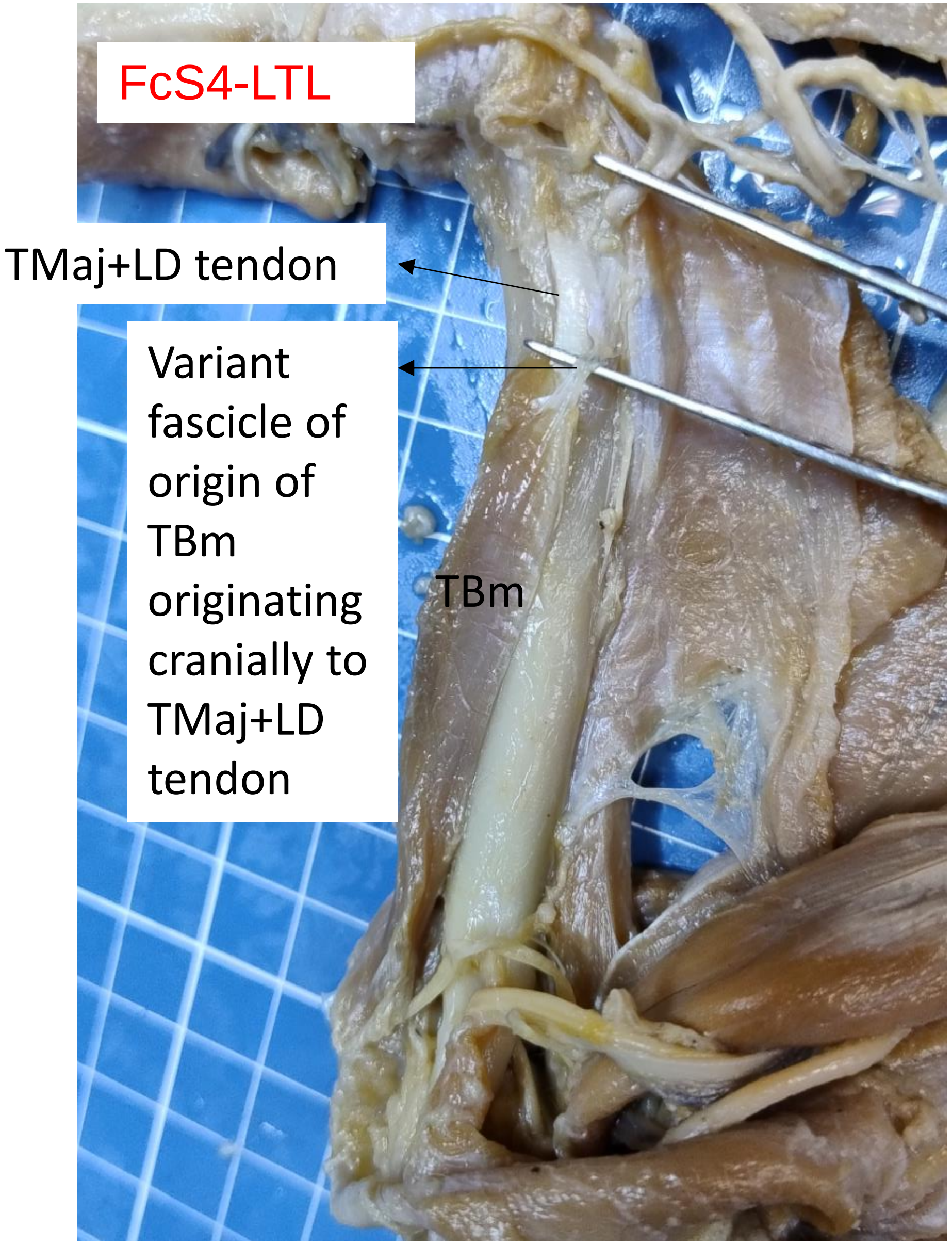
FcS4-LTL
TMaj+LD tendon
Variant fascicle of origin of TBm originating cranially to TMaj+LD tendon
TBm

4.8

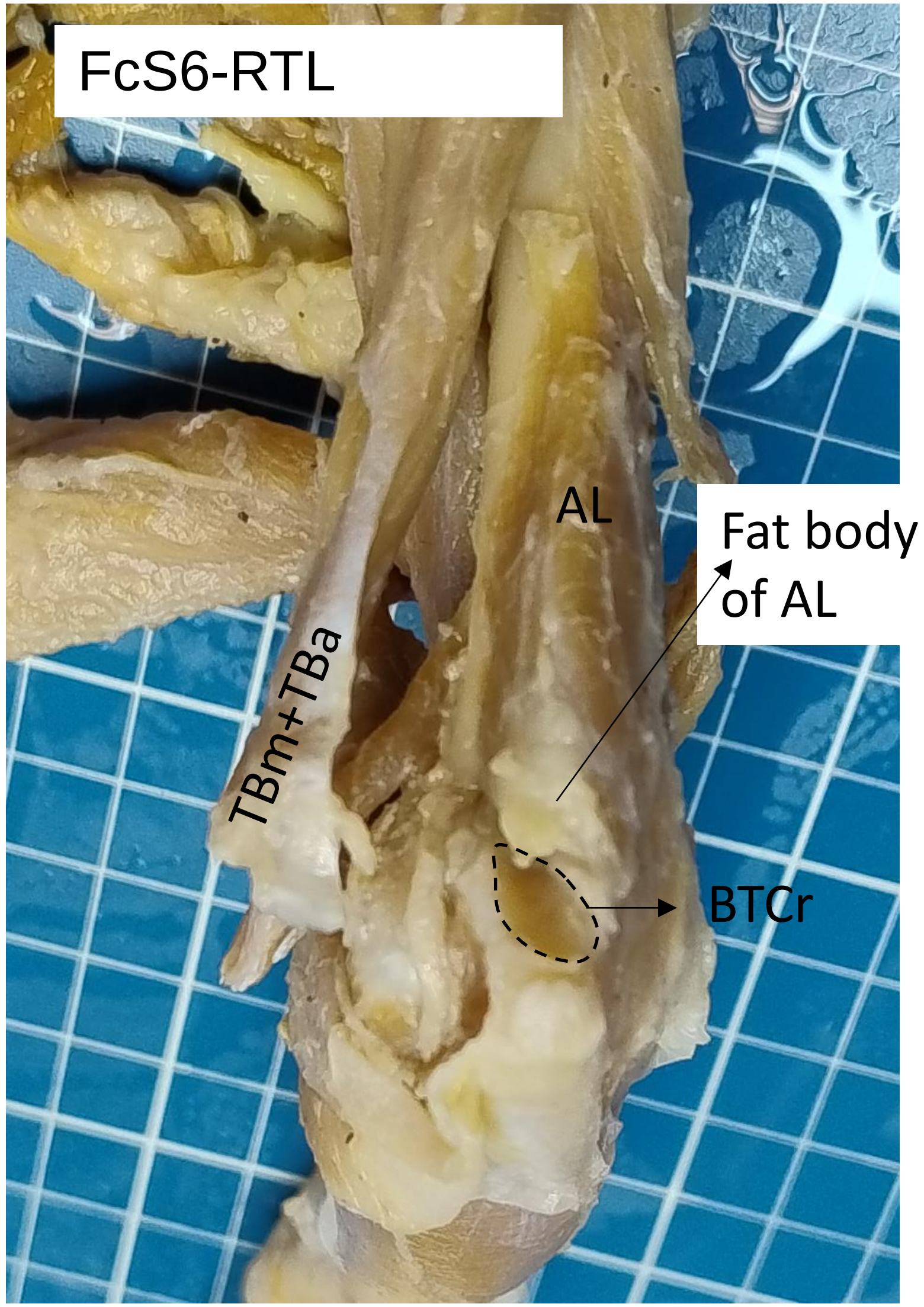
FcS6-RTL
AL
Fat body of AL
TBm+TBa
BTCr

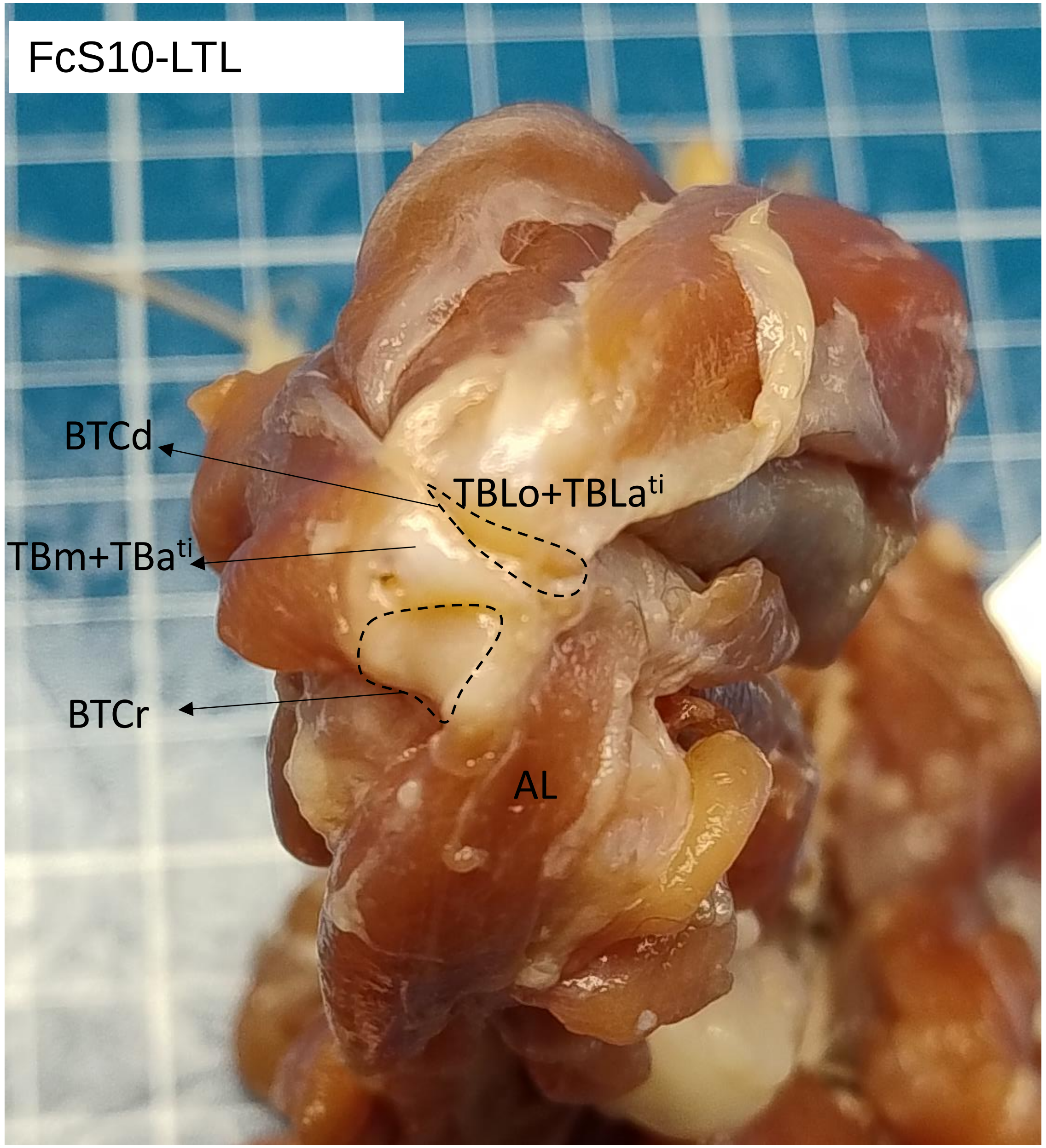
FcS10-LTL
BTCd
TBLo+TBLa^ti
TBm+TBa^ti
BTCr
AL

4.9

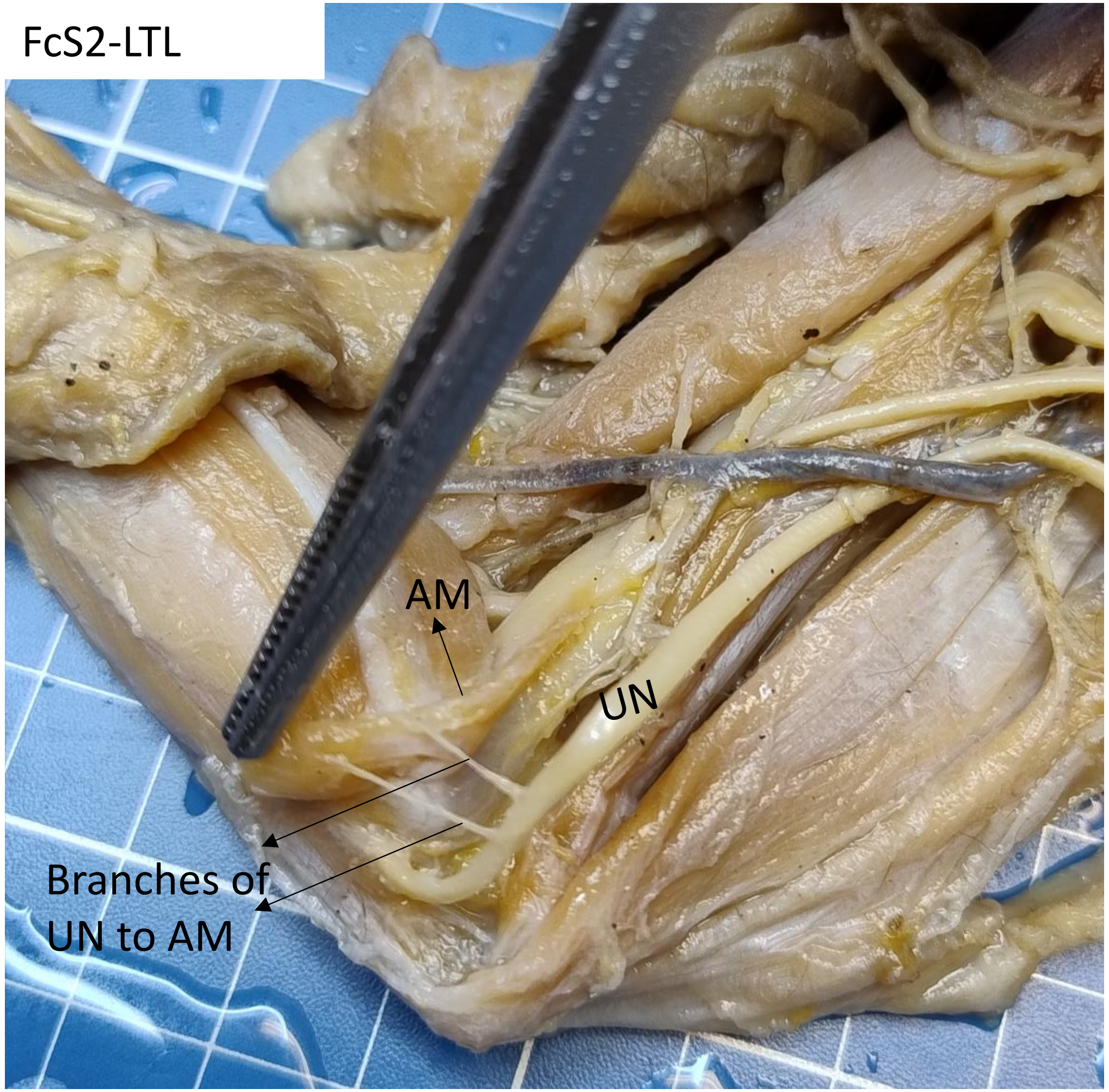
FcS2-LTL
AM
UN
Branches of
UN to AM

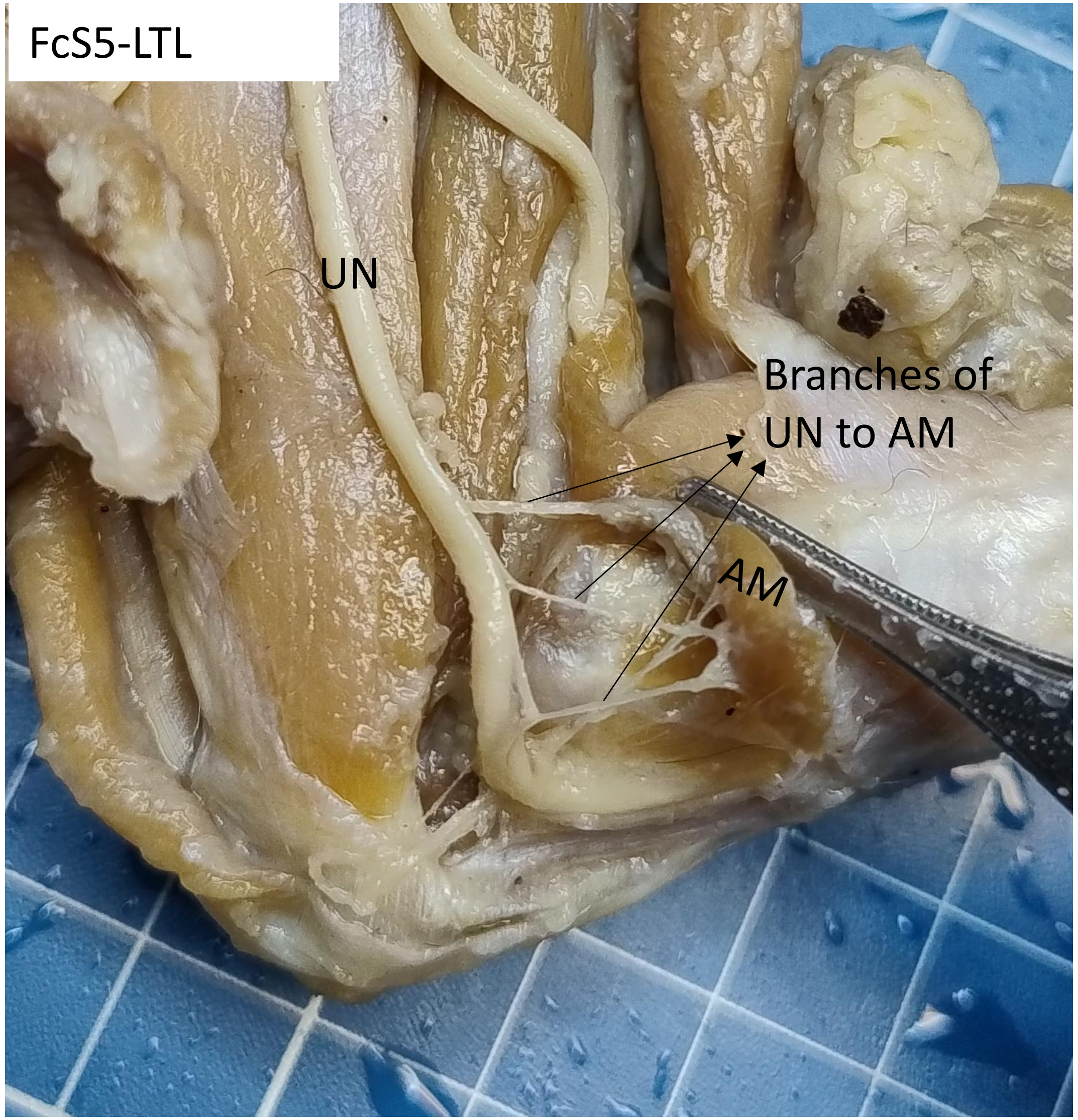
FcS5-LTL
UN
Branches of
UN to AM
AM

4.10

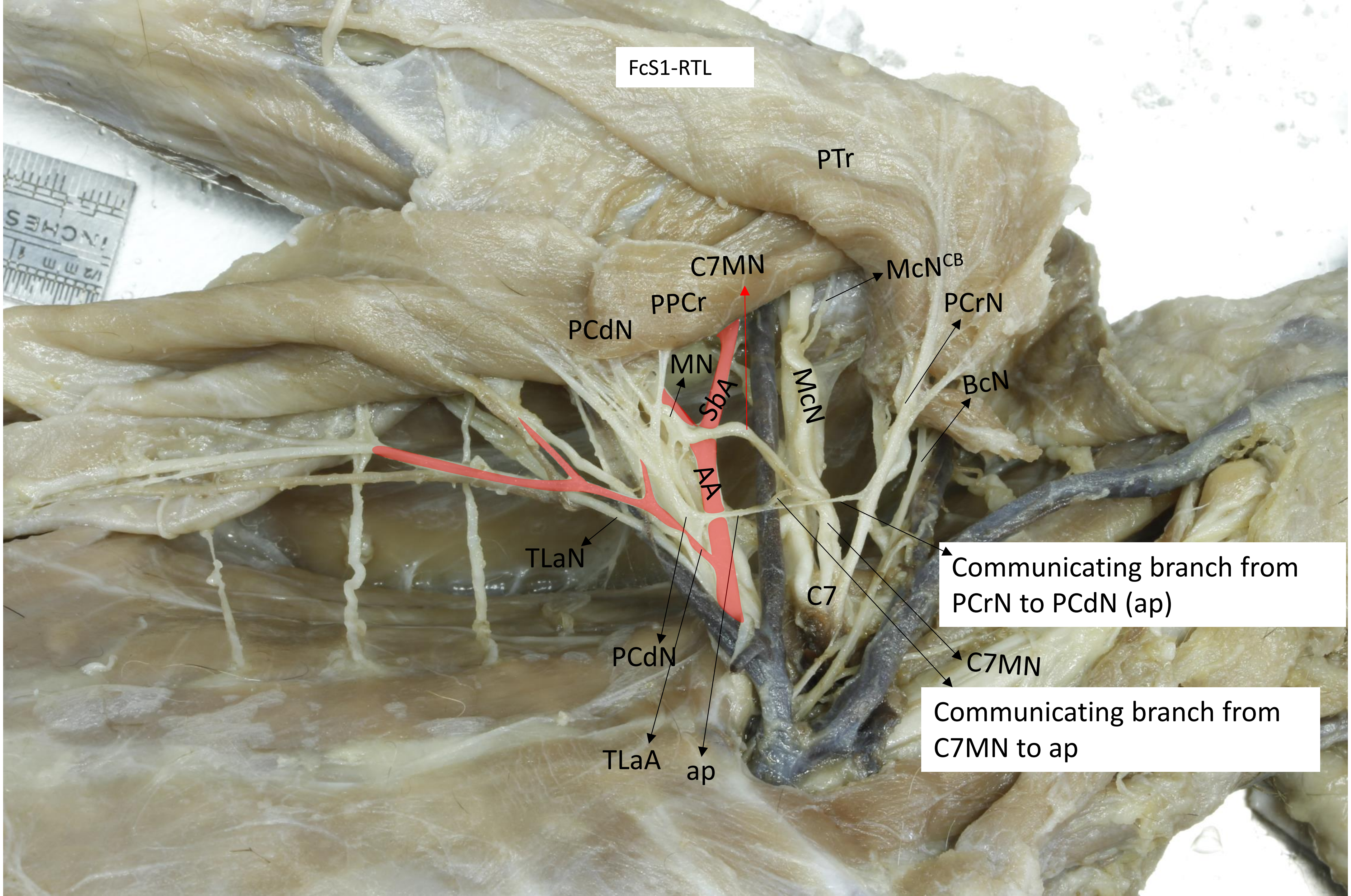
FcS1-RTL
PTr
C7MN
McN^{CB}
PPCr
PCrN
PCdN
MN
SbA
McN
BcN
AA
TLaN
C7
Communicating branch from
PCrN to PCdN (ap)
PCdN
C7MN
Communicating branch from
C7MN to ap
TLaA
ap

4.11

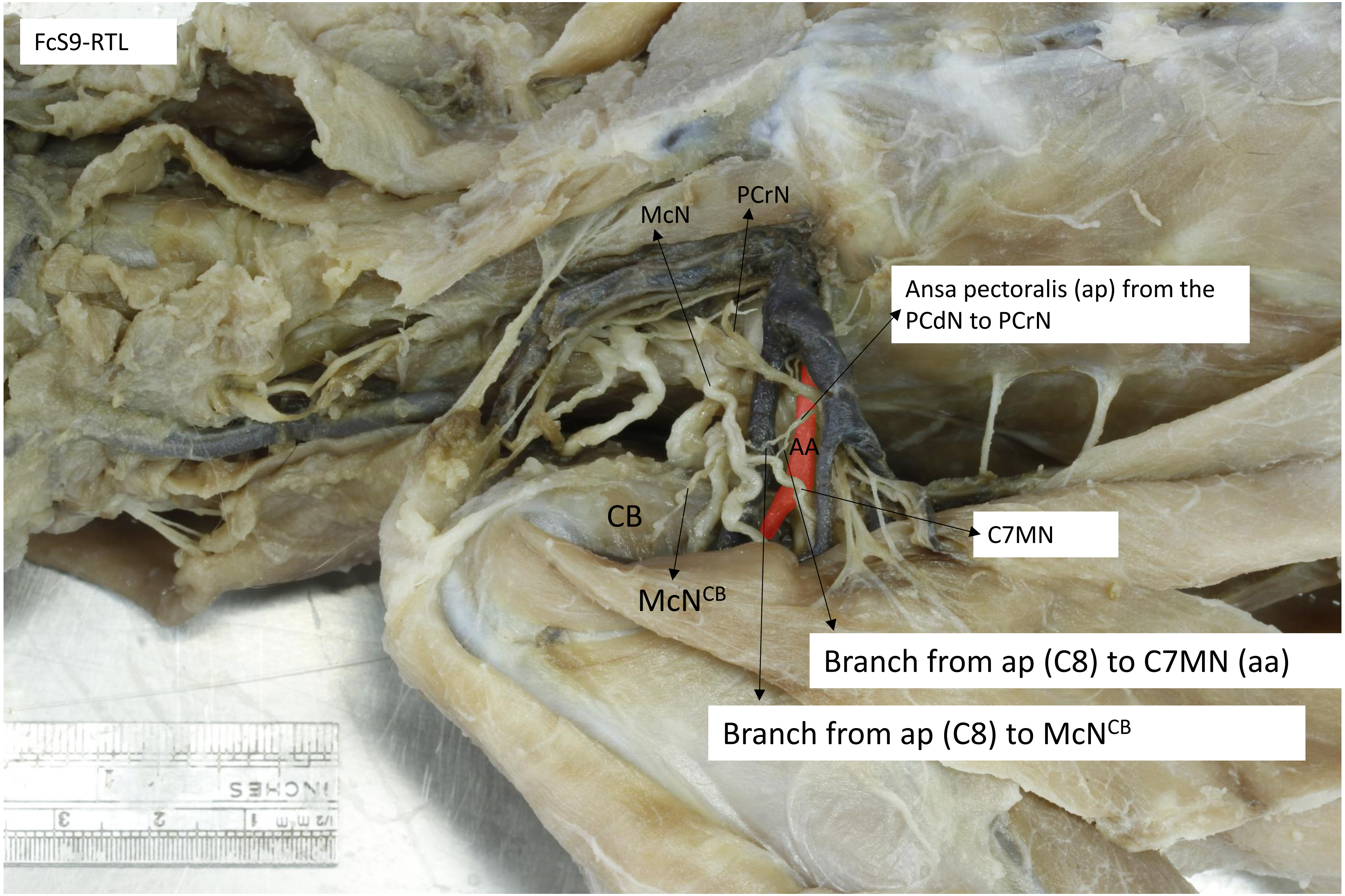

FcS9-RTL
PCrN
McN
Ansa pectoralis (ap) from the PCdN to PCrN
AA
C7MN
CB
McN^{CB}
Branch from ap (C8) to C7MN (aa)
Branch from ap (C8) to McN^{CB}
INCHES
1
2
3

4.12

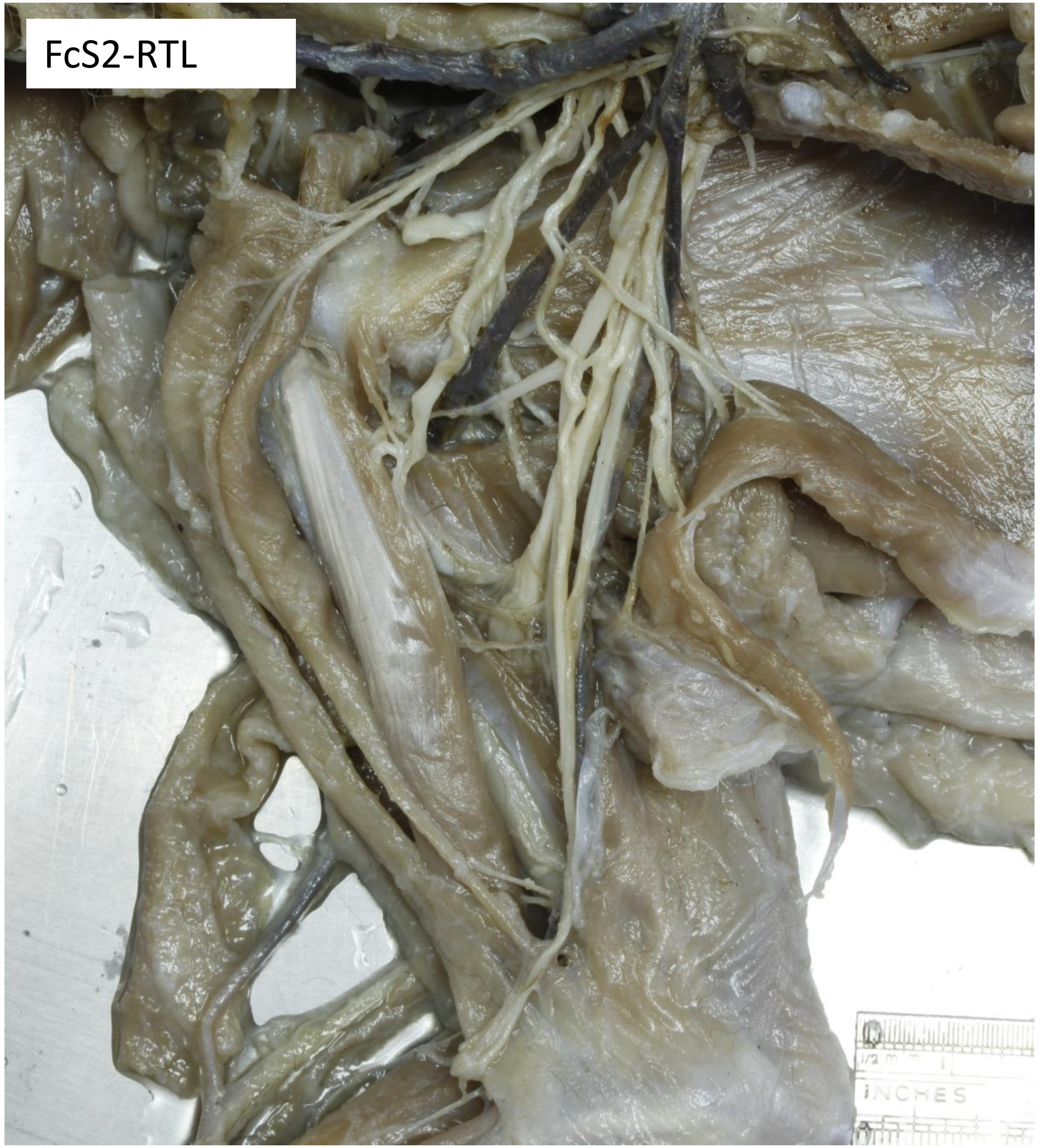

FcS2-RTL

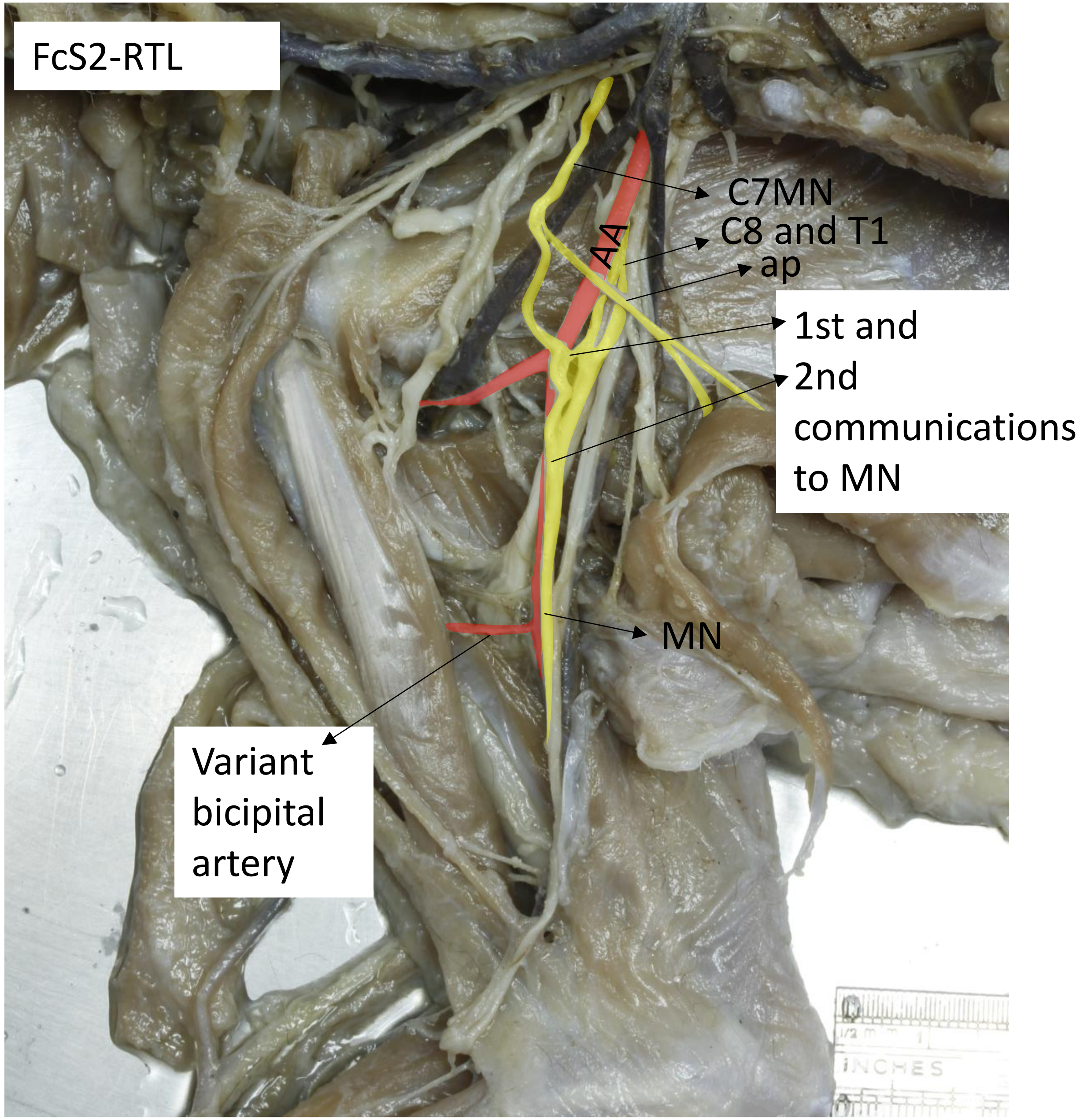

FcS2-RTL
C7MN
C8 and T1
ap
AA
1st and
2nd
communications
to MN
MN
Variant
bicipital
artery

4.13

FcS7-LTL

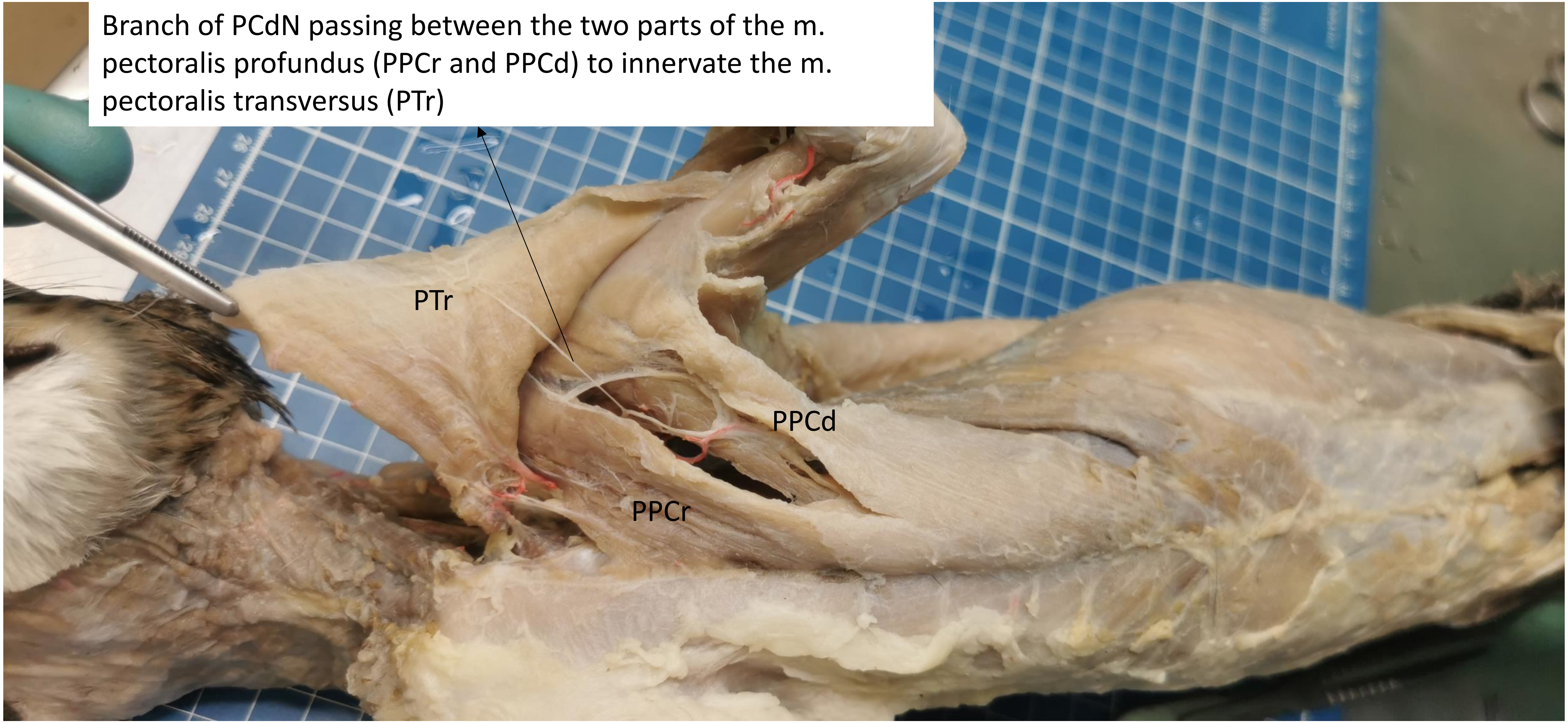
Branch of PCdN passing between the two parts of the m. pectoralis profundus (PPCr and PPCd) to innervate the m. pectoralis transversus (PTr)
PTr
PPCd
PPCr

4.14

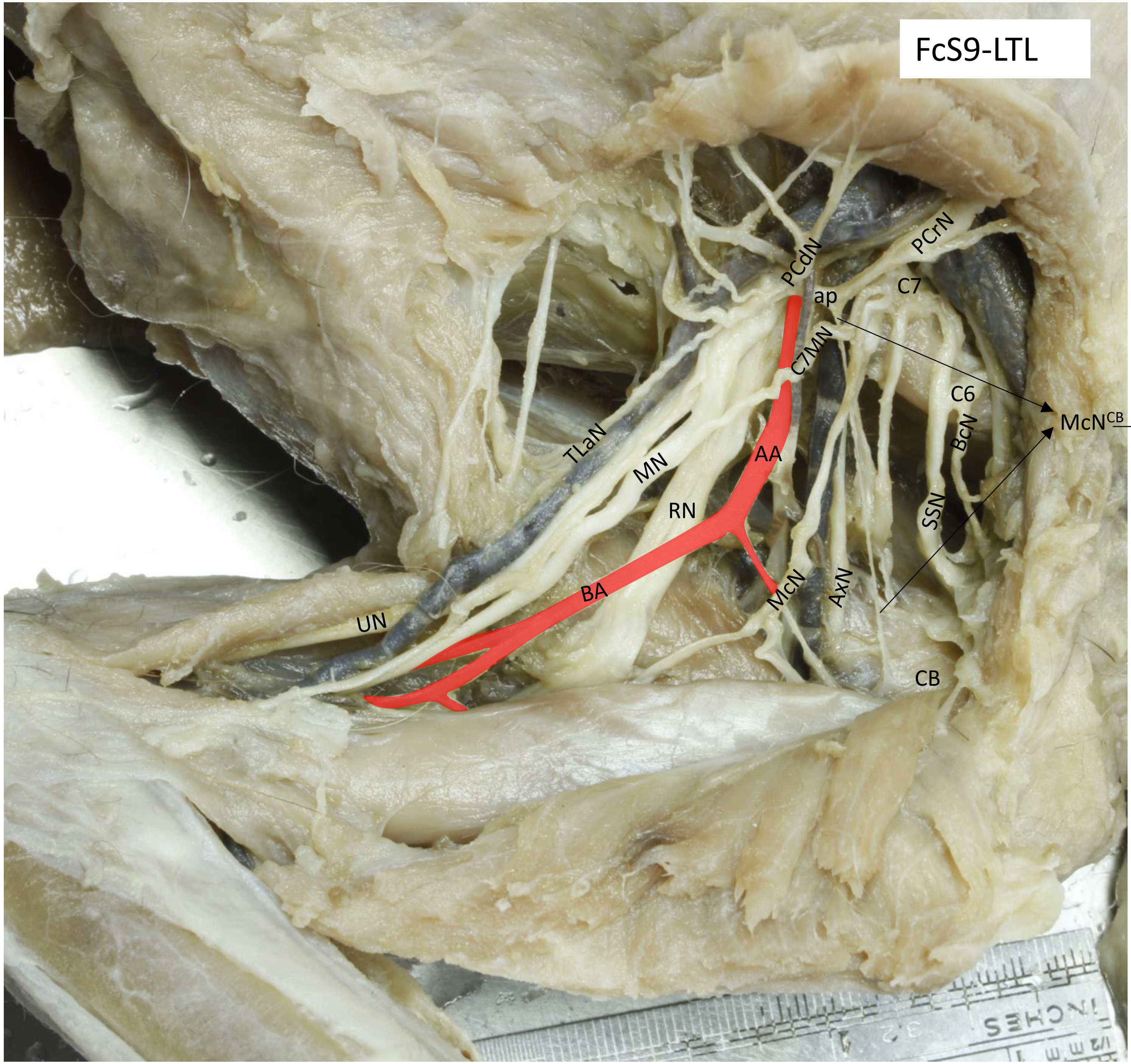

McN^CB → This branch originated from ap (C8)

4.15

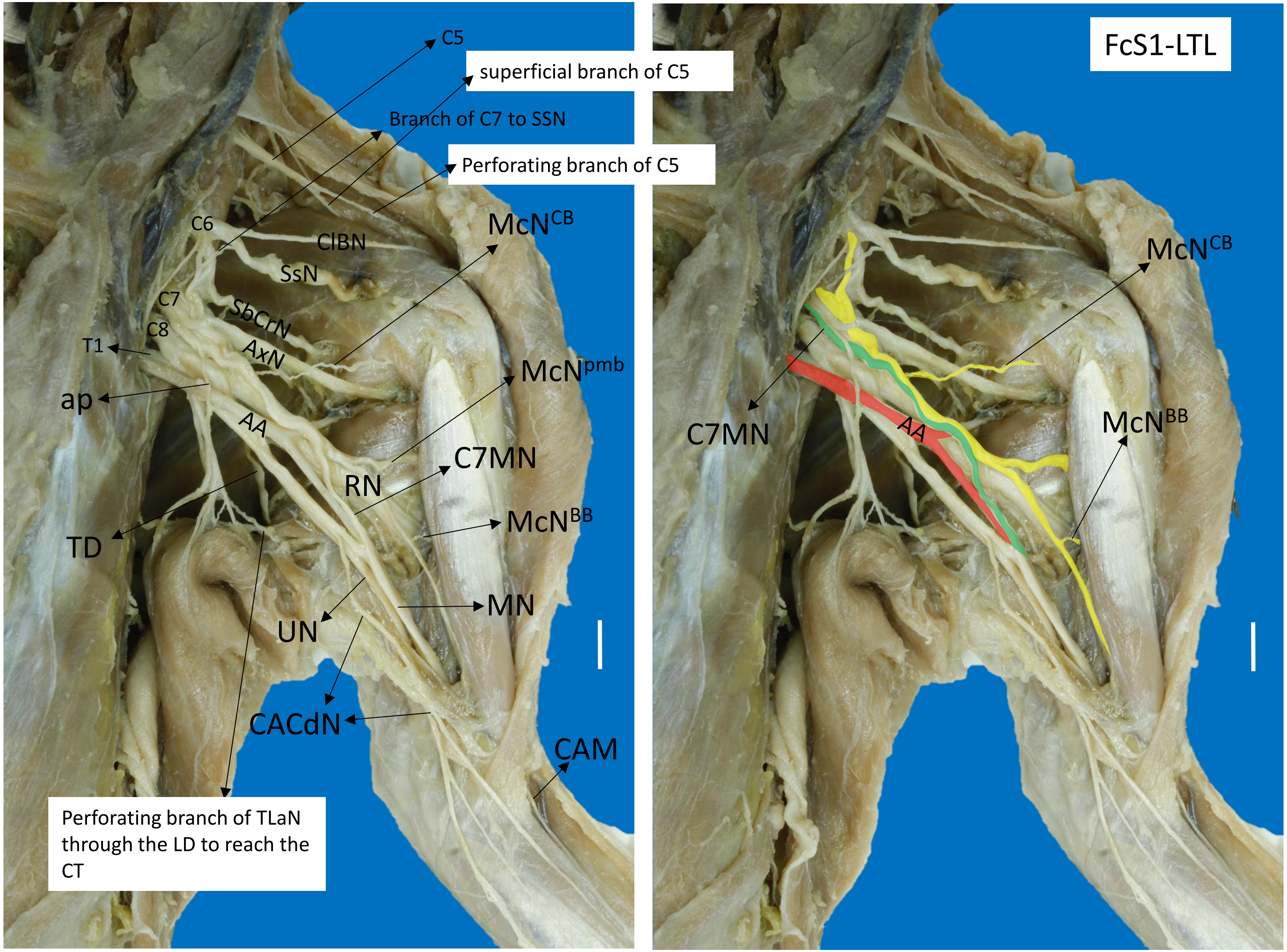
C5
superficial branch of C5
Branch of C7 to SSN
Perforating branch of C5
C6
ClBN
SsN
McN^CB
C7
SbCrN
C8
T1
AxN
McN^pmb
ap
AA
C7MN
RN
McN^BB
TD
MN
UN
CACdN
CAM
Perforating branch of TLaN
through the LD to reach the
CT
FcS1-LTL
McN^CB
C7MN
AA
McN^BB
