# Supplementary material for: Updating the forelimb anatomy of the domestic cat (Felis catus, Felidae) based on evolutionary inferences of its muscles and nerves I: Shoulder and brachium
Source: J Anat. 2026 Apr 19:10.1111/joa.70151. Online ahead of print. doi: 10.1111/joa.70151 (PMC13398976; doi:10.1111/joa.70151)
Supplement: Supplementary file 4 — Supplementary material 4. Photographs of anatomical variants. [file JOA-9999-0-s005.pdf]

4.1

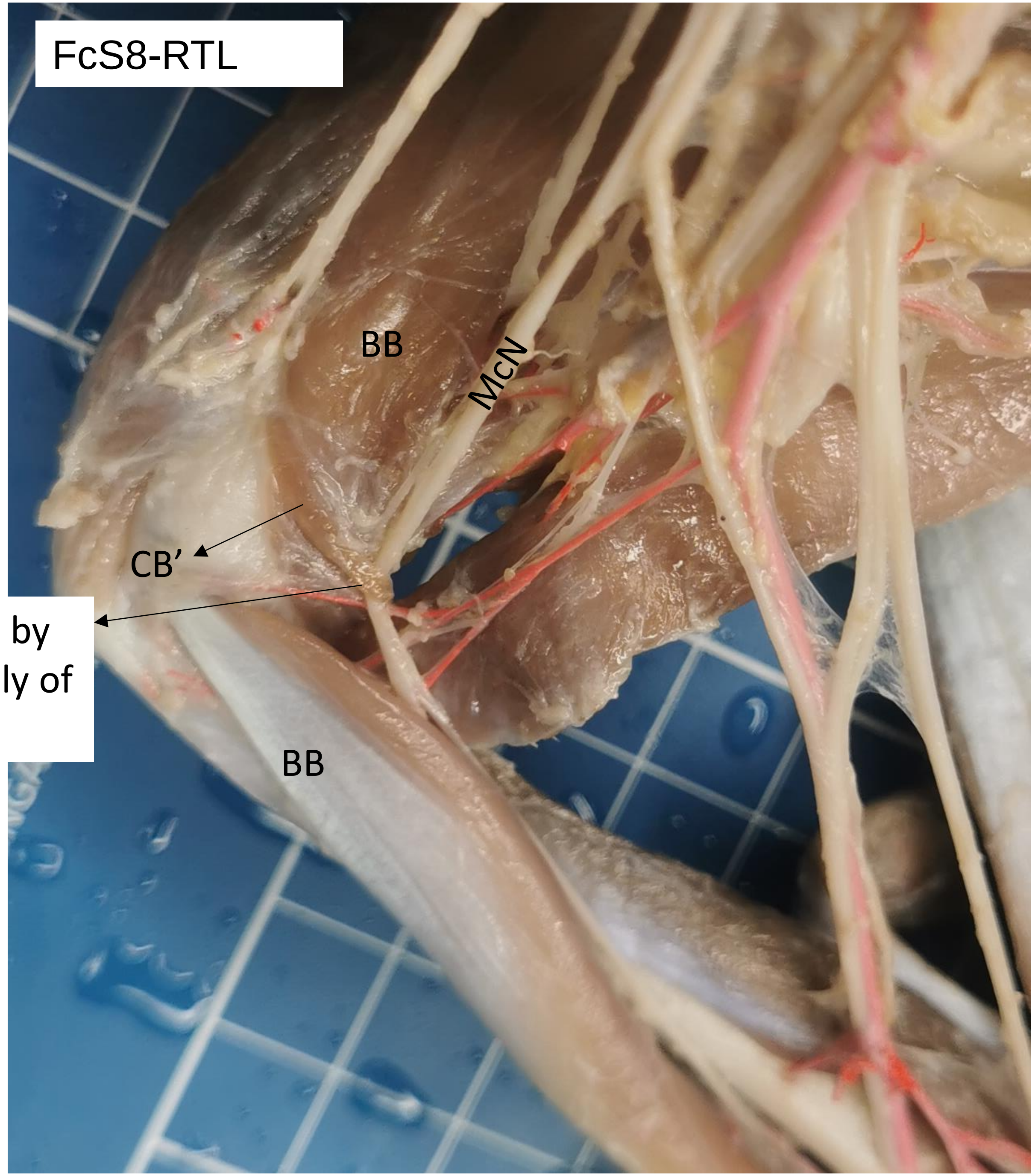

McN entrapment by the accessory belly of CB (CB’)

View without McN

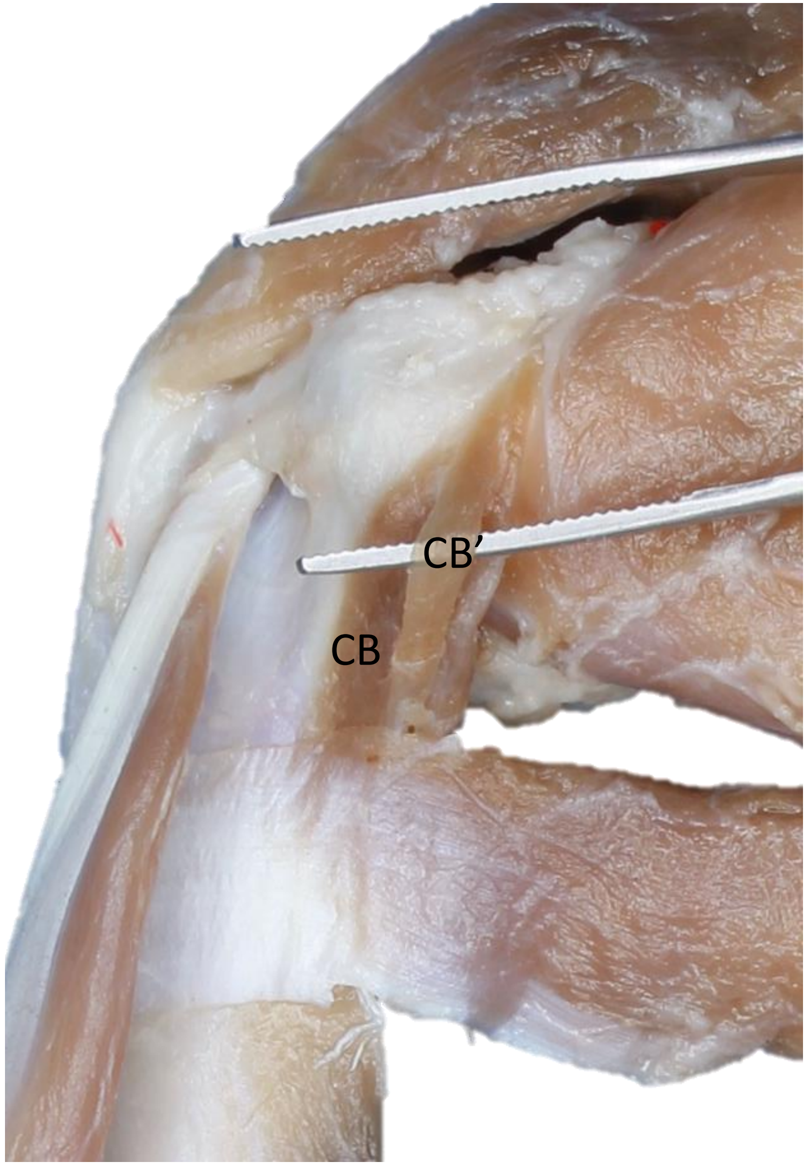

4.2

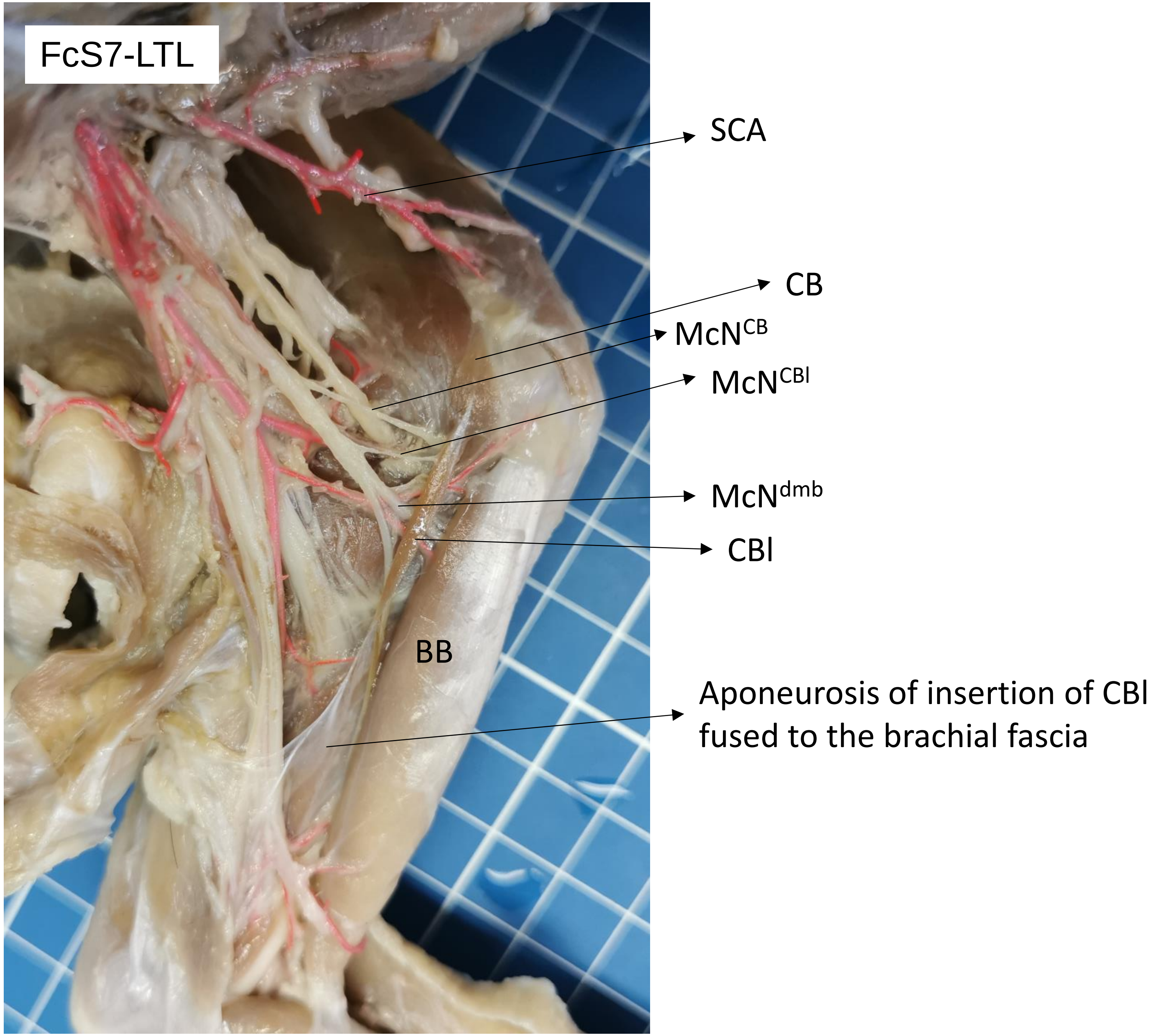
FcS7-LTL
SCA
CB
McN^{CB}
McN^{CBl}
McN^{dmb}
CBl
BB
Aponeurosis of insertion of CBl
fused to the brachial fascia

4.3

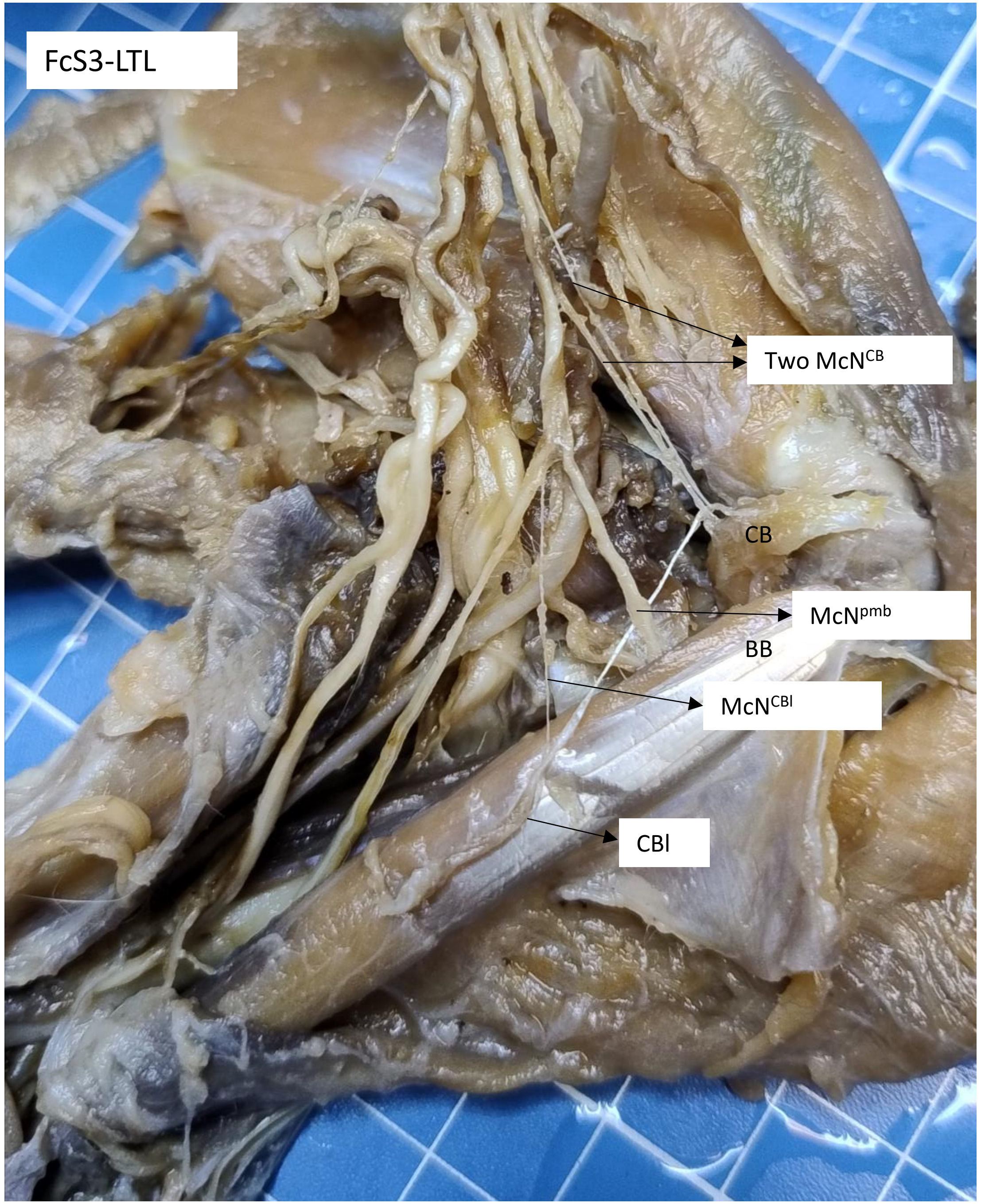

4.4

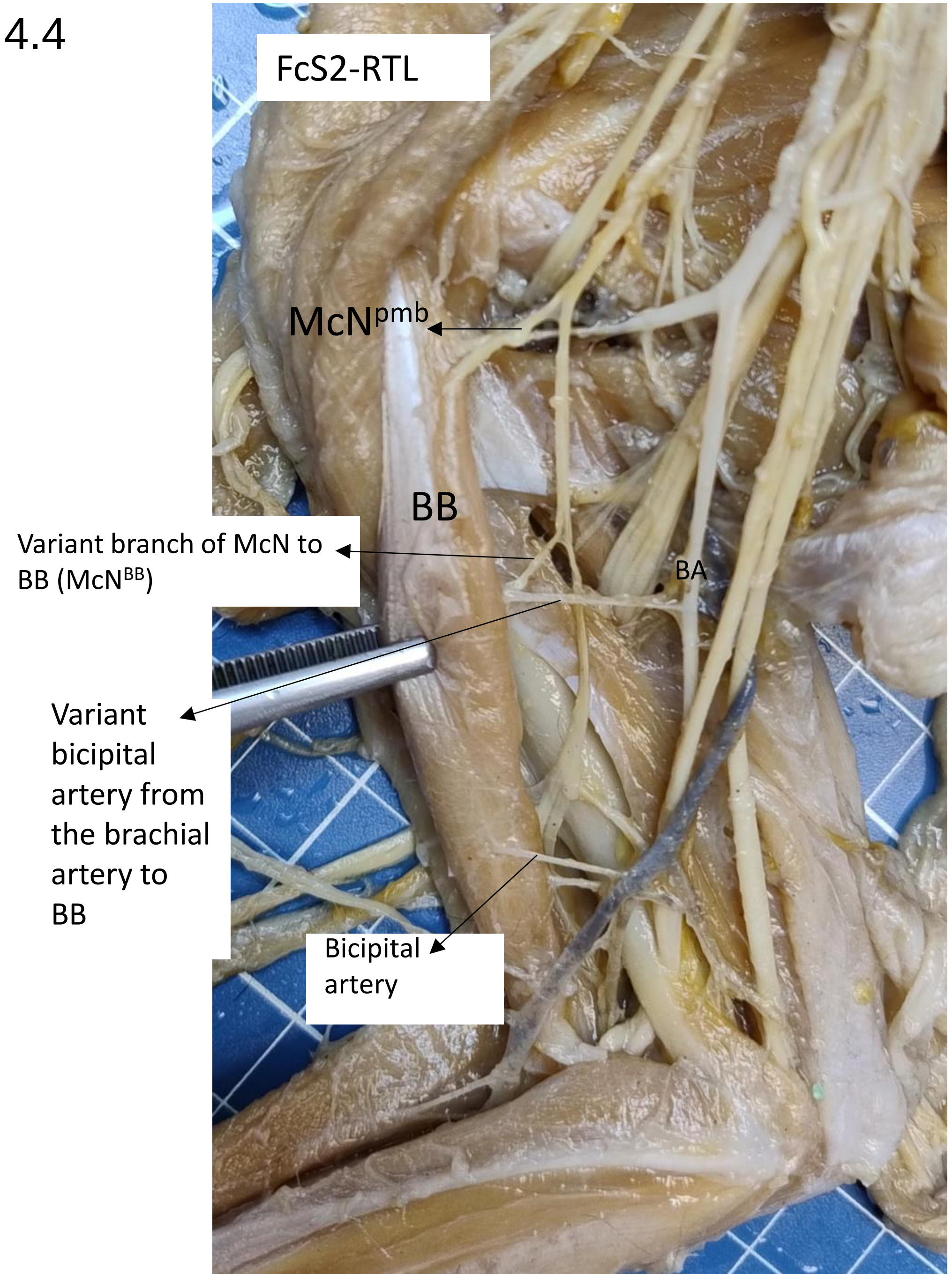
FcS2-RTL
McN^{pmb}
BB
Variant branch of McN to BB (McN^{BB})
BA
Variant bicipital artery from the brachial artery to BB
Bicipital artery

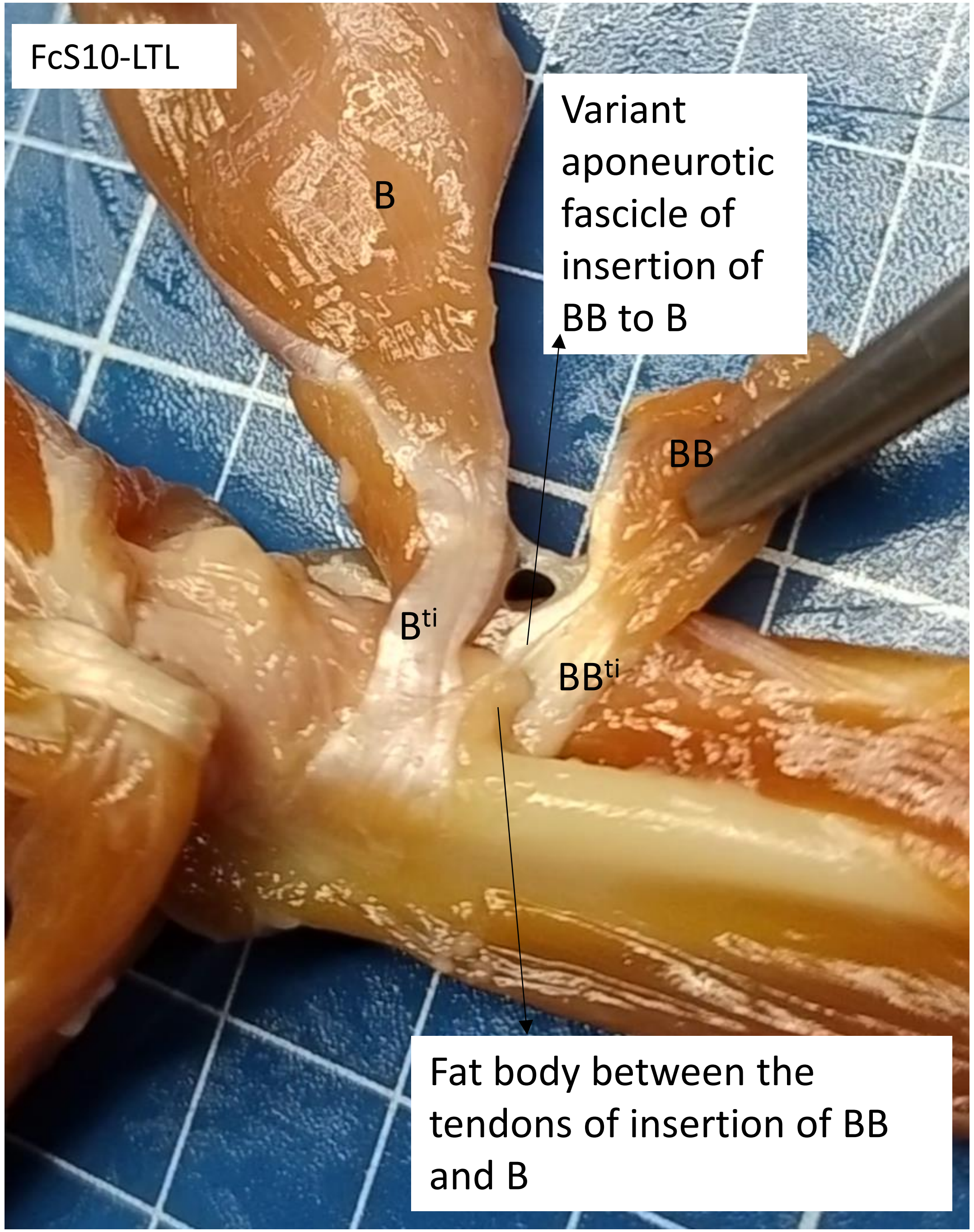
FcS10-LTL
B
Variant aponeurotic fascicle of insertion of BB to B
BB
B^{ti}
BB^{ti}
Fat body between the tendons of insertion of BB and B

4.5

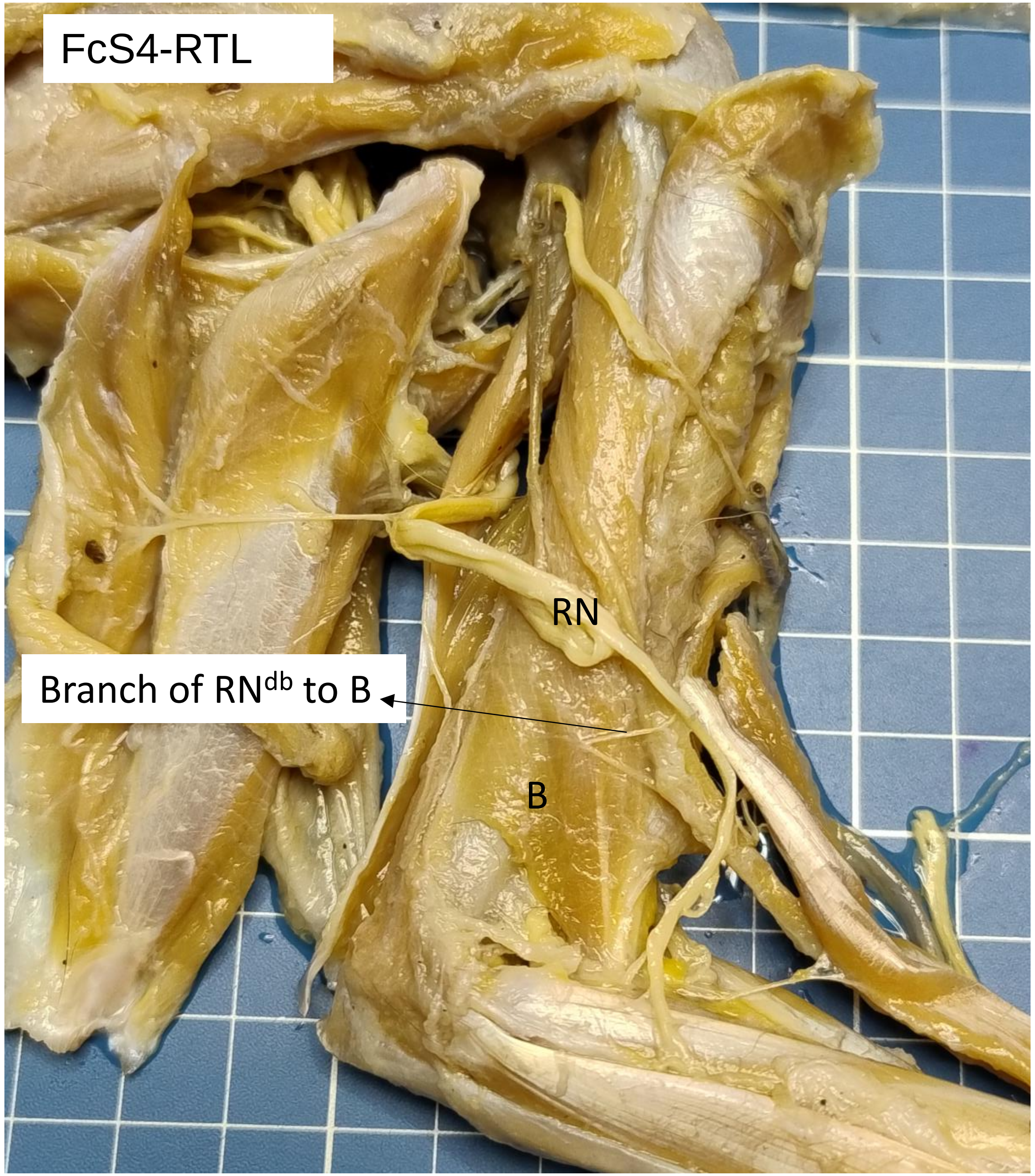

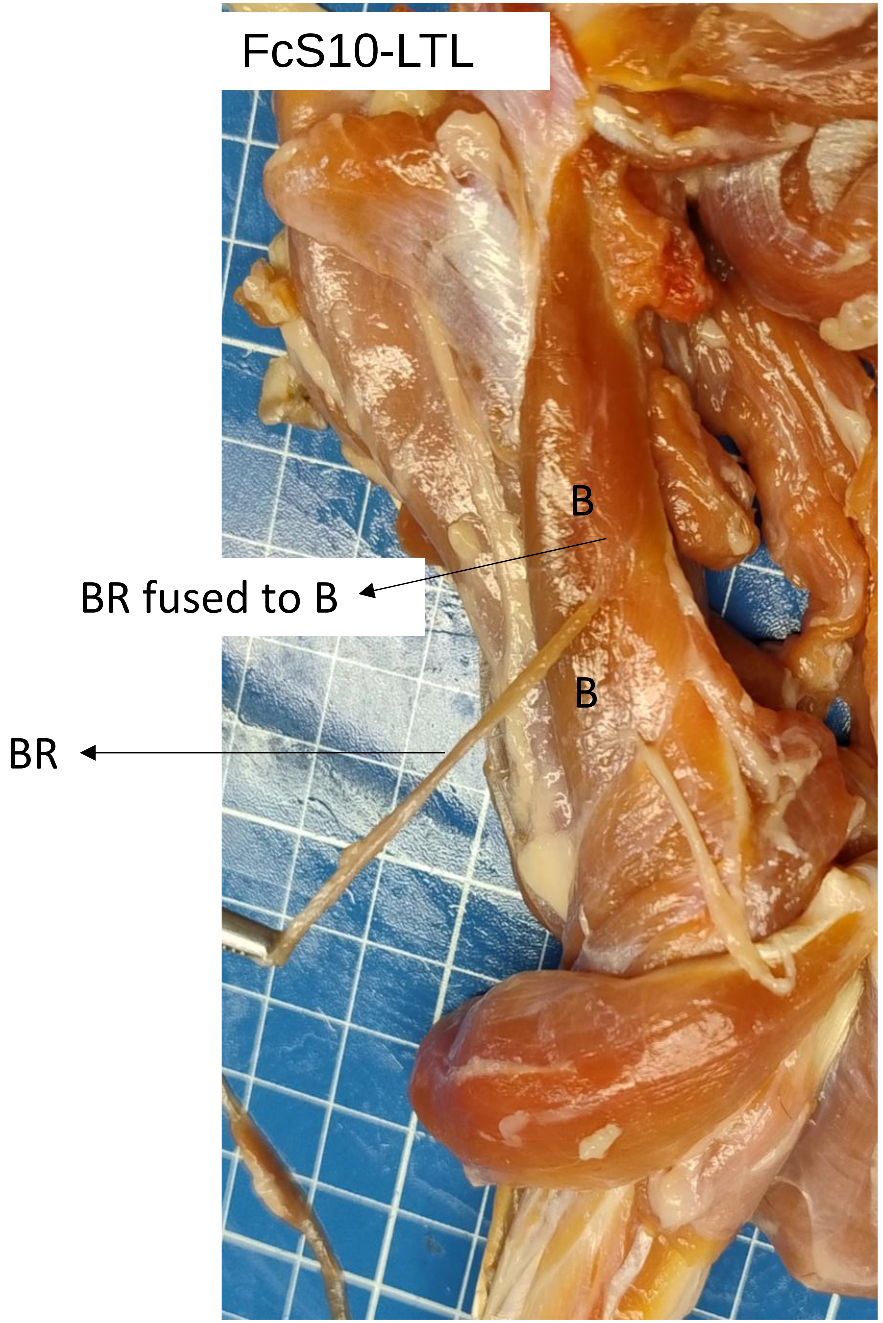

4.6

FcS5-LTL

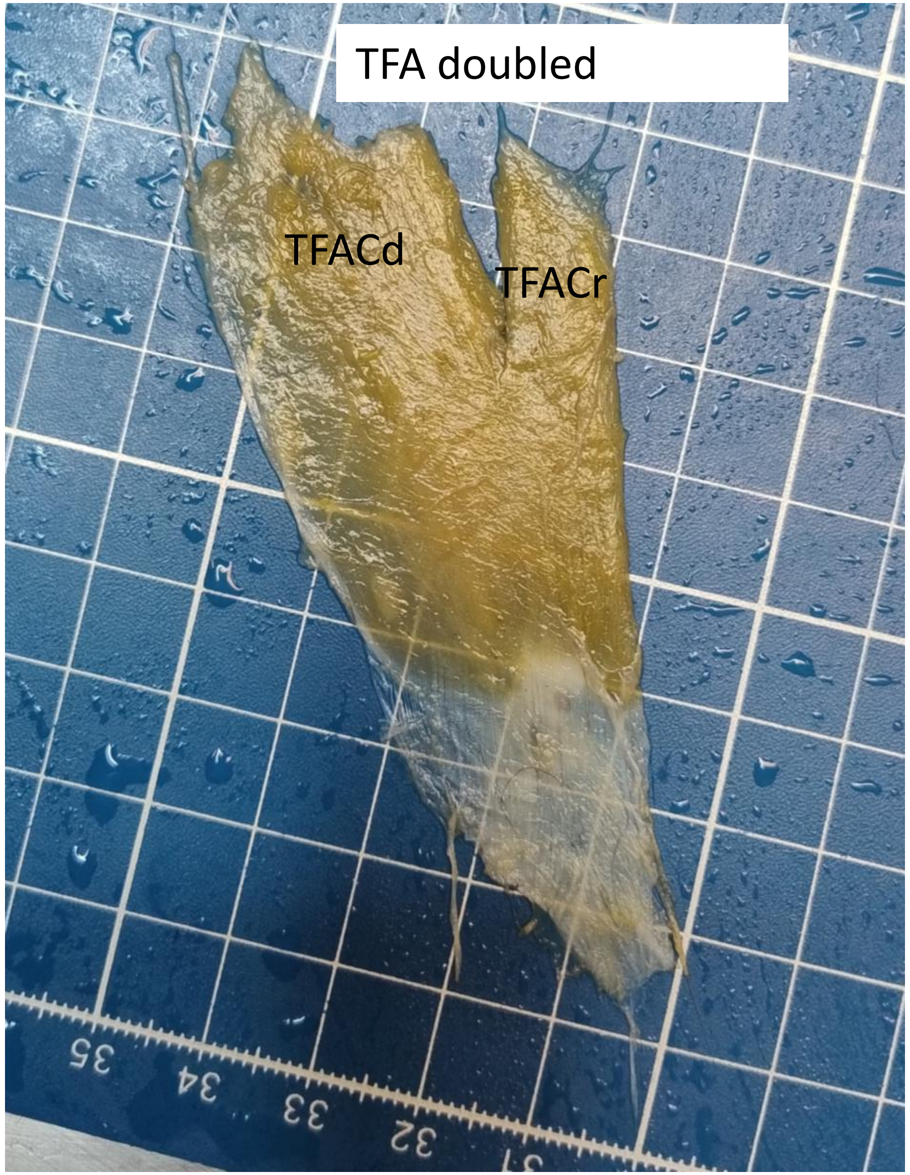

FcS5-LTL

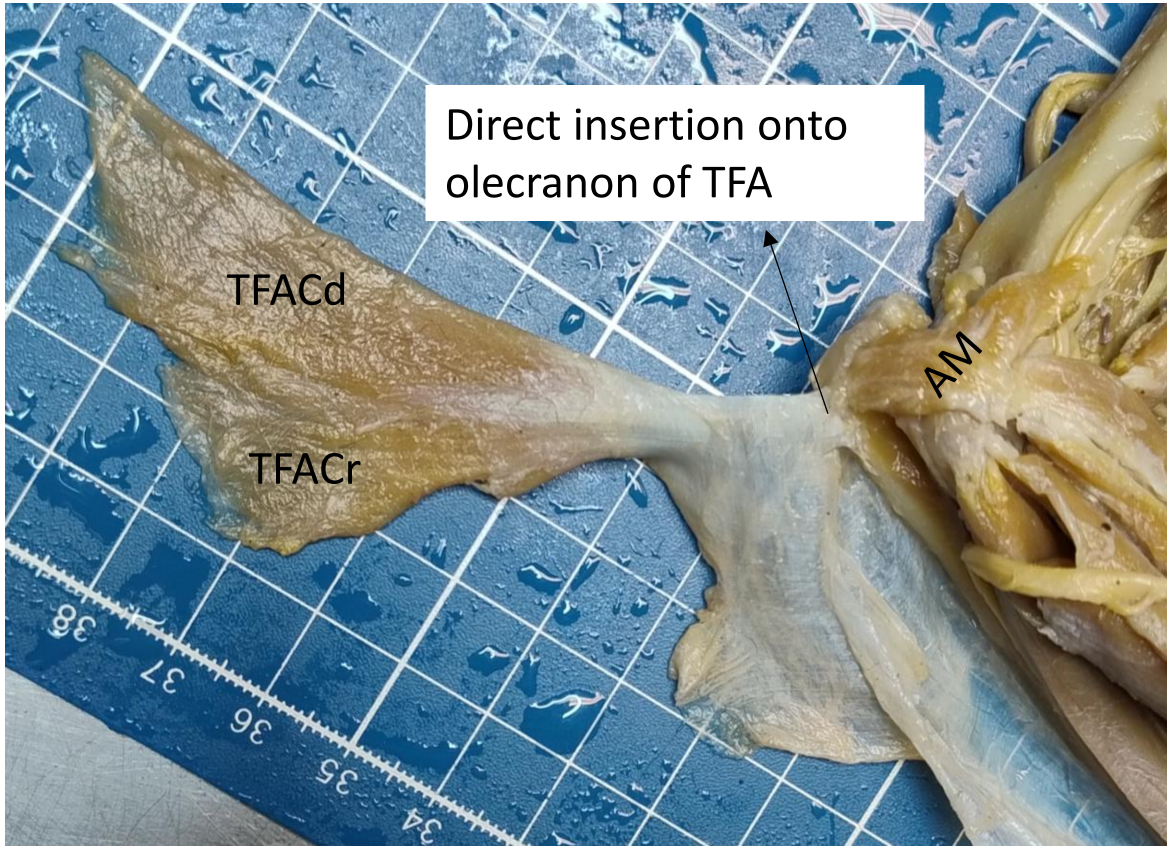

4.7

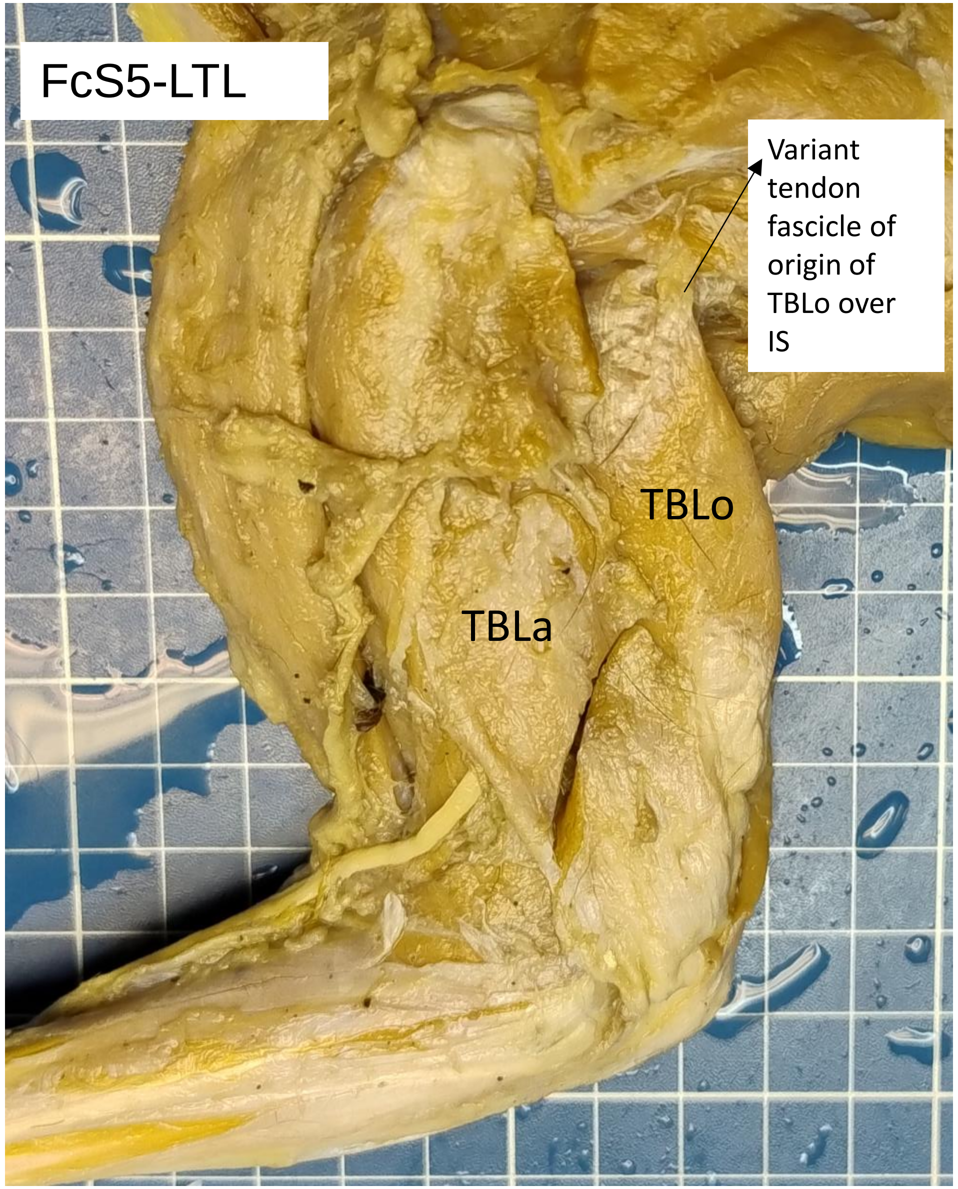
FcS5-LTL
Variant tendon fascicle of origin of TBLo over IS
TBLo
TBLa

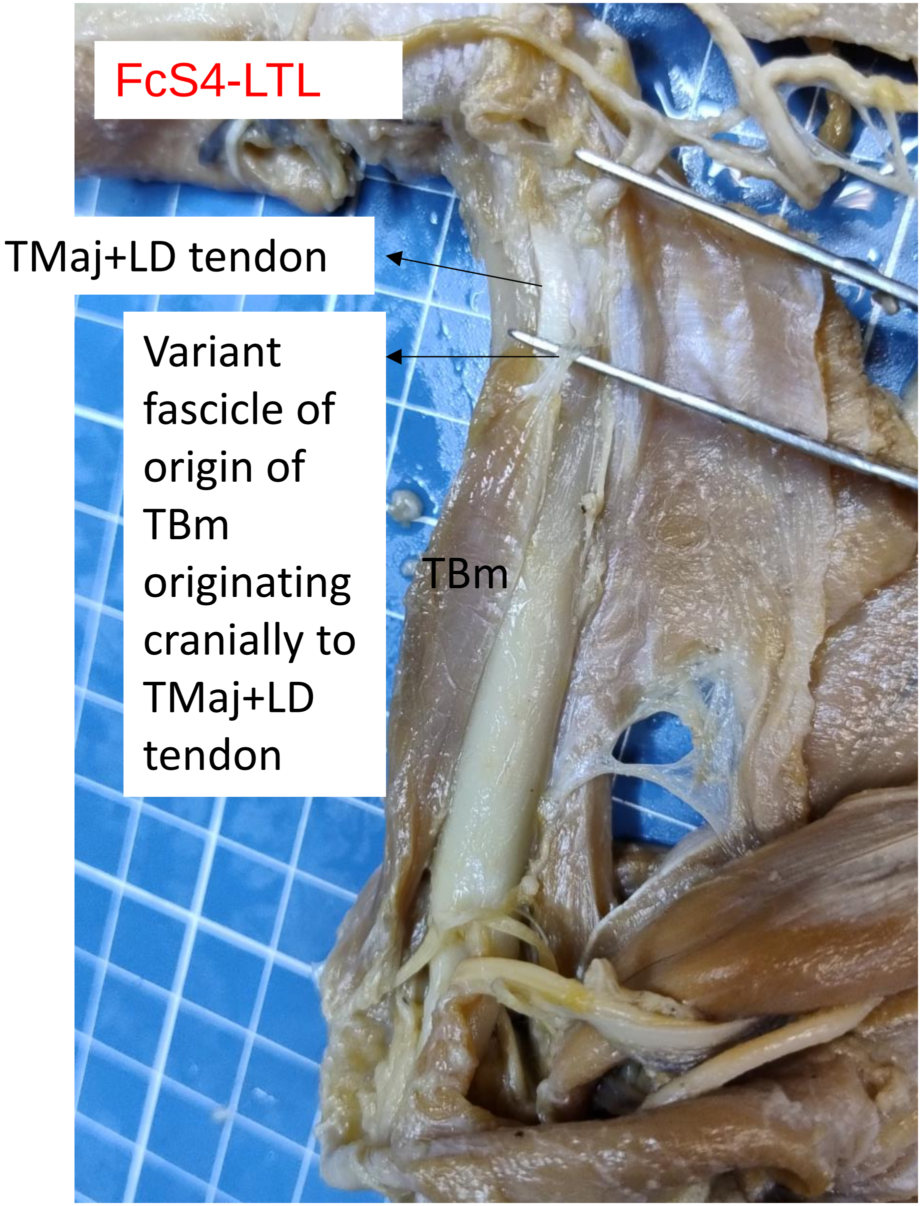
FcS4-LTL
TMaj+LD tendon
Variant fascicle of origin of TBm originating cranially to TMaj+LD tendon
TBm

4.8

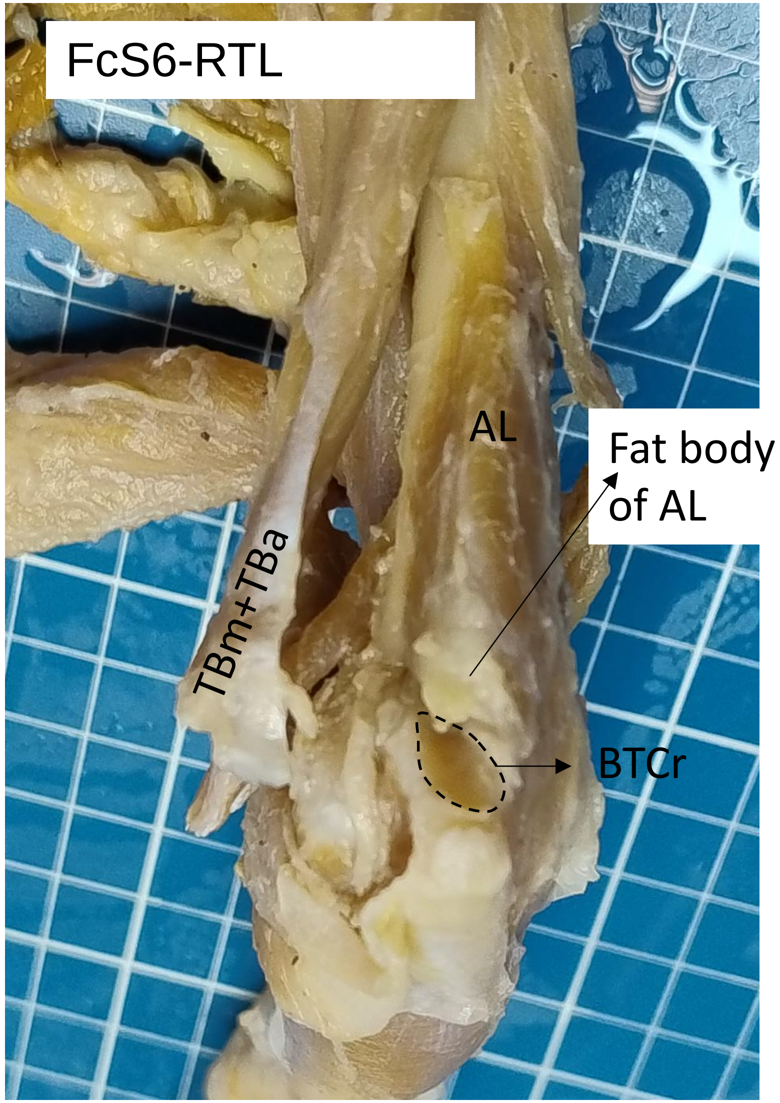
FcS6-RTL
AL
Fat body of AL
TBm+TBa
BTCr

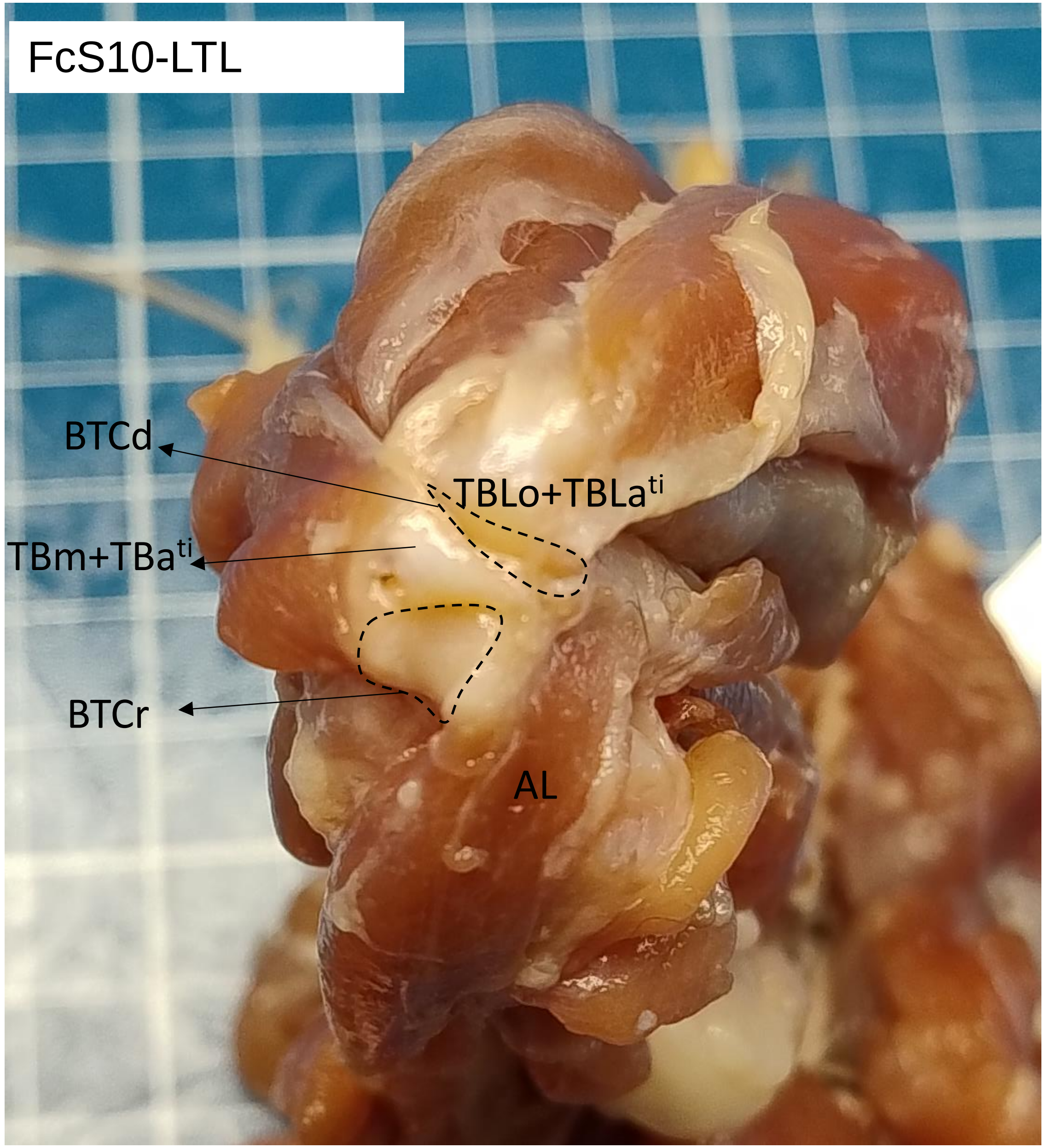
FcS10-LTL
BTCd
TBLo+TBLa^ti
TBm+TBa^ti
BTCr
AL

4.9

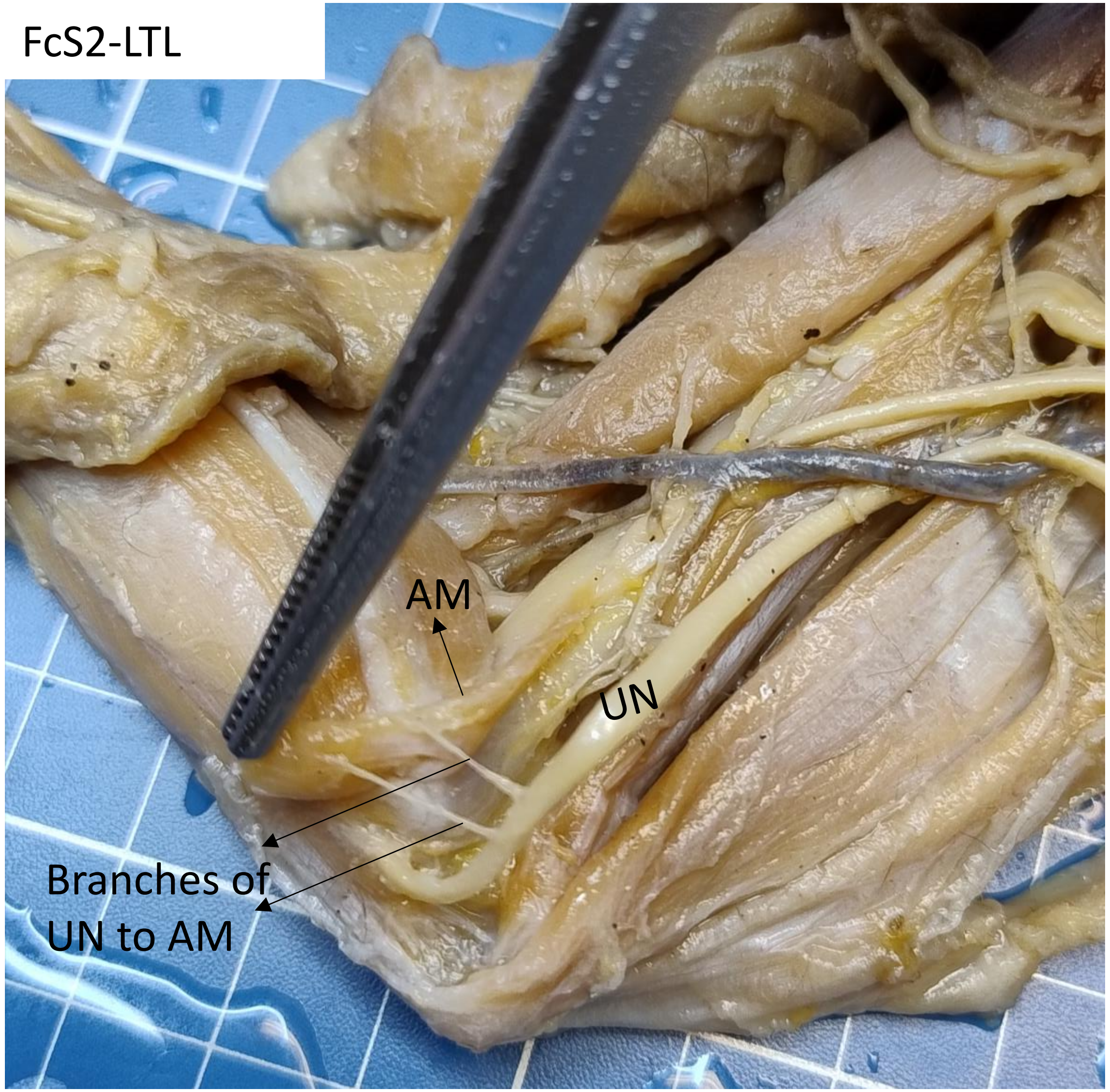
FcS2-LTL
AM
UN
Branches of
UN to AM

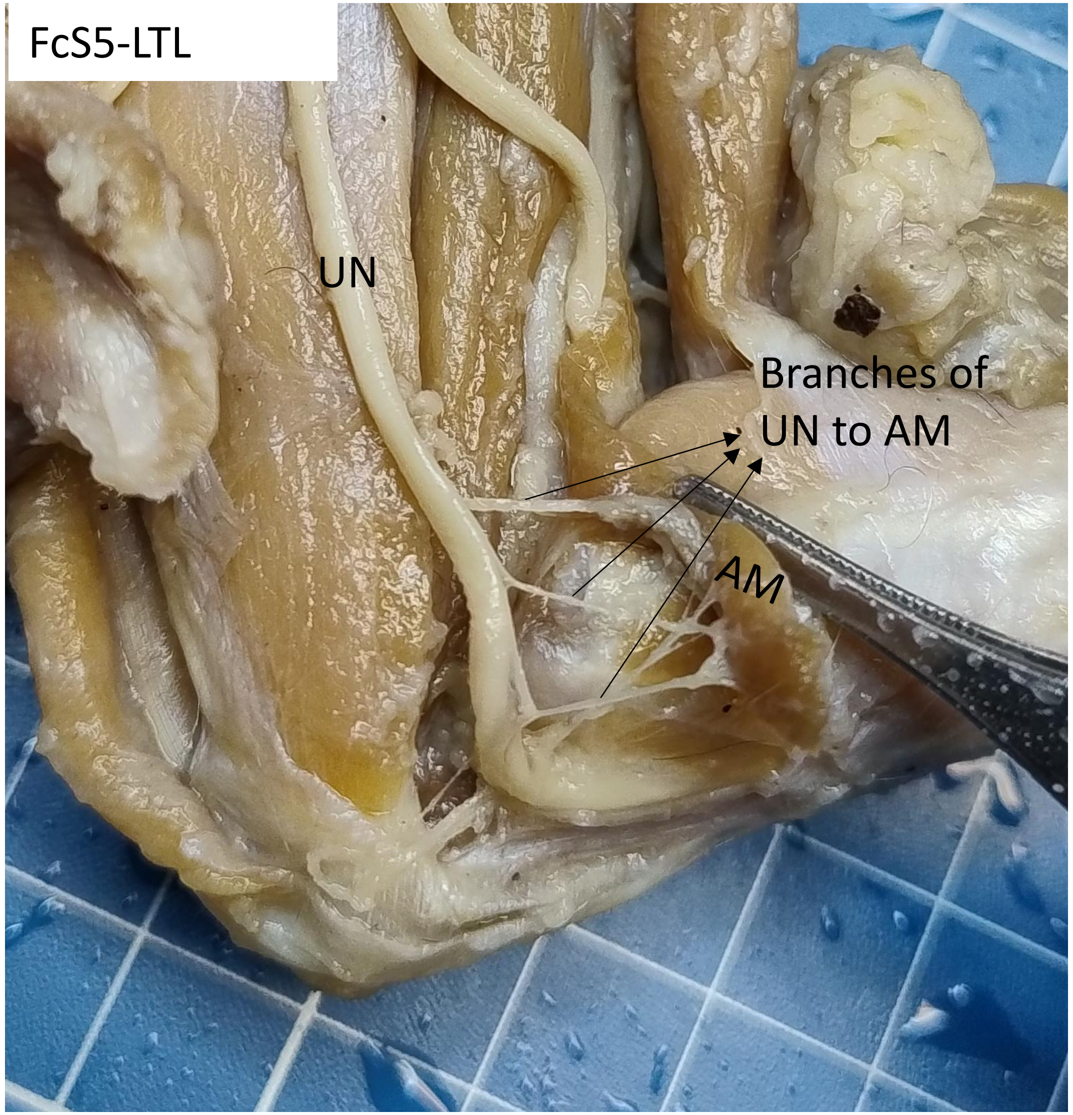
FcS5-LTL
UN
Branches of
UN to AM
AM

4.10

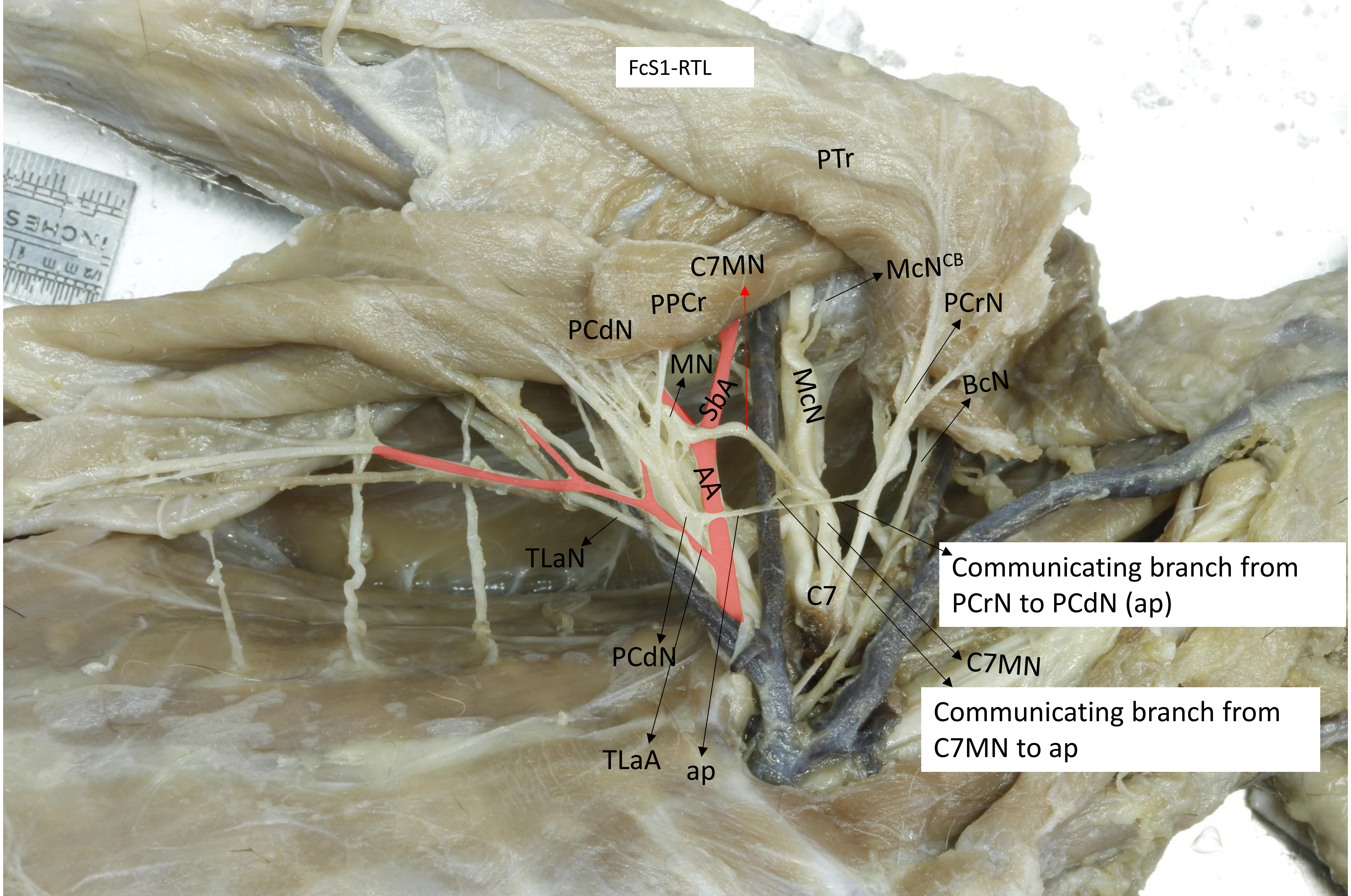
FcS1-RTL
PTr
C7MN
McN^{CB}
PPCr
PCrN
PCdN
MN
SbA
McN
BcN
AA
TLaN
C7
Communicating branch from PCrN to PCdN (ap)
C7MN
PCdN
Communicating branch from C7MN to ap
TLaA
ap

4.11

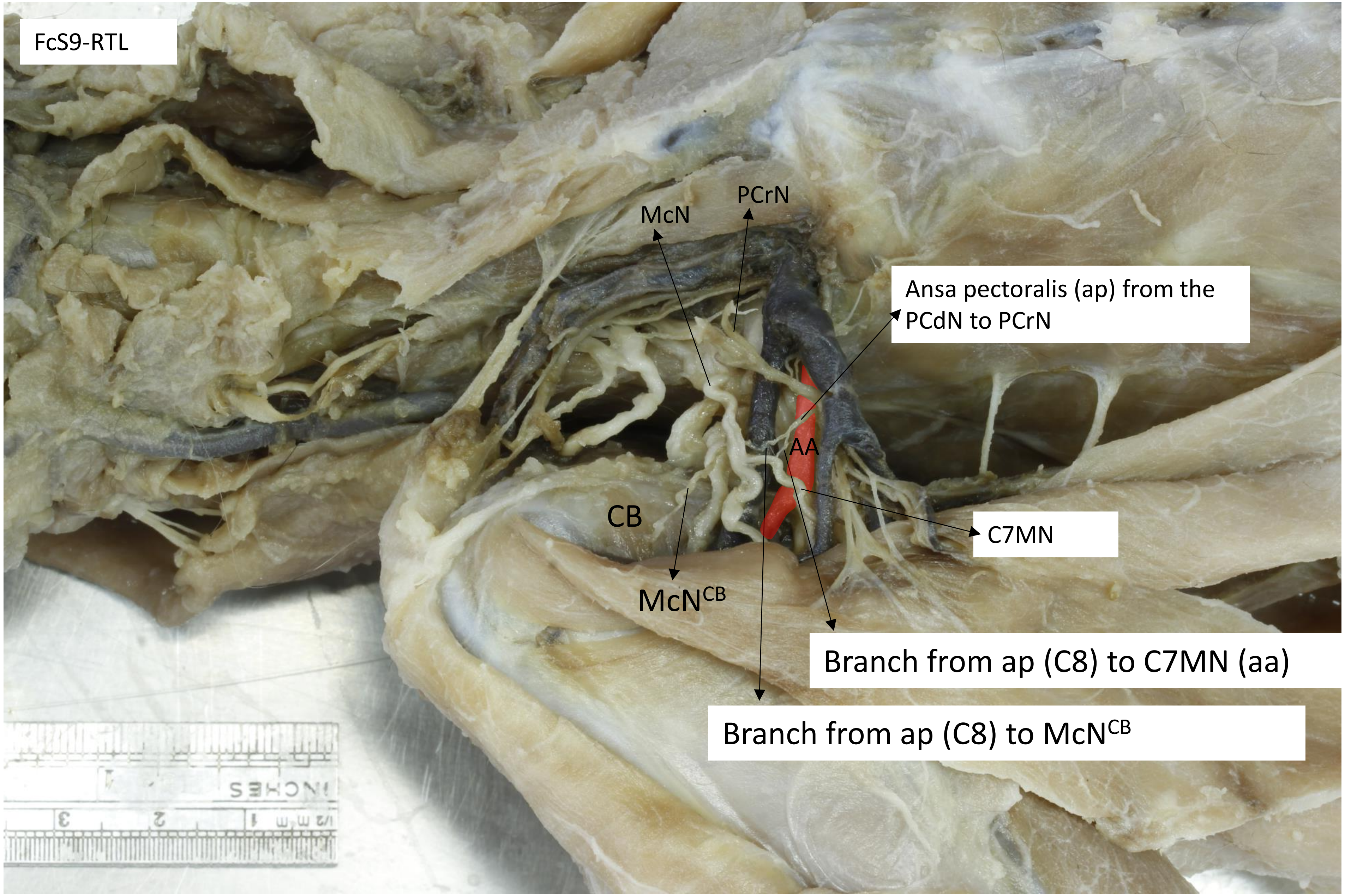
FcS9-RTL
PCrN
McN
Ansa pectoralis (ap) from the PCdN to PCrN
AA
CB
C7MN
McNCB
Branch from ap (C8) to C7MN (aa)
Branch from ap (C8) to McNCB
INCHES
1
2
3

4.12

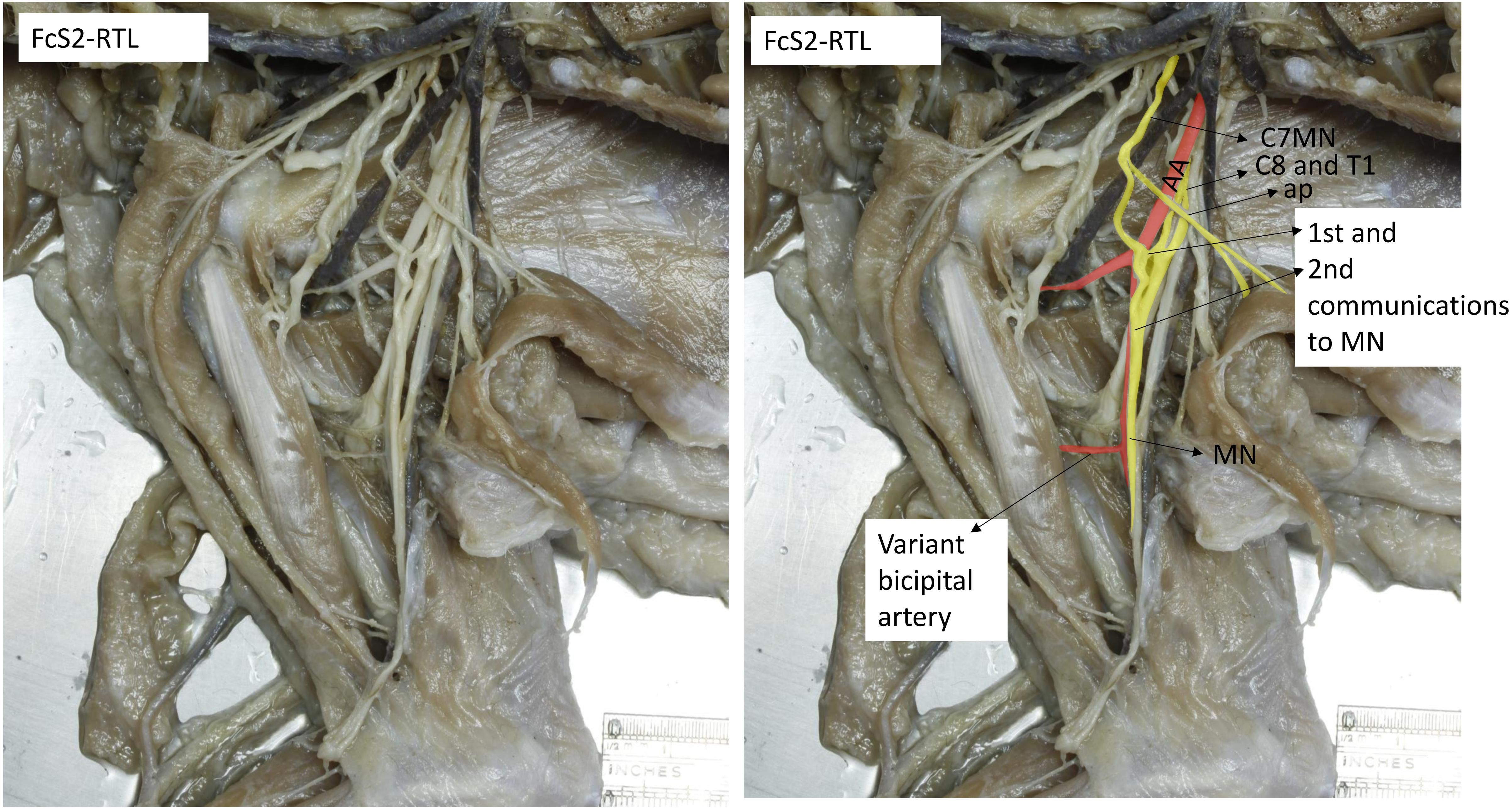
FcS2-RTL
FcS2-RTL
C7MN
C8 and T1
AA
ap
1st and
2nd
communications
to MN
MN
Variant
bicipital
artery
INCHES
INCHES

4.13

FcS7-LTL

Branch of PCdN passing between the two parts of the m. pectoralis profundus (PPCr and PPCd) to innervate the m. pectoralis transversus (PTr)

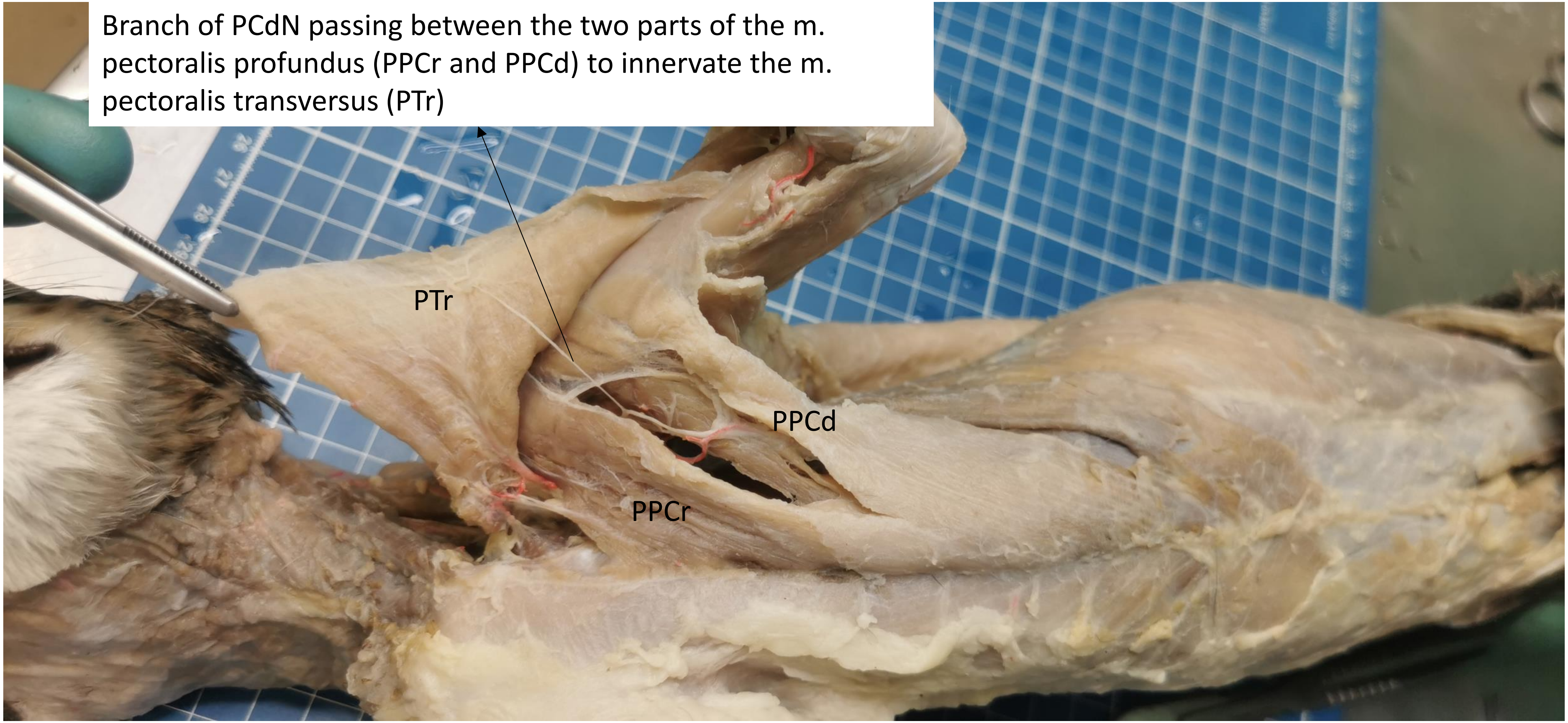

4.14

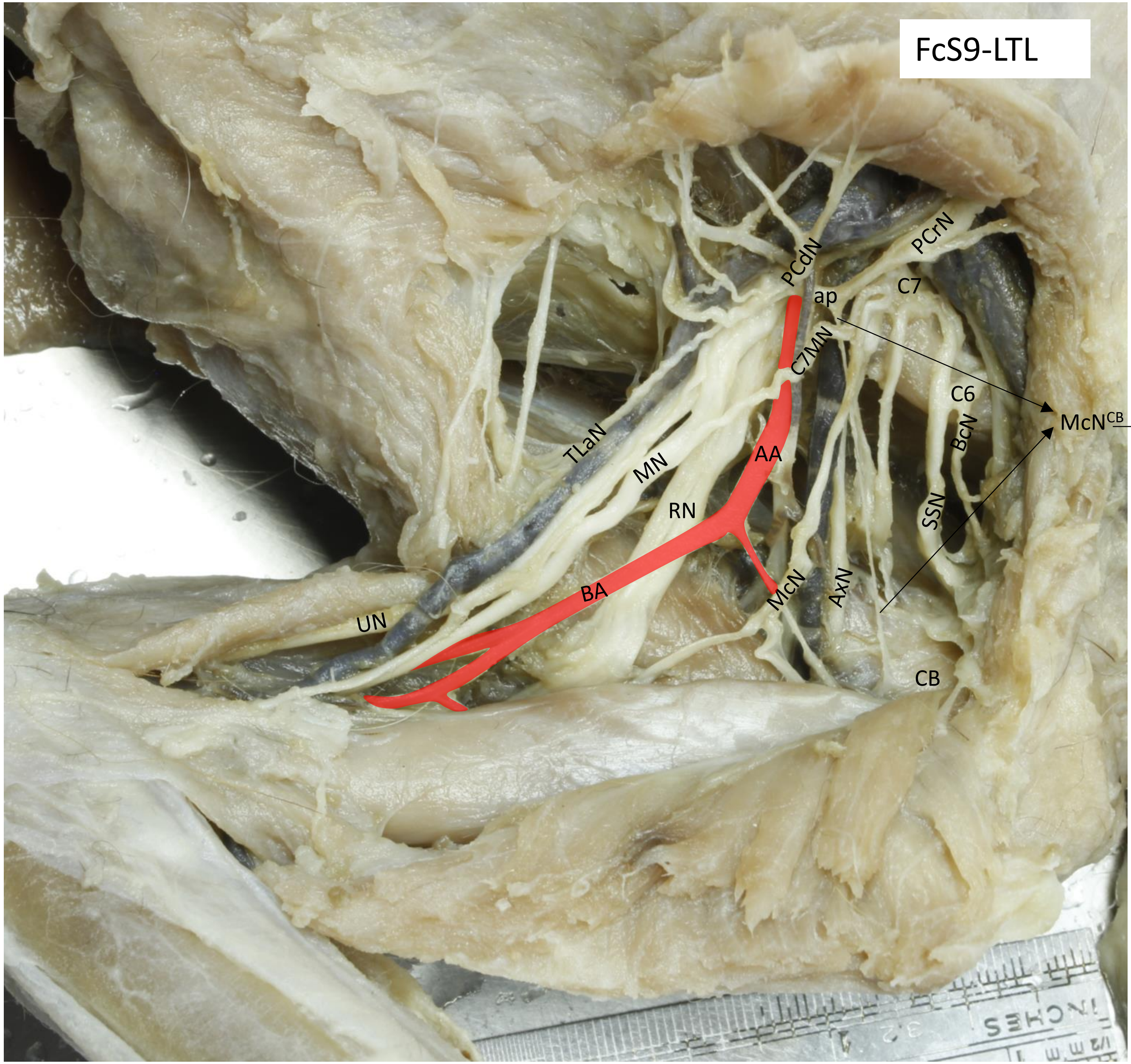

This branch originated from ap (C8)

4.15

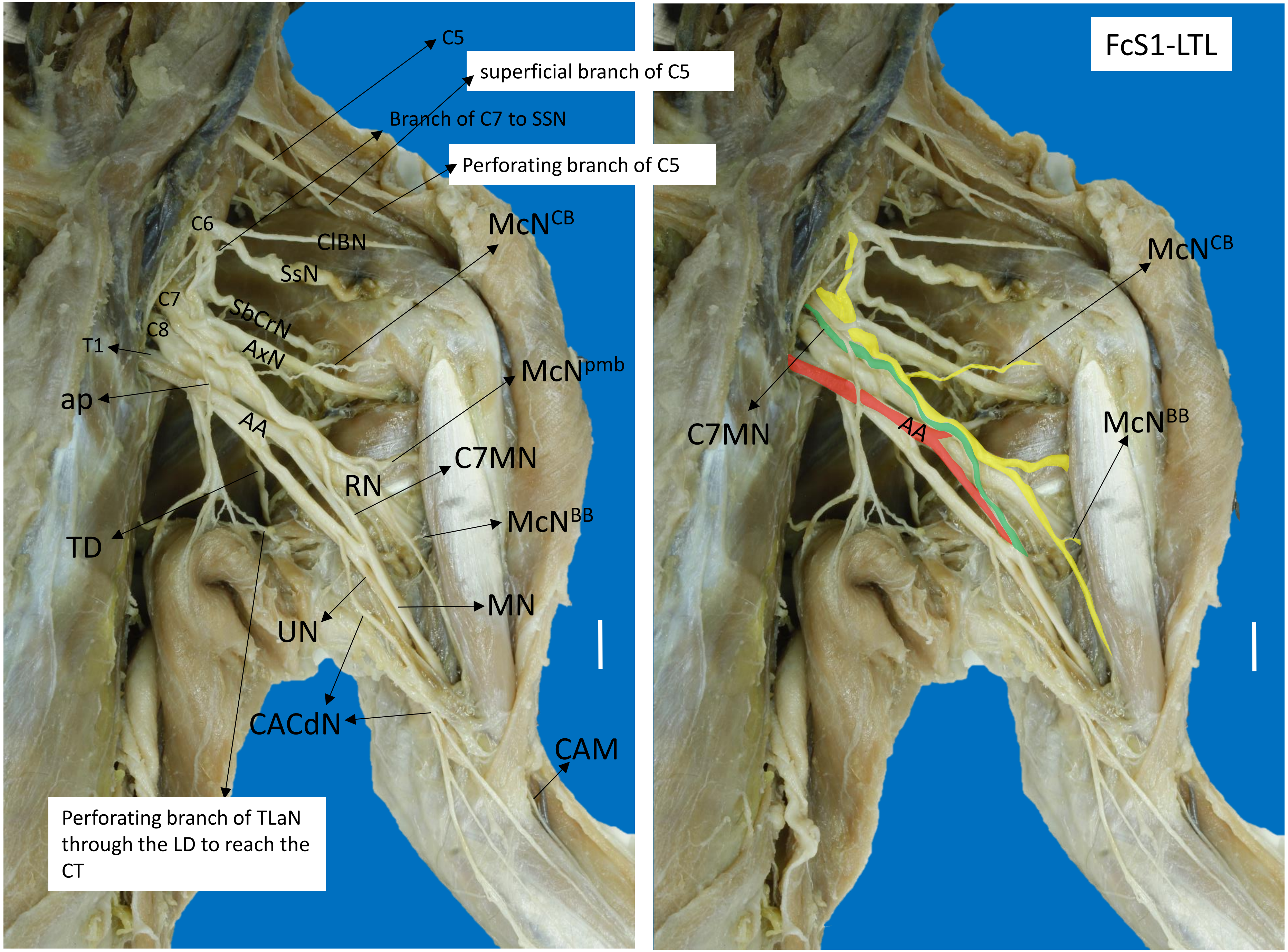
C5
superficial branch of C5
Branch of C7 to SSN
Perforating branch of C5
C6
ClBN
SsN
McNCB
C7
SbCrN
C8
T1
AxN
McNpmb
ap
AA
C7MN
RN
TD
McNBB
MN
UN
CACdN
CAM
Perforating branch of TLaN through the LD to reach the CT
FcS1-LTL
McNCB
C7MN
AA
McNBB
